# Supplementary material for: Spatial distribution of three ARGONAUTEs regulates the anther phasiRNA pathway
Source: Nat Commun. 2023 Jun 7;14:3333. doi: 10.1038/s41467-023-38881-z (PMC10247740; doi:10.1038/s41467-023-38881-z)
Supplement: Supplementary file 1 — Supplementary Information [file 41467_2023_38881_MOESM1_ESM.pdf]

Supplementary Figure 1

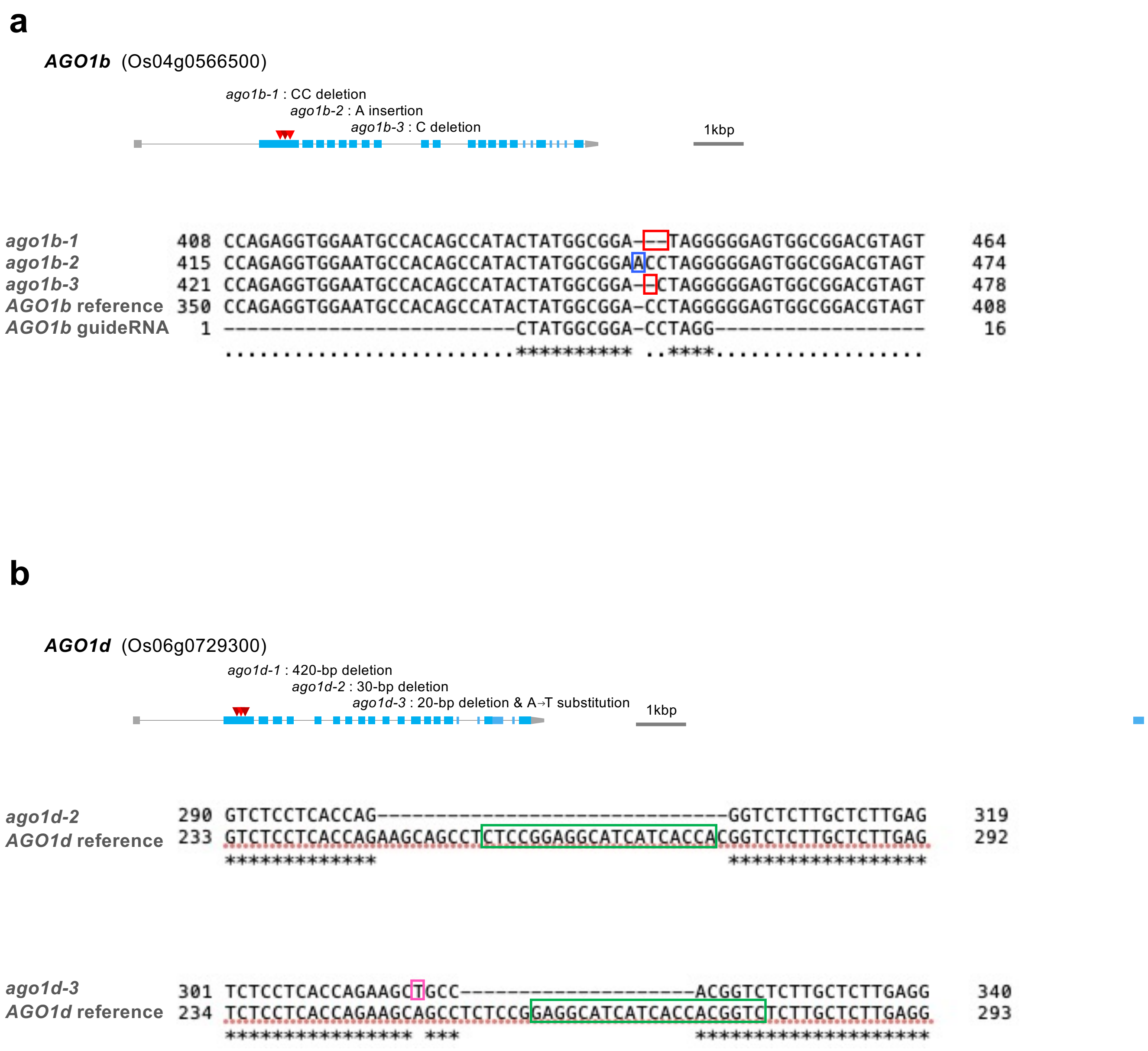

Supplementary Figure 1. a. Schematic structure and alignment of the *AGO1b* gene and its mutants. We generated three mutant *ago1b* alleles. Three red triangles represent the deletion or insertion sites of *ago1b-1*, *ago1b-2*, and *ago1b-3*. Red boxes show deletions, and the blue box shows an insertion. **b**. Schematic structure and alignment of the *AGO1d* gene and two of its mutants. We obtained three mutant *ago1d* alleles. Three red triangles represent the deletion or substitution sites of *ago1d-1*, *ago1d-2*, and *ago1d-3*. The green box shows the sequence of a guide RNA.

Supplementary Figure 2

a

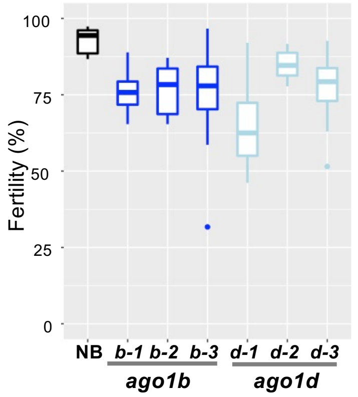

b

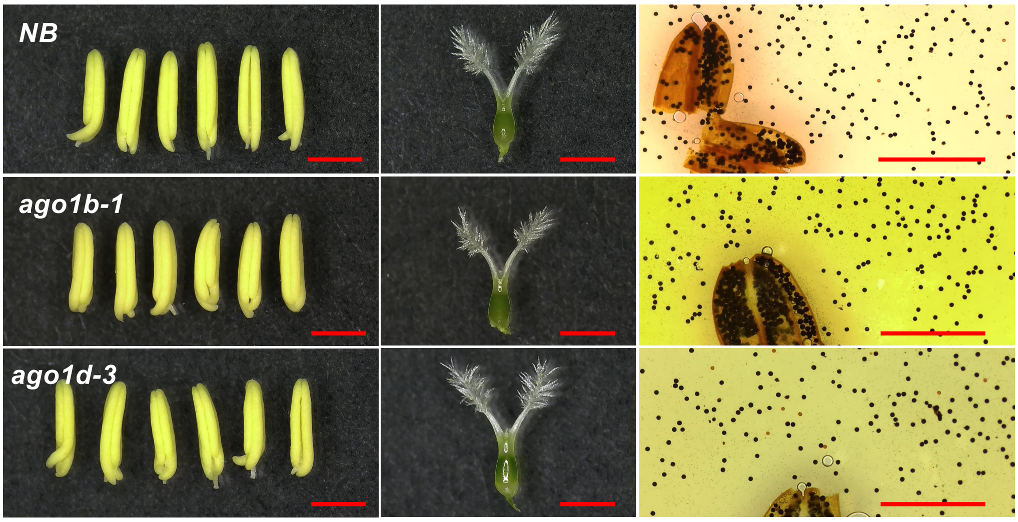

Scale bar:1 mm

Supplementary Figure 2. a. Fertility of Nipponbare (NB), *ago1b-1* (*b-1*), *ago1b-2* (*b-2*), *ago1b-3* (*b-3*), *ago1d-1* (*d-1*), *ago1d-2* (*d-2*), and *ago1d-3* (*d-3*). In each box plot, the centre line indicates the median and the edges of the box represent the first and third quartiles. Whiskers indicate 1.5× the interquartile range. Points are plotted as outliers. *n* = 8 panicles from 3 plants (NB), 11 panicles from 3 plants (*b-1*), 8 panicles from 3 plants (*b-2)*, 16 panicles from 3 plants (*b-3*), 19 panicles from 3 plants (*d-1*), 7 panicles from 3 plants (*d-2*), and 18 panicles from 3 plants (*d-3*). Source data are provided with this paper. **b**. Mature anthers (left), pistil (middle), and pollen grains (right) of Nipponbare (control), *ago1b-1*, and *ago1d-3*. The single mutants, *ago1b-1* and *ago1d-3*, showed normal anthers and pistils compared to those of Nipponbare. Furthermore, mature pollen grains of *ago1b-1* and *ago1d-3* were also stained with iodine-potassium iodide. The pollen staining was performed five times for NB, twelve times for *ago1b-1*, and seven times for *ago1d-3* with similar results.

Supplementary Figure 3

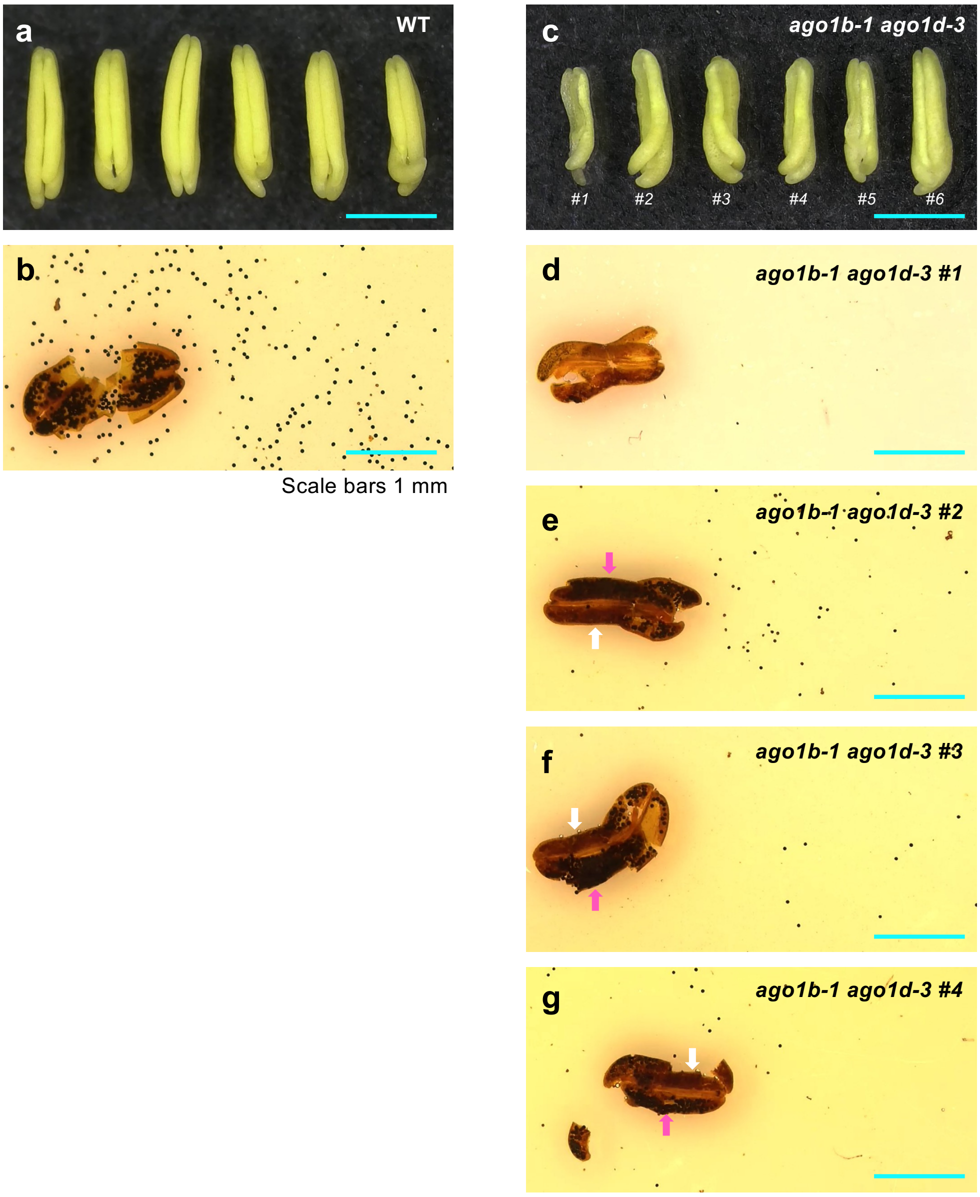

Supplementary Figure 3. a. Anthers of WT. **b**. Starch staining of pollen from WT anthers. The WT pollen staining was performed four times with similar results. **c**. Anthers of *ago1b-1 ago1d-3* double mutants with several abnormal shapes from severe to mild types (#1–6). **d–g**. Starch staining of pollen from severely abnormal and semi-abnormal anthers of the *ago1b-1 ago1d-3* double mutant (#1–4). Non-staining pollens reflect an abnormality of pollen activity and development (**d**). Semi-abnormal anthers of the double mutant contain non-staining pollens (white arrows) as well as stained pollen grains (magenta arrows) (**e, f,** and **g**). Most of the stained pollen grains were trapped in the anthers of the *ago1b-1 ago1d-3* double mutant (**e, f,** and **g,** magenta arrows), perhaps due to defects of somatic anther wall development. The pollen staining of the *ago1b-1 ago1d-3* double mutant was performed four times with similar results. Scale bars are 1 mm.

Supplementary Figure 4

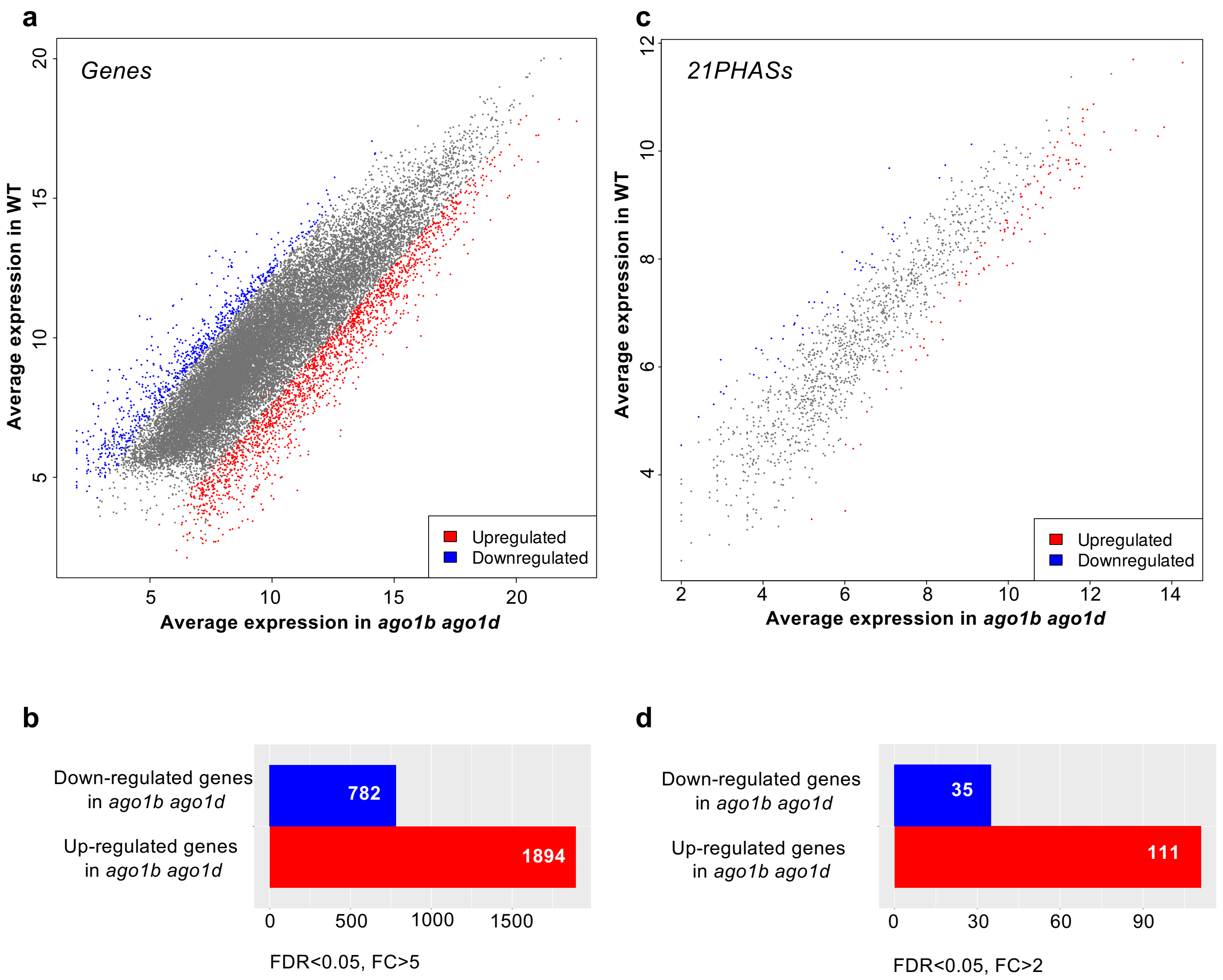

Supplementary Figure 4. Differentially expressed genes/*21PHAS*s in WT and *ago1b/d* double mutants. a, c. Scatter plots of differentially expressed genes/*21PHAS*s in *ago1b/d* double mutants compared to those in WT. **b.** Blue bar indicates 782 downregulated genes, and red bar indicates 1,894 upregulated genes, in mutants. **d.** Thirty-five lncRNAs derived from *21PHAS*s were downregulated, and 111 lncRNAs derived from *21PHAS*s were upregulated, in the double mutants (**d**). Three or two biological replicates were prepared for RNA sequences of WT or *ago1b-1 ago1d-3* double mutant, respectively. FDR, false discovery rate; FC, fold change.

Supplementary Figure 5

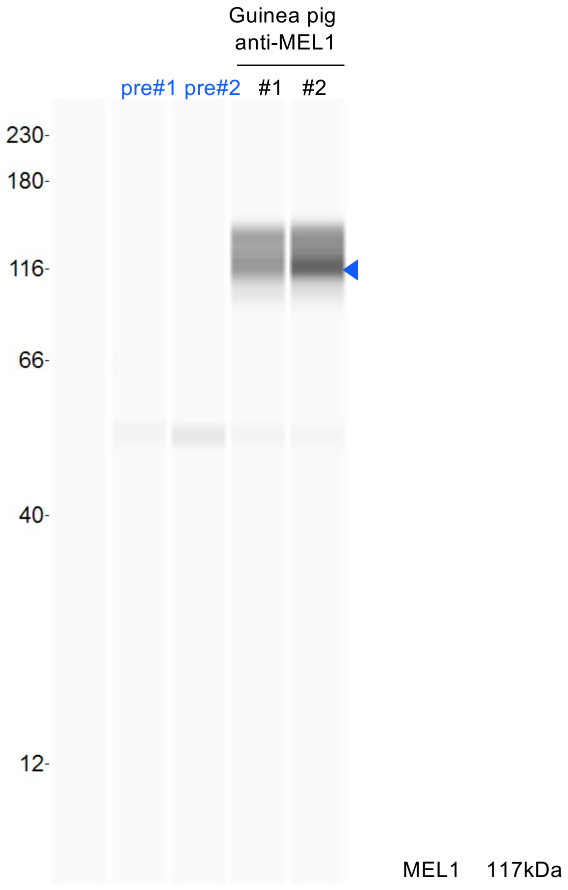

Supplementary Figure 5. Wes analysis of anthers using guinea pig anti-MEL1 antibody. Total proteins were extracted from 0.5-mm anthers during early meiosis when MEL1 is abundantly expressed. Wes signals coincide with the predicted molecular mass of MEL1 (blue arrowhead), demonstrating that the guinea pig anti-MEL1 antibody detects MEL1 protein. The wes analysis was performed twice with similar results.

Supplementary Figure 6

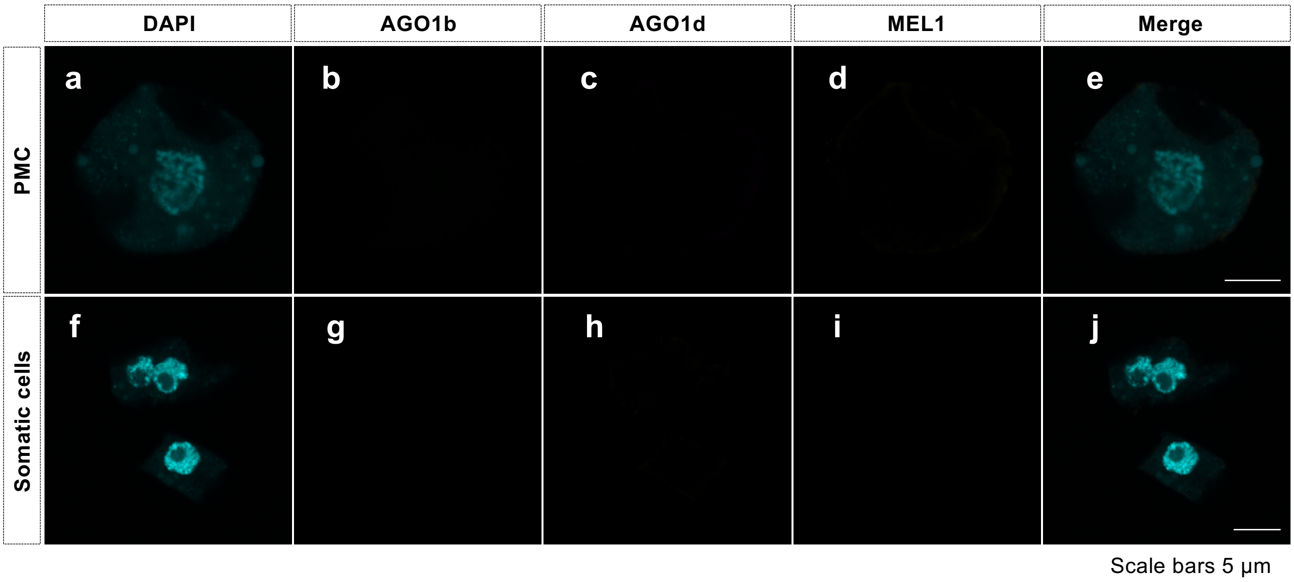

Supplementary Figure 6. Immunoimaging for negative control of Figure 5. **a–j.** Samples were treated with 0.3% BSA buffer during the primary antibody reaction, while the 2D-immunoimaging samples for Figure 5 were treated with anti-AGO1b, -AGO1d, and -MEL1 antibodies. All procedures, with the exception of the primary antibody reaction, were performed with the samples shown in Figure 5. DAPI signals (cyan) were detected in both PMCs and somatic anther wall cells (**a, f**). However, AGO1d (**b, g**), AGO1b (**c, h**), and MEL1 (**d, i**) signals were rarely detected in the same image-capturing conditions as in Figure 5 using LSM 880 with Airyscan. Merged images of a, b, c, d (**e**) and f, g, h, i (**j**). The 2D-immunoimaging for negative control using secondary antibodies (Alexa 488 anti-mouse and Alexa 568 anti-rabbit) was repeated with similar results.

Supplementary Figure 7

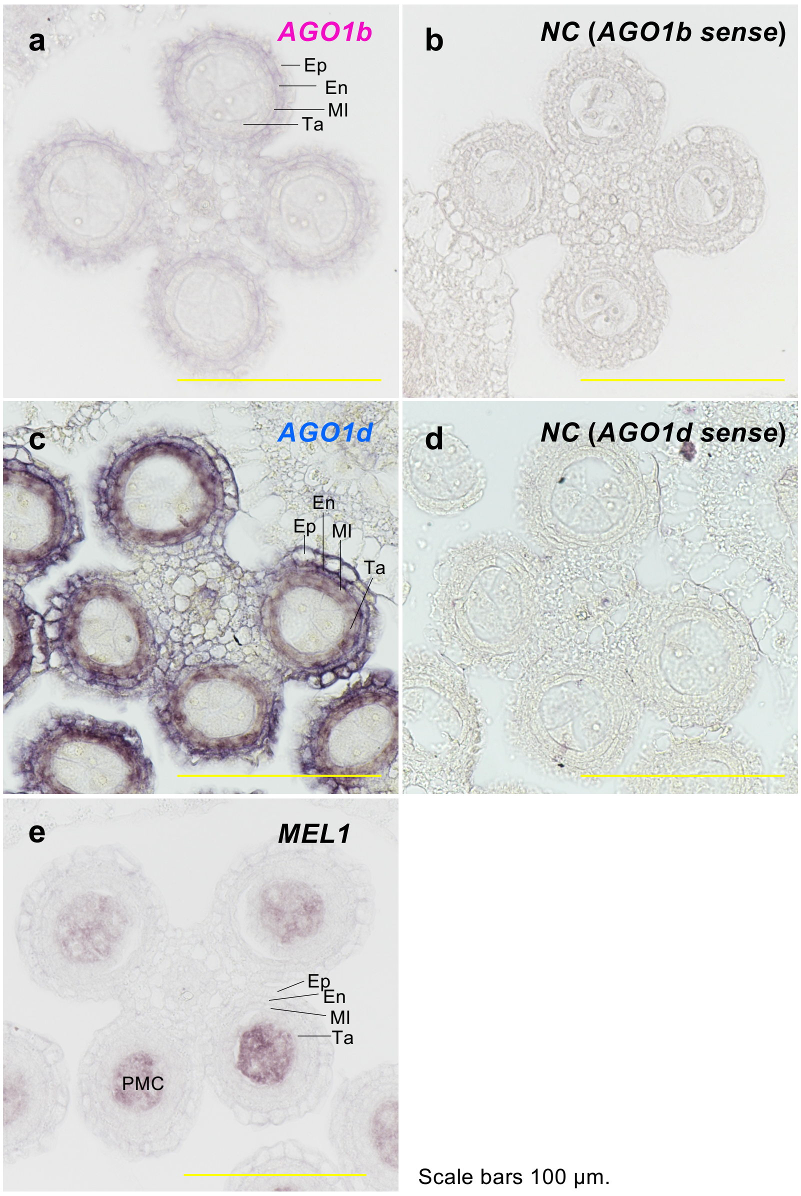

Scale bars 100 μm.

Supplementary Figure 7. *In situ* hybridization of *AGO1b, AGO1d, MEL1*, and negative control (NC, sense strand) probes using anthers from 2.0–2.5-mm inflorescences. *AGO1b* localization was detected at the anther wall, especially the endothecium (En) and middle layer (Ml), while there was almost no signal in the negative control using the *AGO1b* sense probe (**a, b**). *AGO1d* localization was also detected at the anther wall, especially the tapetum layer (Ta), while there was almost no signal in the negative control using the *AGO1d* sense probe (**c, d**). *MEL1* mRNA is enriched in the PMCs, contrary to the *AGO1b/d* localization (**e**). The *in situ* hybridization was performed five times for *AGO1b*, *AGO1b sense, AGO1d* and *MEL1*, and four times for *AGO1d sense* with similar results.
