# Supplementary material for: Spatial distribution of three ARGONAUTEs regulates the anther phasiRNA pathway
Source: Nat Commun. 2023 Jun 7;14:3333. doi: 10.1038/s41467-023-38881-z (PMC10247740; doi:10.1038/s41467-023-38881-z)
Supplement: Supplementary file 3 — Description of Additional Supplementary Files [file 41467_2023_38881_MOESM3_ESM.pdf]

Description of Additional Supplementary Files:

Supplementary Data 1. Transcriptome from anthers of WT or ago1b ago1d.

Supplementary Data 2. Developmental stages of anthers.

Supplementary Data 3. Mass spectrometry of IP fractions with anti-AGO1b (rabbit), AGO1d (rabbit), and AGO1d (mouse).

Supplementary Data 4. Small RNAs deep-sequenced and mapped on the rice genome.

Supplementary Data 5. The 21-nt phasiRNA clusters in AGO1b-RIP fractions.

Supplementary Data 6. The 21-nt phasiRNA clusters in AGO1d-RIP fractions.

Supplementary Data 7. The 24-nt phasiRNA clusters in AGO1d-RIP fractions.

Supplementary Data 8. The 24-nt phasiRNA clusters in AGO1d-RIP fractions.

Supplementary Data 9. Mass spectrometry of IP fractions with anti-MEL1#1 (guinea pig) by Kazusa DNA Res. Inst.

Supplementary Data 10. Primer sequences.

Supplementary Movie 1. 3D movie of AGO1b/d immunostaining using whole anthers. Cyan signals indicate DAPI staining. Magenta signals indicate the indirect fluorescence of the AGO1b protein. Green signals indicate the indirect fluorescence of the AGO1d protein. The 0.5 mm-long anthers at early meiosis were used for 3D-multiple immunoimaging. The upper right figure shows a cross-section (X section) of the 3D anther immunoimaging, in which 0.5-mm anthers from the early meiosis stage were used. Laser excitation/emission are 405 nm/410–455 nm for DAPI, 488 nm/490–552 nm for AGO1d, and 561 nm/544–615 nm for AGO1b.
