# Supplementary material for: Spatial distribution of three ARGONAUTEs regulates the anther phasiRNA pathway
Source: Nat Commun. 2023 Jun 7;14:3333. doi: 10.1038/s41467-023-38881-z (PMC10247740; doi:10.1038/s41467-023-38881-z)
Supplement: Supplementary file 15 — Reporting Summary [file 41467_2023_38881_MOESM15_ESM.pdf]

nature portfolio

Corresponding author(s): Reina Komiya

Last updated by author(s): May 11, 2023

Reporting Summary

Nature Portfolio wishes to improve the reproducibility of the work that we publish. This form provides structure for consistency and transparency in reporting. For further information on Nature Portfolio policies, see our Editorial Policies and the Editorial Policy Checklist.

Statistics

For all statistical analyses, confirm that the following items are present in the figure legend, table legend, main text, or Methods section.

n/a	Confirmed	
☐	☒	The exact sample size (*n*) for each experimental group/condition, given as a discrete number and unit of measurement
☐	☒	A statement on whether measurements were taken from distinct samples or whether the same sample was measured repeatedly
☐	☒	The statistical test(s) used AND whether they are one- or two-sided<br>*Only common tests should be described solely by name; describe more complex techniques in the Methods section.*
☒	☐	A description of all covariates tested
☐	☒	A description of any assumptions or corrections, such as tests of normality and adjustment for multiple comparisons
☐	☒	A full description of the statistical parameters including central tendency (e.g. means) or other basic estimates (e.g. regression coefficient) AND variation (e.g. standard deviation) or associated estimates of uncertainty (e.g. confidence intervals)
☐	☒	For null hypothesis testing, the test statistic (e.g. *F*, *t*, *r*) with confidence intervals, effect sizes, degrees of freedom and *P* value noted<br>*Give P values as exact values whenever suitable.*
☒	☐	For Bayesian analysis, information on the choice of priors and Markov chain Monte Carlo settings
☒	☐	For hierarchical and complex designs, identification of the appropriate level for tests and full reporting of outcomes
☒	☐	Estimates of effect sizes (e.g. Cohen's *d*, Pearson's *r*), indicating how they were calculated

Our web collection on statistics for biologists contains articles on many of the points above.

Software and code

Policy information about availability of computer code

Data collection: n/a

Data analysis:
- Trimmomatic-0.38
- tophat/2.1.1
- samtools/1.3.1
- PHASIS/3.3
- bowtie/1.0.0 (used by PHASIS)
- ggplot2/3.2.1
- IGV/2.5.2
- ZEN2012 (LSM780), ZEN2 (LSM880 Airyscan), ZEN2014(Lightsheet Z.1)
- HISAT2/2.2.0
- Imaris/9.1.2

Microscopy
1.Carl Zeiss Lightsheet Z.1
20x/1.0 PlanApochomat detection lens,
10x/0.2 LSFM Clearing illumination lens

2. Nikon NiE

DS-Ri2 Color Camera
40x/0.95 Plan Apoα lens, 20x/0.75 Plan Apo α lens

3. Carl Zeiss LSM780
40x/1.40 Plan Apochromat lens (Movie1) and 63x/1.46 α-Plan Apochromat lens

4. Carl Zeiss LSM880 Airyscan
63x/1.40 oil Plan Apochromat lens

5. Nikon SMZ745T
Motic Images Plus 2.3S

Wes
Protein simple Wes
Compass for SW ver.3.1.7

qPCR
TAKARA Thermal Cycler Dice Real time System Ⅲ
ver. 6. 01D

For manuscripts utilizing custom algorithms or software that are central to the research but not yet described in published literature, software must be made available to editors and reviewers. We strongly encourage code deposition in a community repository (e.g. GitHub). See the Nature Portfolio guidelines for submitting code & software for further information.

Data

Policy information about availability of data

All manuscripts must include a data availability statement. This statement should provide the following information, where applicable:

- Accession codes, unique identifiers, or web links for publicly available datasets
- A description of any restrictions on data availability
- For clinical datasets or third party data, please ensure that the statement adheres to our policy

AGO1b: Os04g0566500; AGO1d: Os06g0729300; MEL1: Os03g0800200. The gene datasets used during the current study are available in the RAP-DB repository (https://rapdb.dna.affrc.go.jp/).

RIP data and RNA sequences have been deposited in the DNA Data Bank of Japan (DDBJ), under the accession codes, PRJDB13635 [https://ddbj.nig.ac.jp/resource/sra-submission/DRA015390] and PRJDB14859 [https://ddbj.nig.ac.jp/resource/sra-submission/DRA015421].

Imaging data have been deposited in the BioStudies, under the accession code, S-BSST1083 [https://www.ebi.ac.uk/biostudies/studies/S-BSST1083?key=8d7214cd-b5eb-4985-8e73-1eab99a5ccbc]. Source data are provided in this paper.

Human research participants

Policy information about studies involving human research participants and Sex and Gender in Research.

Reporting on sex and gender	n/a
Population characteristics	n/a
Recruitment	n/a
Ethics oversight	n/a

Note that full information on the approval of the study protocol must also be provided in the manuscript.

Field-specific reporting

Please select the one below that is the best fit for your research. If you are not sure, read the appropriate sections before making your selection.

[x] Life sciences [ ] Behavioural & social sciences [ ] Ecological, evolutionary & environmental sciences

For a reference copy of the document with all sections, see nature.com/documents/nr-reporting-summary-flat.pdf

Life sciences study design

All studies must disclose on these points even when the disclosure is negative.

Sample size	No sample size calculation was performed. Sample size was chosen according to the standard in the plant biology field.
Data exclusions	n/a
Replication	All the experiments were reproducible in the repeated experiments. Replicate numbers are described in the manuscript.
Randomization	Plant samples were placed randomly in the plant facility.
Blinding	No blinding was applied for sampling.

Reporting for specific materials, systems and methods

We require information from authors about some types of materials, experimental systems and methods used in many studies. Here, indicate whether each material, system or method listed is relevant to your study. If you are not sure if a list item applies to your research, read the appropriate section before selecting a response.

Materials & experimental systems

n/a	Involved in the study	
☐	☒	Antibodies
☒	☐	Eukaryotic cell lines
☒	☐	Palaeontology and archaeology
☒	☐	Animals and other organisms
☒	☐	Clinical data
☒	☐	Dual use research of concern

Methods

n/a	Involved in the study	
☒	☐	ChIP-seq
☒	☐	Flow cytometry
☒	☐	MRI-based neuroimaging

Antibodies

Antibodies used

1) AGO1b
A synthetic peptide, AGO1b (Cys-GSSQRAERGPQQH-OH), was used to raise rabbit polyclonal antibody against rice AGO1b. The rabbit antisera were purified using affinity chromatography (SCRUM Inc.).

(2) AGO1d
Two oligopeptides, AGO1d-1 (Cys-GRGSYYPQAQQYH-OH) and AGO1d-2 (Cys-HQQPYNSSVRPQH-OH), were used to raise antibodies in rabbits and mice. The rabbit antisera were purified using affinity chromatography (SCRUM Inc.).

(3)
Two oligopeptides, MEL1-N (CVYGAPMPAAHHQGAYQ) and MEL1-C (GQAVAREGPVEVRQLPKC) were used to induce antibody production in guinea pigs. The guinea pig antiserum were purified using affinity chromatography (SCRUM Inc.). The same oligopeptides, MEL1-N (CVYGAPMPAAHHQGAYQ) and MEL1-C (GQAVAREGPVEVRQLPKC), were used to induce antibody production in rabbits and validated in Mass spectrometry analysis (Komiya et al., 2014).

Animals were immunized five times with keyhole limpet hemocyanin–peptide conjugates mixed with Freund's complete adjuvant, and were bled seven days after the last immunization for (1), (2), and (3). Antiserum was purified on a peptide coupling purification column. SCRUM Inc. performed all procedures for antibody generation. The animal experimentation committee of SCRUM Inc. follows its own established guidelines for animal care. SCRUM Inc. referenced to the Ministry of the Environment guidelines [https://elaws.e-gov.go.jp/document?lawid=418M60001000001] and [https://www.env.go.jp/nature/dobutsu/aigo/2_data/nt_h180428_88.html] for its own animal care guidelines.

(4) Secondary antibody
Goat anti-Rabbit IgG (H+L) Highly Cross-Adsorbed Secondary Antibody, Alexa Fluor 568 (Invitrogen, A11036)
Goat anti-Mouse IgG (H+L) Highly Cross-Adsorbed Secondary Antibody, Alexa Fluor 488 (Invitrogen, A11001)
Goat anti-Guinea pig IgG HL, Alexa FluorR 647 (abcom, ab150187)

Validation

(1) AGO1b
Mass spectrometry analysis confirmed that AGO1b was immunopurified in AGO1b-RIP fractions, using antibodies generated in rabbits (Supplementary Table 3).

(2) AGO1d
Mass spectrometry analysis confirmed that AGO1d was immunopurified in AGO1d-RIP fractions, using antibodies generated in rabbits and mice, respectively (Supplementary Table 3).

(3) MEL1
Mass spectrometry analysis confirmed that MEL1 was immunopurified in MEL1(#1)-RIP fractions, using the antibody generated in guinea pigs (Supplementary Table 9).
Automated western analysis using Wes, with MEL1 antibodies (guinea pigs), revealed that the predicted molecular masses of MEL1 were confirmed at the early meiotic stage (Supplementary Fig. 5). Furthermore, immunostaining against MEL1 showed cytoplasmic MEL1 in germ cells. Thus molecular weight and cellular and subcellular localization using anti-guinea pigs MEL1 antibodies are similar to those of anti-rabbit MEL1 antibodies.
