# Supplementary figures and images for: Spatial distribution of three ARGONAUTEs regulates the anther phasiRNA pathway
Source: Nat Commun. 2023 Jun 7;14:3333. doi: 10.1038/s41467-023-38881-z (PMC10247740; doi:10.1038/s41467-023-38881-z)

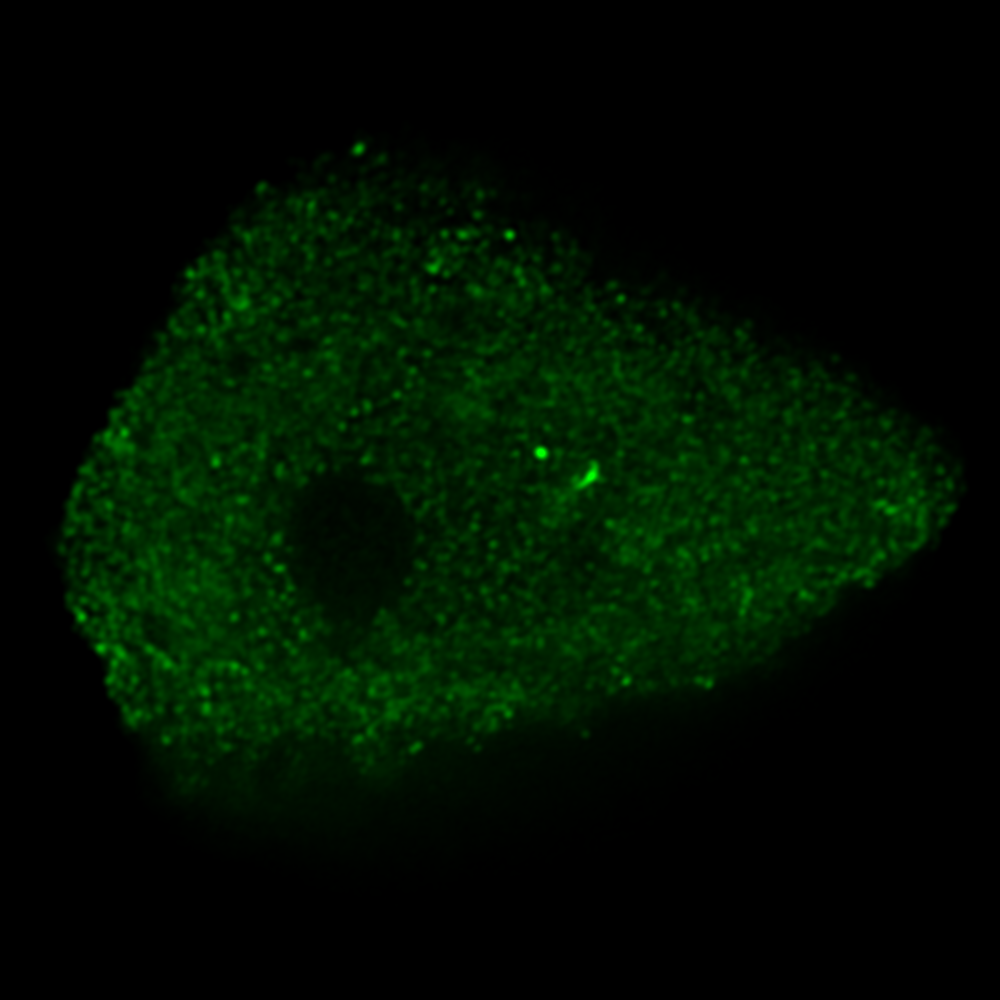

Supplement: Supplementary file 16 — Source_Data_zip [file 41467_2023_38881_MOESM16_ESM.zip › Source_Data_files_RK/Fig_5/Fig5_e-h_221031 NB SD 0.5A DAPI 1D-488 1B-561 MEL1affy-647 PMC-12z_Airyscan Processing-13/221031 NB SD 0.5A DAPI 1D-488 1B-561 MEL1affy-647 PMC-12z_Airyscan Processing-13_z13c3.tif]

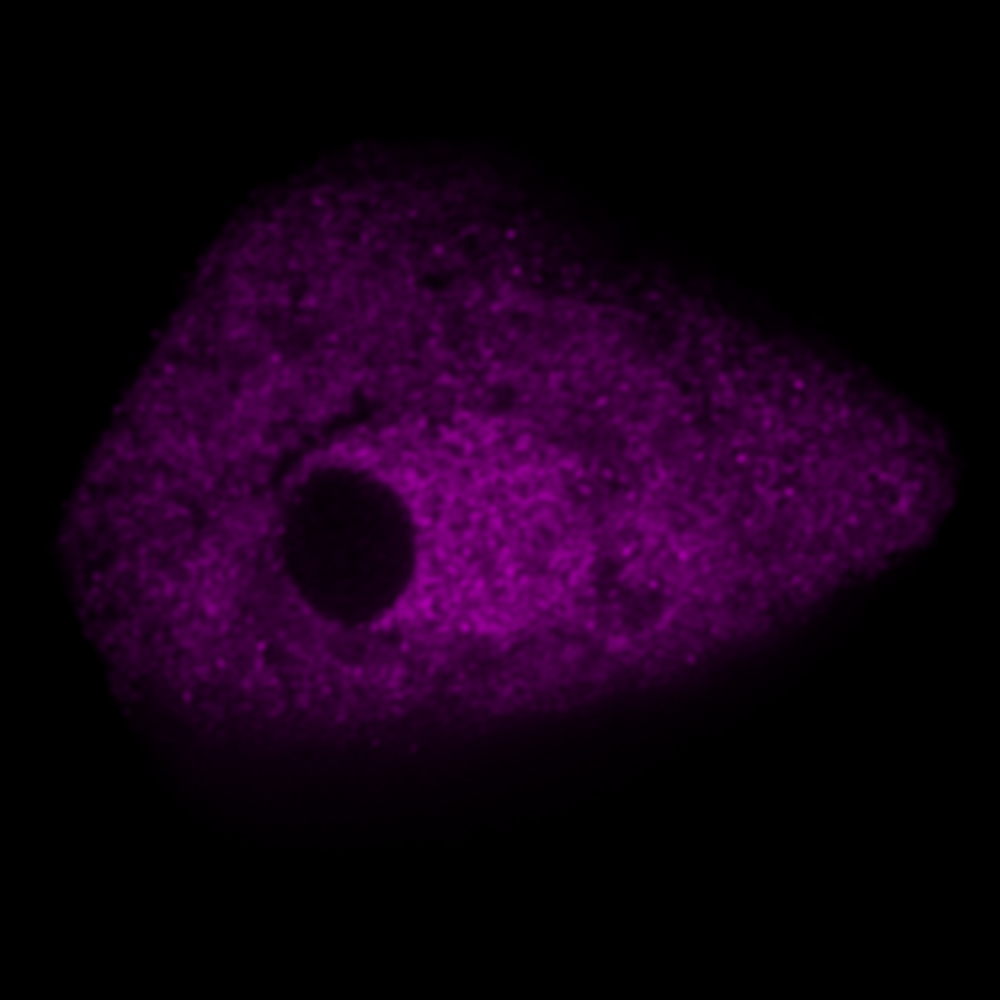

Supplement: Supplementary file 16 — Source_Data_zip [file 41467_2023_38881_MOESM16_ESM.zip › Source_Data_files_RK/Fig_5/Fig5_e-h_221031 NB SD 0.5A DAPI 1D-488 1B-561 MEL1affy-647 PMC-12z_Airyscan Processing-13/221031 NB SD 0.5A DAPI 1D-488 1B-561 MEL1affy-647 PMC-12z_Airyscan Processing-13_z13c2.tif]

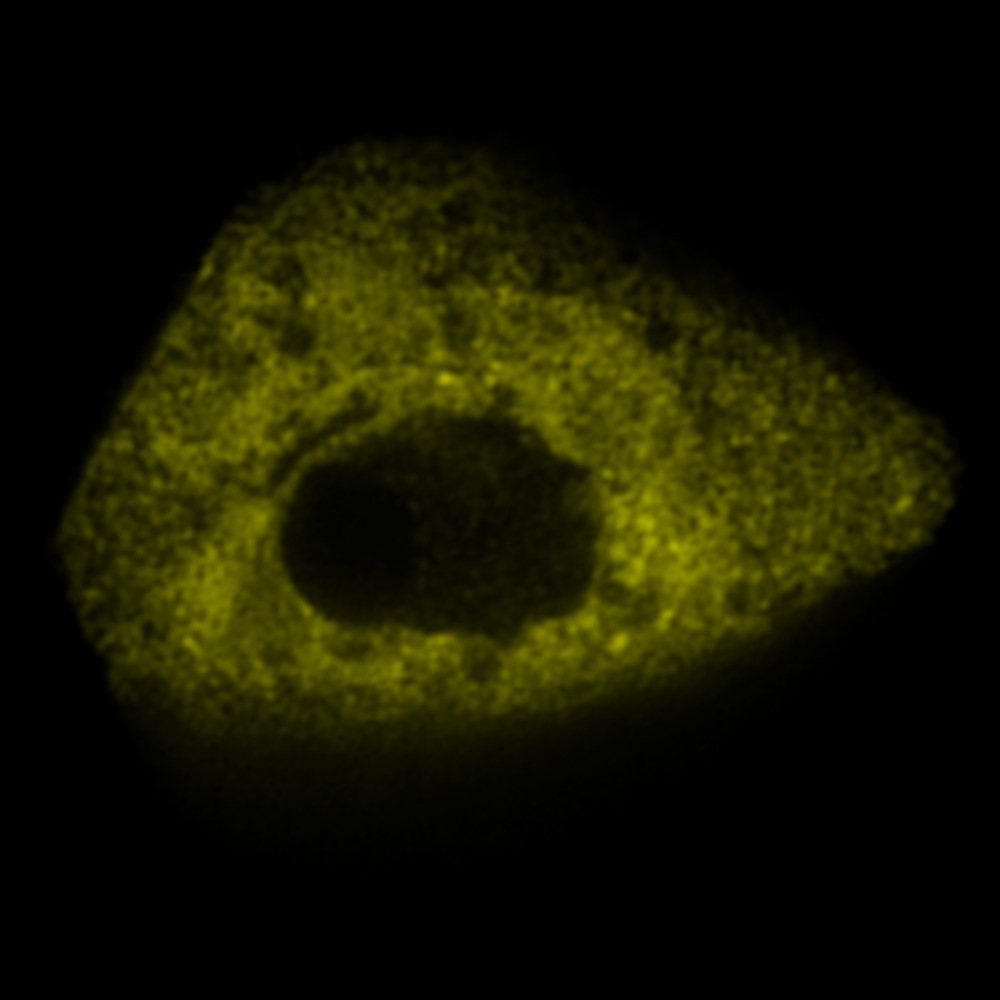

Supplement: Supplementary file 16 — Source_Data_zip [file 41467_2023_38881_MOESM16_ESM.zip › Source_Data_files_RK/Fig_5/Fig5_e-h_221031 NB SD 0.5A DAPI 1D-488 1B-561 MEL1affy-647 PMC-12z_Airyscan Processing-13/221031 NB SD 0.5A DAPI 1D-488 1B-561 MEL1affy-647 PMC-12z_Airyscan Processing-13_z13c1.tif]

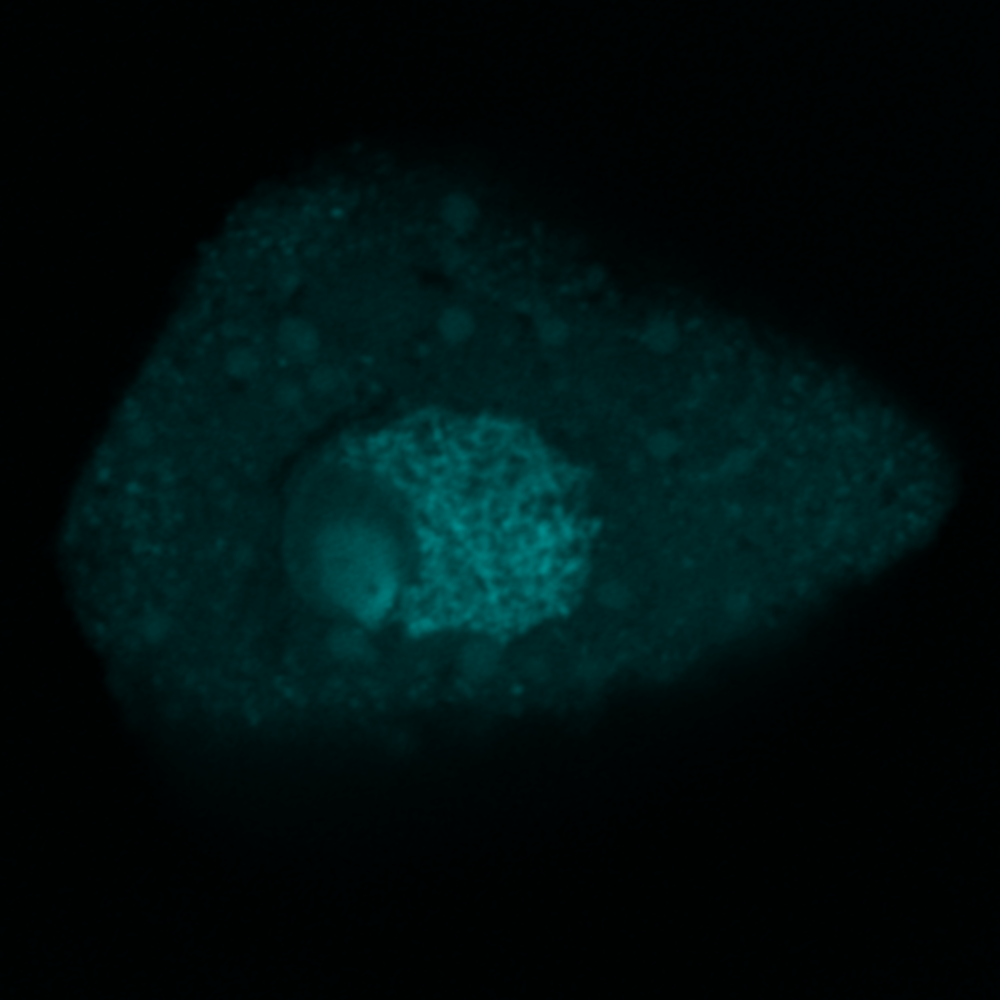

Supplement: Supplementary file 16 — Source_Data_zip [file 41467_2023_38881_MOESM16_ESM.zip › Source_Data_files_RK/Fig_5/Fig5_e-h_221031 NB SD 0.5A DAPI 1D-488 1B-561 MEL1affy-647 PMC-12z_Airyscan Processing-13/221031 NB SD 0.5A DAPI 1D-488 1B-561 MEL1affy-647 PMC-12z_Airyscan Processing-13_z13c4.tif]

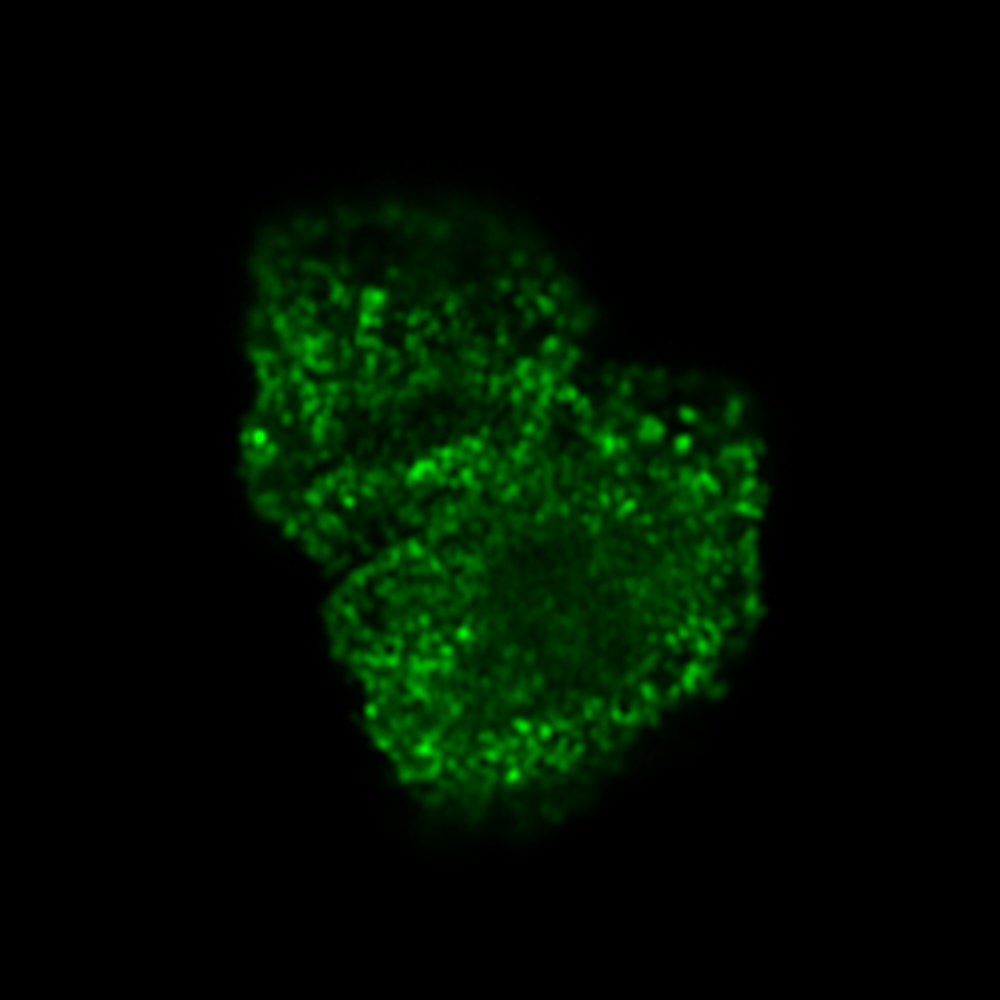

Supplement: Supplementary file 16 — Source_Data_zip [file 41467_2023_38881_MOESM16_ESM.zip › Source_Data_files_RK/Fig_5/Fig5_1-d_221031 NB SD 0.5A DAPI 1D-488 1B-561 MEL1affy-647 SC-11_Airyscan Processing-part2/221031 NB SD 0.5A DAPI 1D-488 1B-561 MEL1affy-647 SC-11_Airyscan Processing-part2_c3.tif]

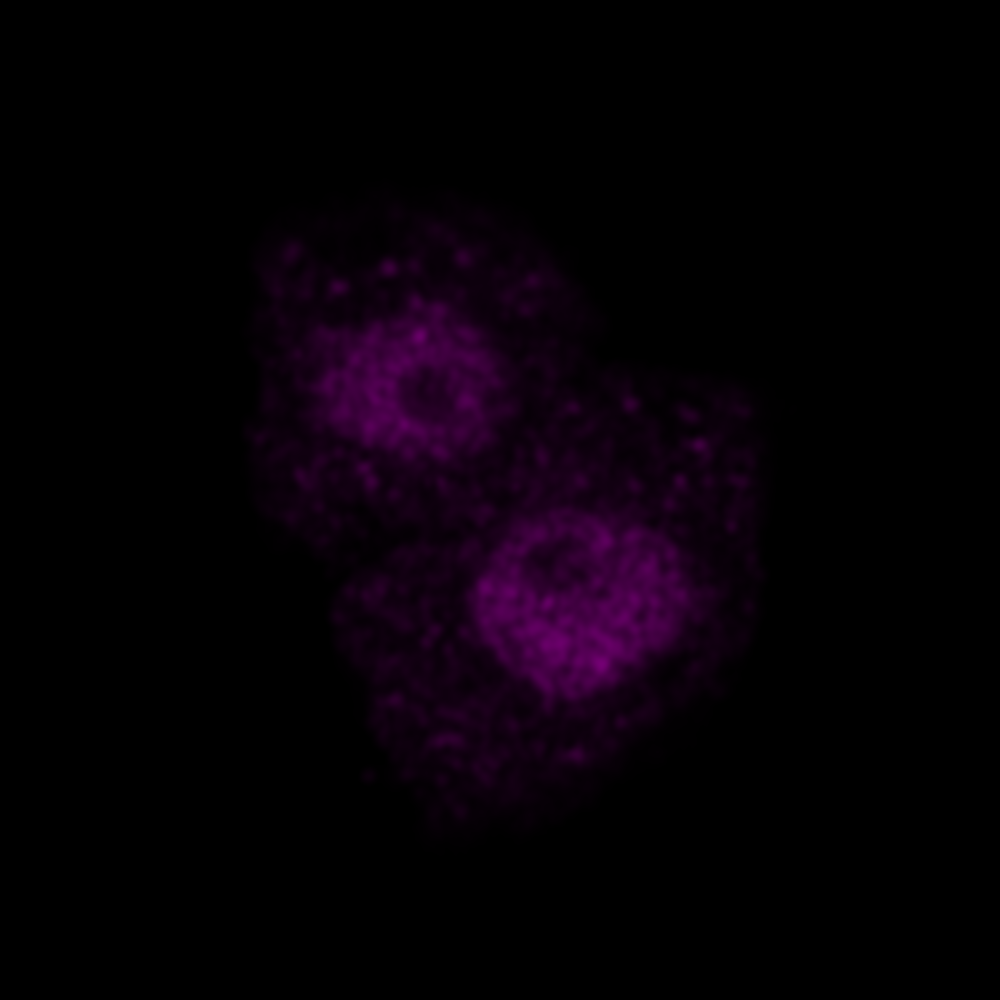

Supplement: Supplementary file 16 — Source_Data_zip [file 41467_2023_38881_MOESM16_ESM.zip › Source_Data_files_RK/Fig_5/Fig5_1-d_221031 NB SD 0.5A DAPI 1D-488 1B-561 MEL1affy-647 SC-11_Airyscan Processing-part2/221031 NB SD 0.5A DAPI 1D-488 1B-561 MEL1affy-647 SC-11_Airyscan Processing-part2_c2.tif]

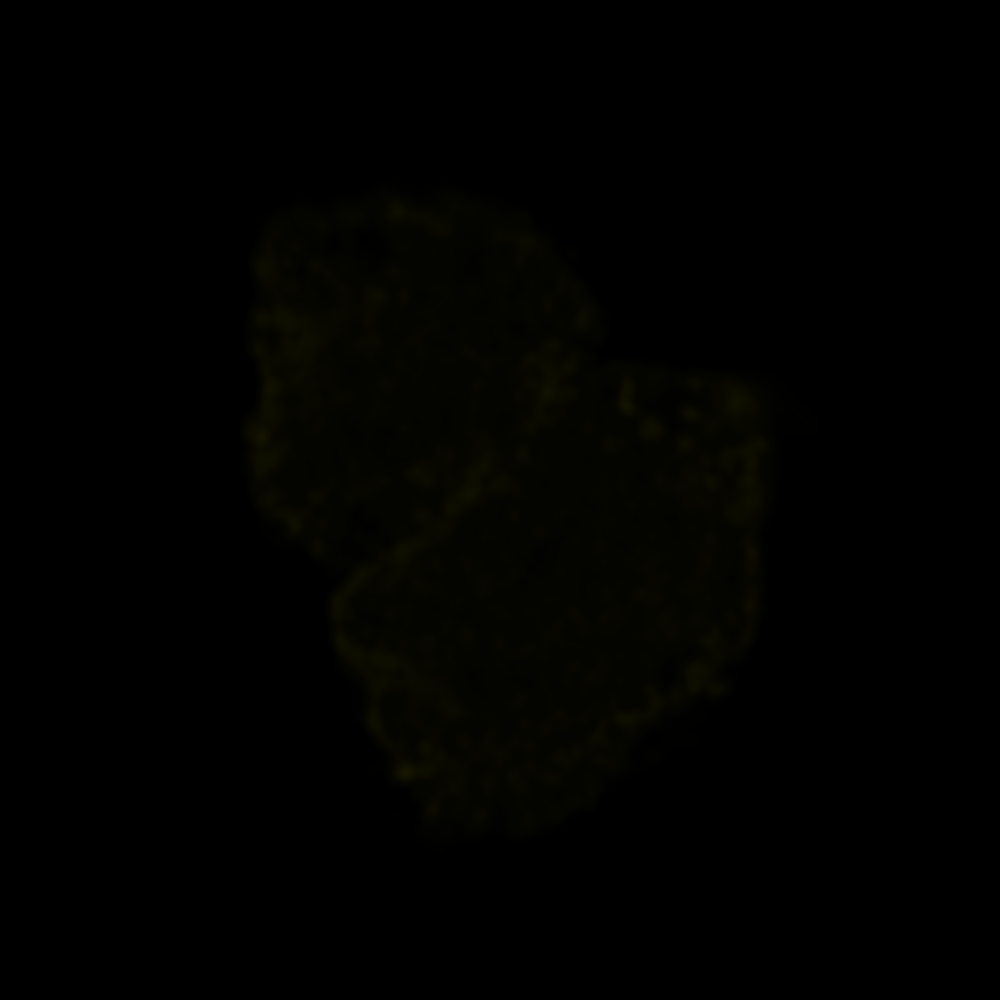

Supplement: Supplementary file 16 — Source_Data_zip [file 41467_2023_38881_MOESM16_ESM.zip › Source_Data_files_RK/Fig_5/Fig5_1-d_221031 NB SD 0.5A DAPI 1D-488 1B-561 MEL1affy-647 SC-11_Airyscan Processing-part2/221031 NB SD 0.5A DAPI 1D-488 1B-561 MEL1affy-647 SC-11_Airyscan Processing-part2_c1.tif]

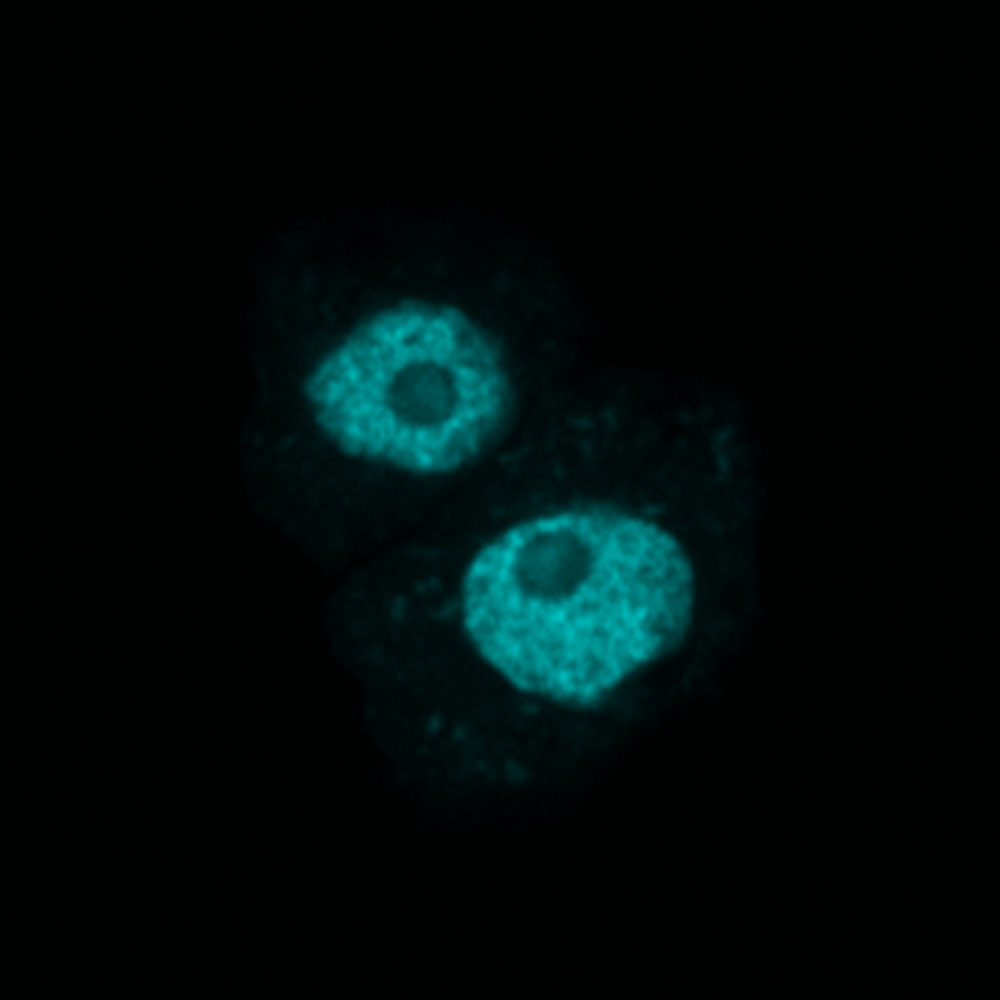

Supplement: Supplementary file 16 — Source_Data_zip [file 41467_2023_38881_MOESM16_ESM.zip › Source_Data_files_RK/Fig_5/Fig5_1-d_221031 NB SD 0.5A DAPI 1D-488 1B-561 MEL1affy-647 SC-11_Airyscan Processing-part2/221031 NB SD 0.5A DAPI 1D-488 1B-561 MEL1affy-647 SC-11_Airyscan Processing-part2_c4.tif]

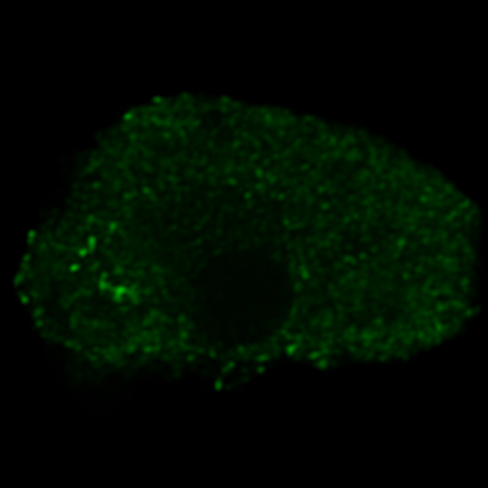

Supplement: Supplementary file 16 — Source_Data_zip [file 41467_2023_38881_MOESM16_ESM.zip › Source_Data_files_RK/Fig_5/Fig5_i-l_221020 NB SD AGO1b-568 AGO1d-488 DAPI PMC-1z_Airyscan Processing-34-part3-2/221020 NB SD AGO1b-568 AGO1d-488 DAPI PMC-1z_Airyscan Processing-34-part3-2_z34_ChA-T2.tif]

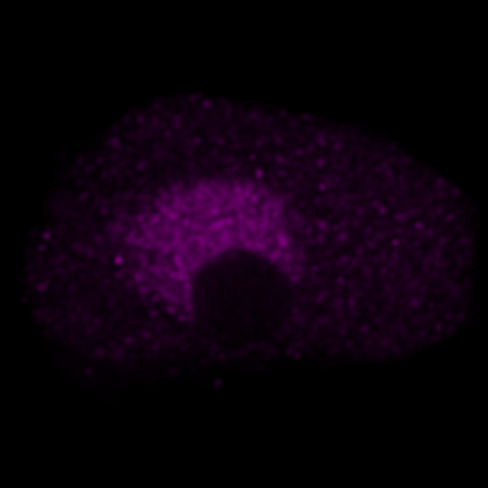

Supplement: Supplementary file 16 — Source_Data_zip [file 41467_2023_38881_MOESM16_ESM.zip › Source_Data_files_RK/Fig_5/Fig5_i-l_221020 NB SD AGO1b-568 AGO1d-488 DAPI PMC-1z_Airyscan Processing-34-part3-2/221020 NB SD AGO1b-568 AGO1d-488 DAPI PMC-1z_Airyscan Processing-34-part3-2_z34_ChA-T1.tif]

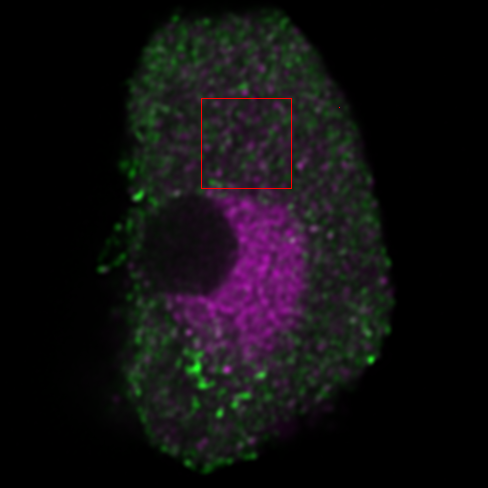

Supplement: Supplementary file 16 — Source_Data_zip [file 41467_2023_38881_MOESM16_ESM.zip › Source_Data_files_RK/Fig_5/Fig5_i-l_221020 NB SD AGO1b-568 AGO1d-488 DAPI PMC-1z_Airyscan Processing-34-part3-2/221020 NB SD AGO1b-568 AGO1d-488 DAPI PMC-1z_Airyscan Processing.tif]

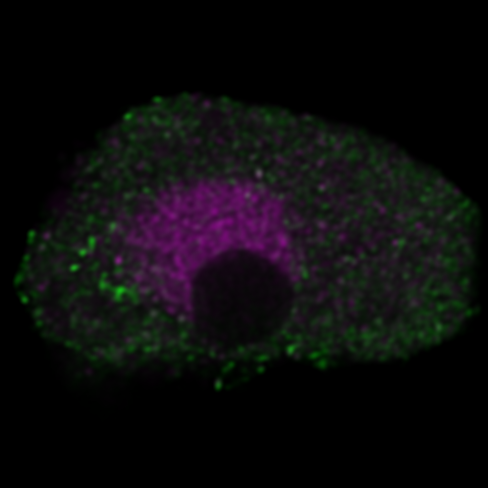

Supplement: Supplementary file 16 — Source_Data_zip [file 41467_2023_38881_MOESM16_ESM.zip › Source_Data_files_RK/Fig_5/Fig5_i-l_221020 NB SD AGO1b-568 AGO1d-488 DAPI PMC-1z_Airyscan Processing-34-part3-2/221020 NB SD AGO1b-568 AGO1d-488 DAPI PMC-1z_Airyscan Processing-34-part3-2_z34.tif]

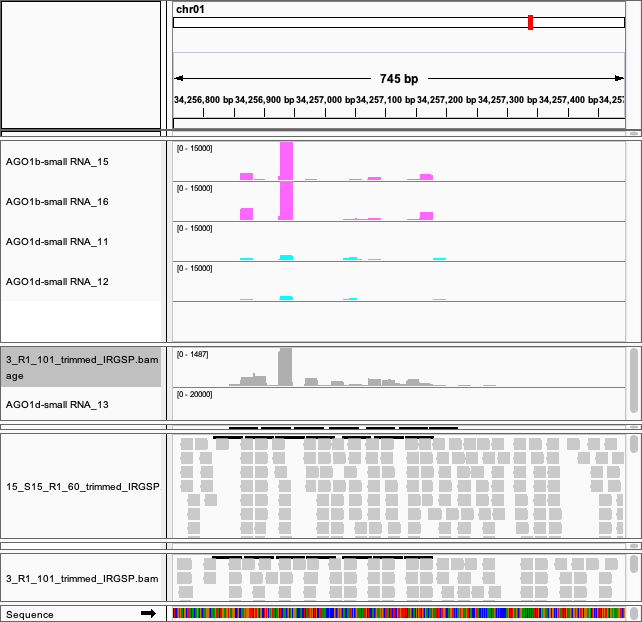

Supplement: Supplementary file 16 — Source_Data_zip [file 41467_2023_38881_MOESM16_ESM.zip › Source_Data_files_RK/Fig_3/Fig3_d_1-32_new_igv_snapshot.png]

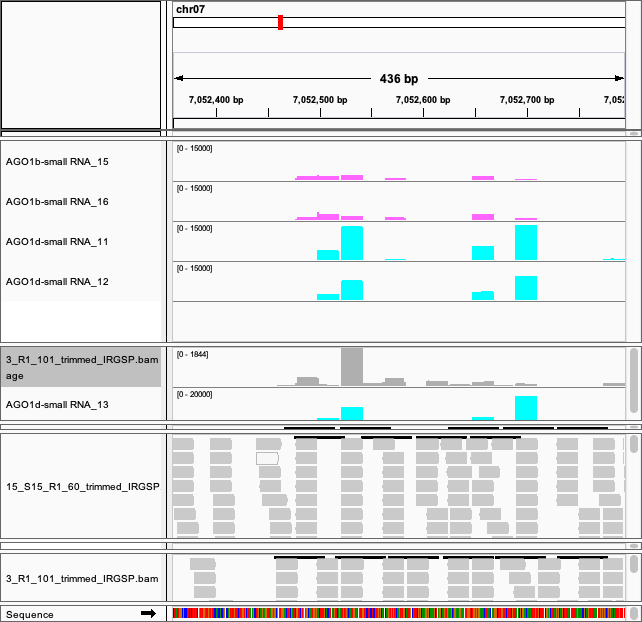

Supplement: Supplementary file 16 — Source_Data_zip [file 41467_2023_38881_MOESM16_ESM.zip › Source_Data_files_RK/Fig_3/Fig3_e_7-55_igv_snapshot.png]

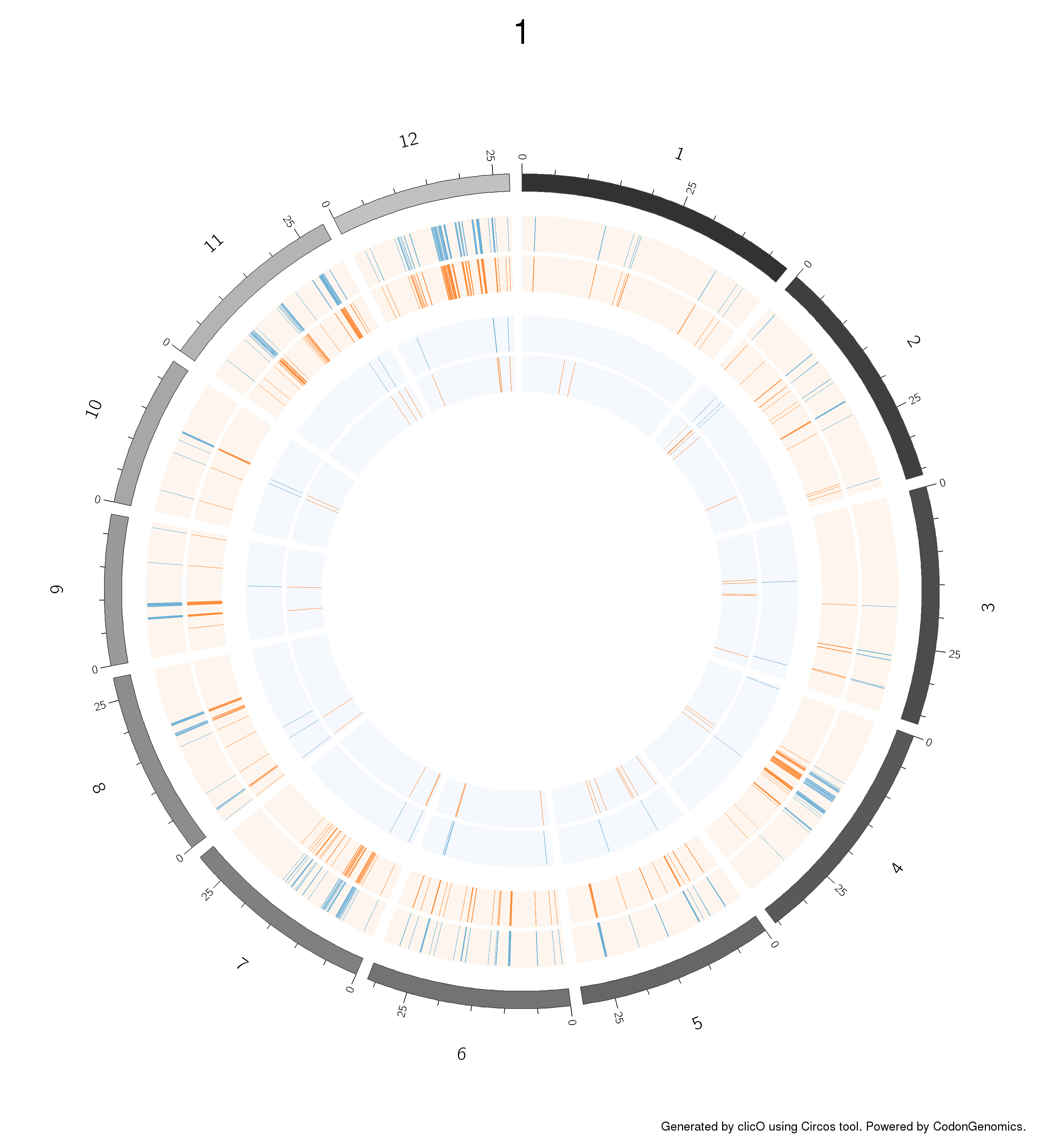

Supplement: Supplementary file 16 — Source_Data_zip [file 41467_2023_38881_MOESM16_ESM.zip › Source_Data_files_RK/Fig_3/Fig3_a_output_20210219162502.png]

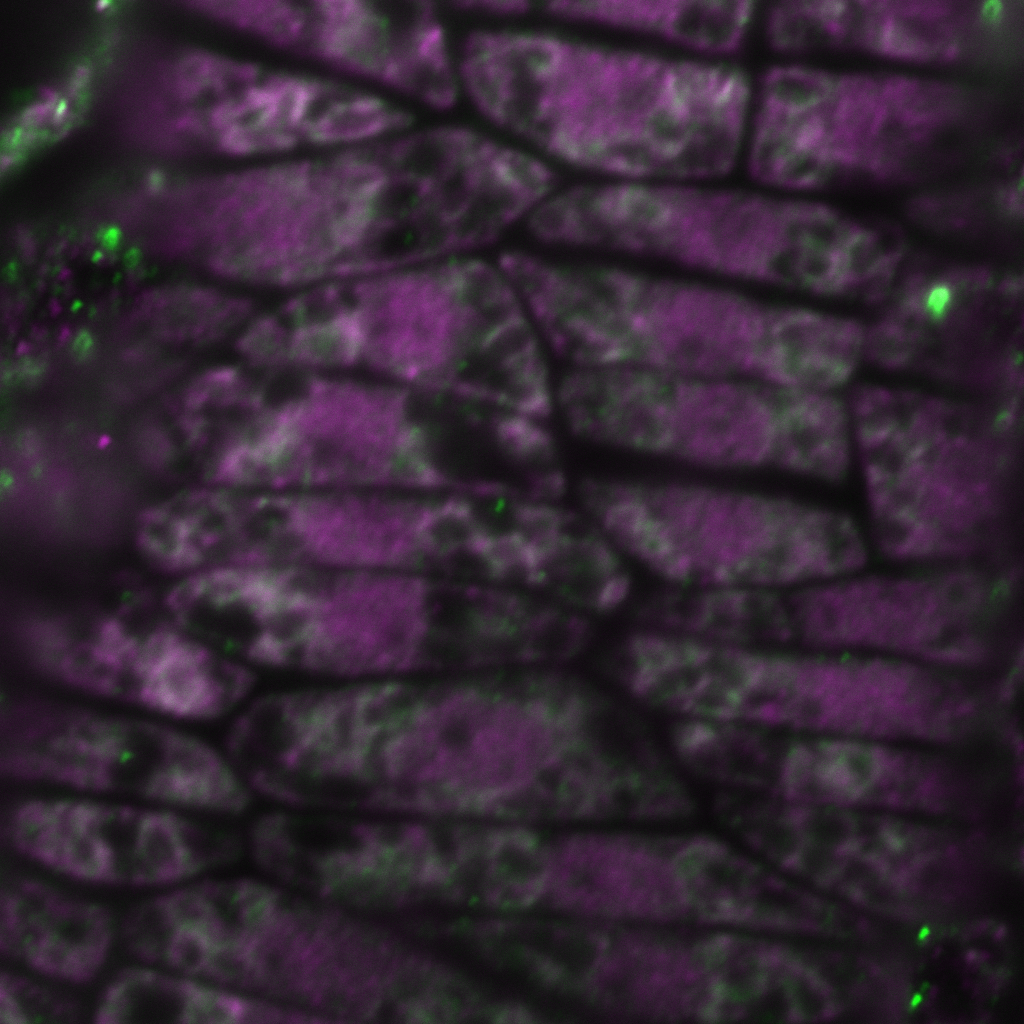

Supplement: Supplementary file 16 — Source_Data_zip [file 41467_2023_38881_MOESM16_ESM.zip › Source_Data_files_RK/Fig_4/e-h/210205 AGO1b-568 AGO1d-488 SD 0.5mm-15En-63(2)_c1-3.tif]

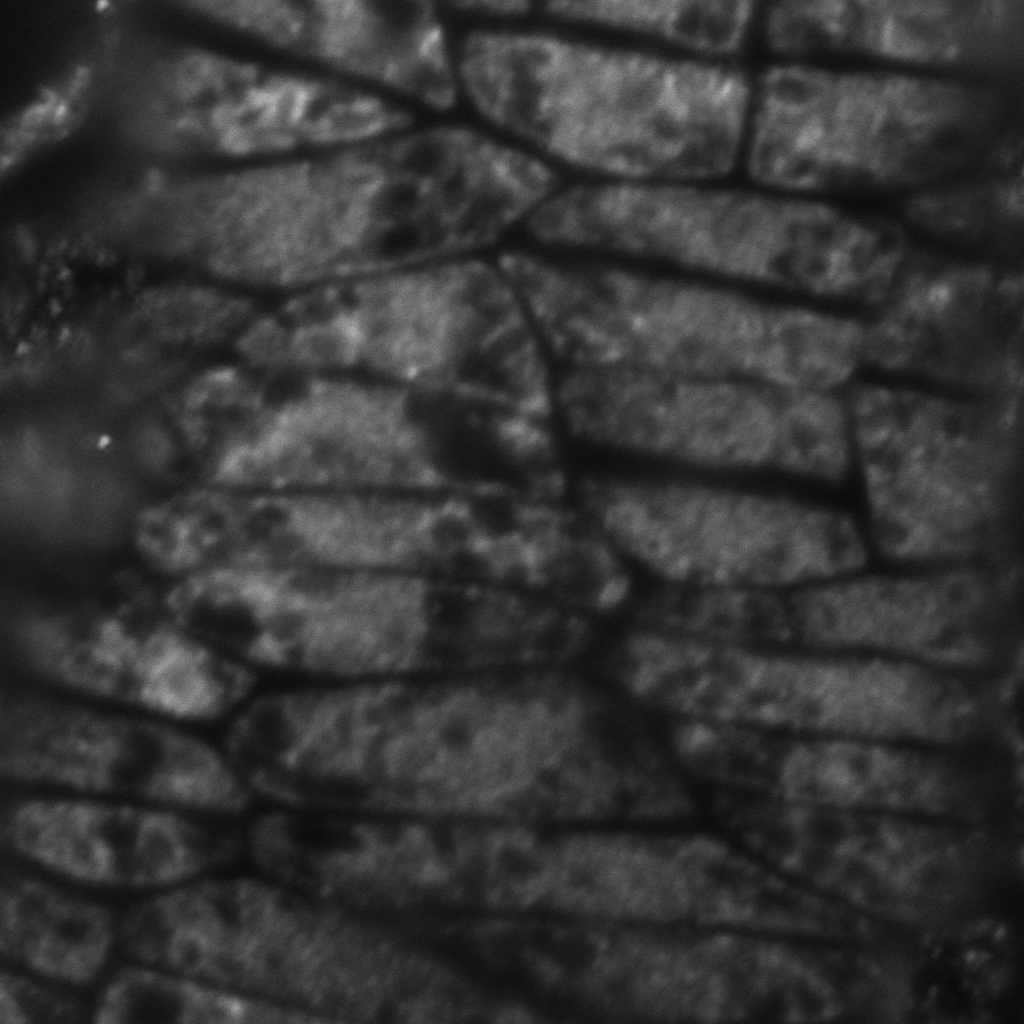

Supplement: Supplementary file 16 — Source_Data_zip [file 41467_2023_38881_MOESM16_ESM.zip › Source_Data_files_RK/Fig_4/e-h/210205 AGO1b-568 AGO1d-488 SD 0.5mm-15En-63(W)_c3.tif]

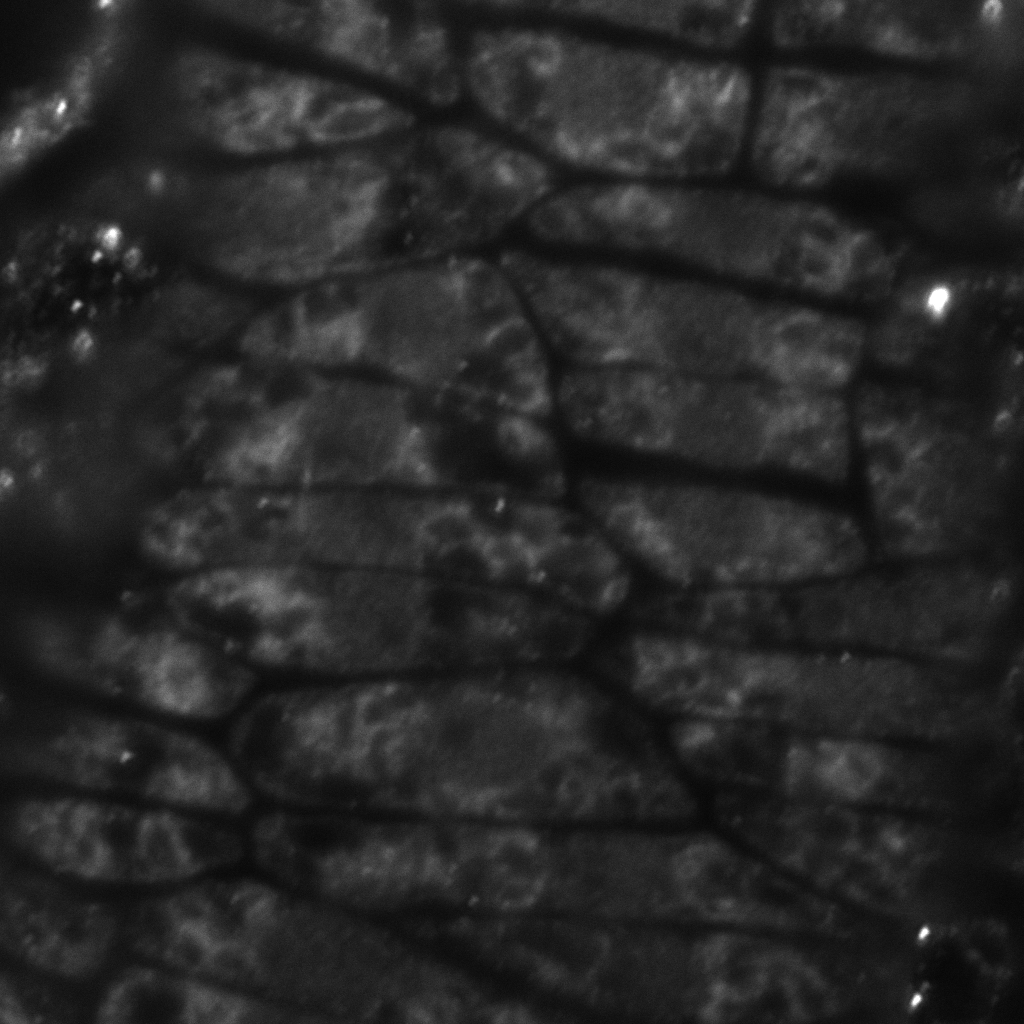

Supplement: Supplementary file 16 — Source_Data_zip [file 41467_2023_38881_MOESM16_ESM.zip › Source_Data_files_RK/Fig_4/e-h/210205 AGO1b-568 AGO1d-488 SD 0.5mm-15En-63(W)_c2.tif]

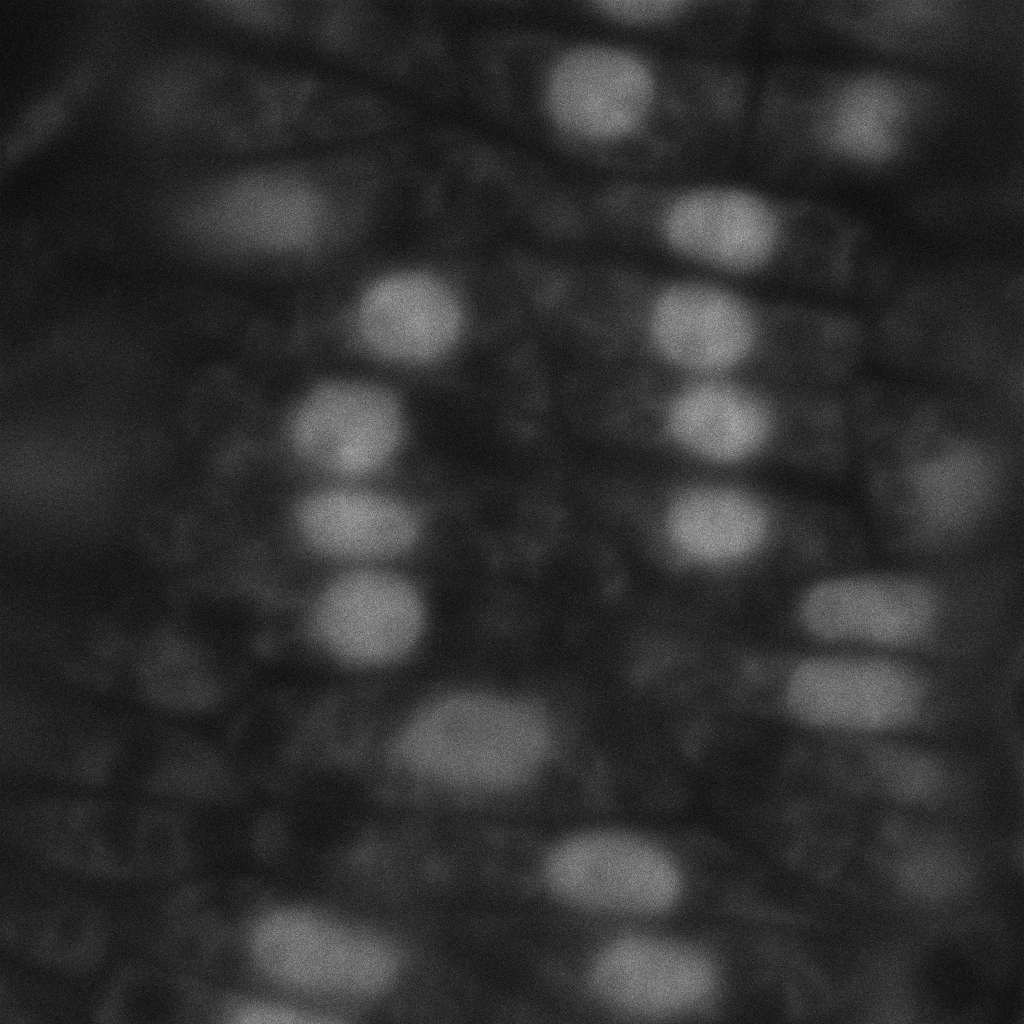

Supplement: Supplementary file 16 — Source_Data_zip [file 41467_2023_38881_MOESM16_ESM.zip › Source_Data_files_RK/Fig_4/e-h/210205 AGO1b-568 AGO1d-488 SD 0.5mm-15En-63(W)_c1.tif]

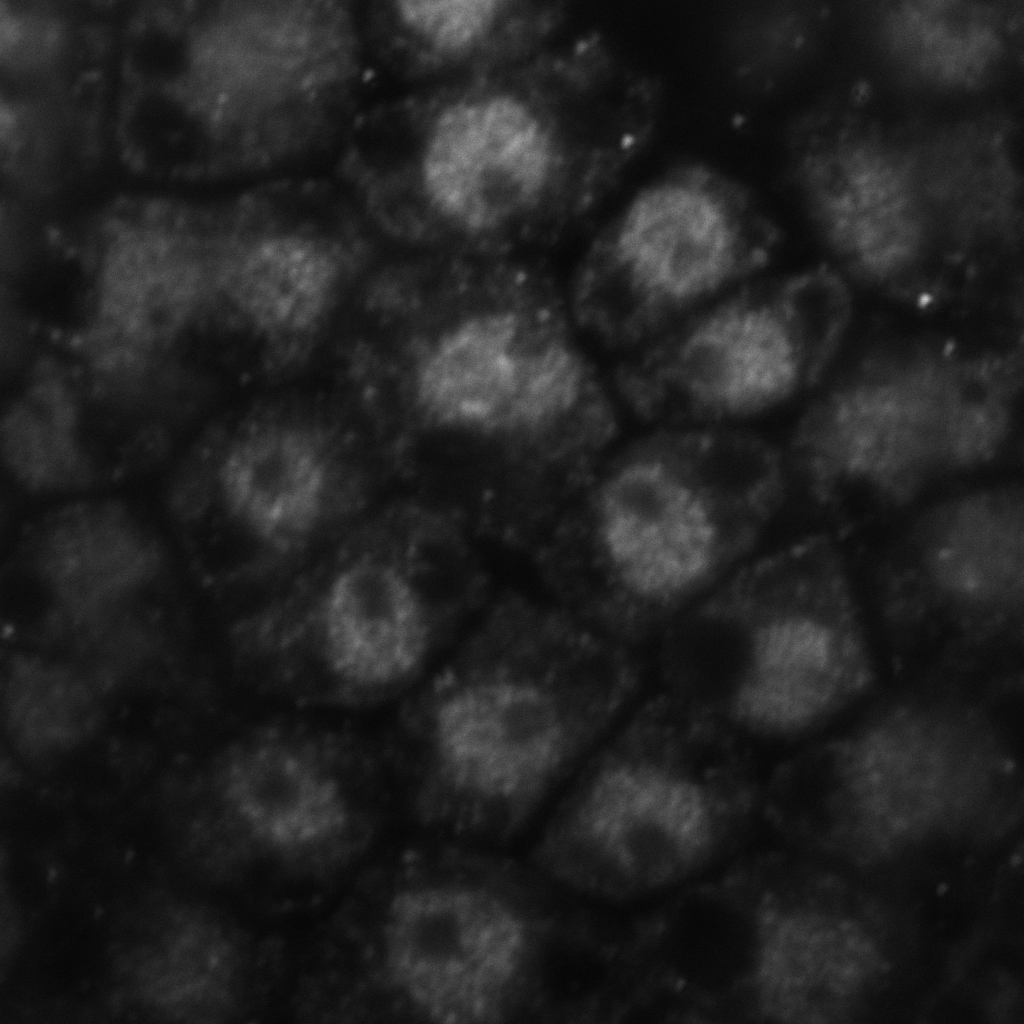

Supplement: Supplementary file 16 — Source_Data_zip [file 41467_2023_38881_MOESM16_ESM.zip › Source_Data_files_RK/Fig_4/m-p/210205 AGO1b-568 AGO1d-488 SD 0.5mm-18Ta-63(W)_c3.tif]

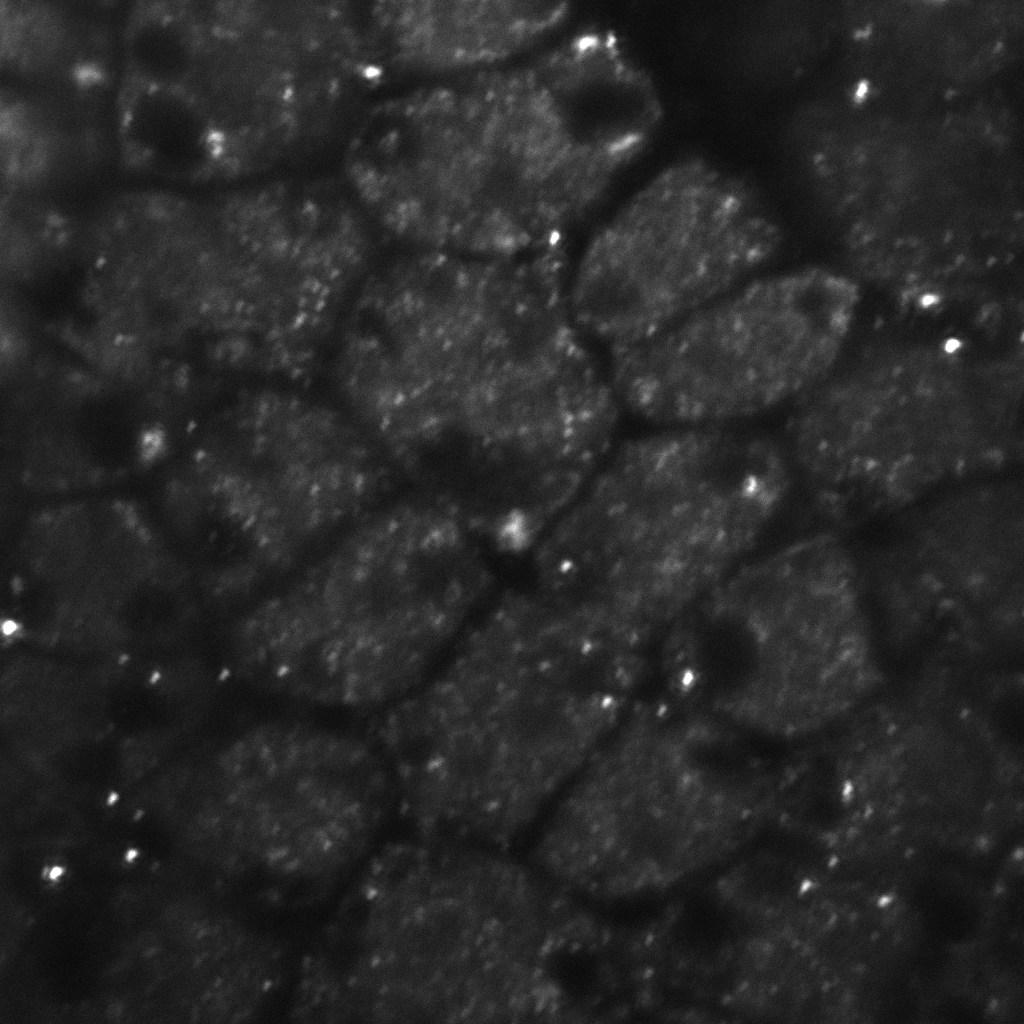

Supplement: Supplementary file 16 — Source_Data_zip [file 41467_2023_38881_MOESM16_ESM.zip › Source_Data_files_RK/Fig_4/m-p/210205 AGO1b-568 AGO1d-488 SD 0.5mm-18Ta-63(W)_c2.tif]

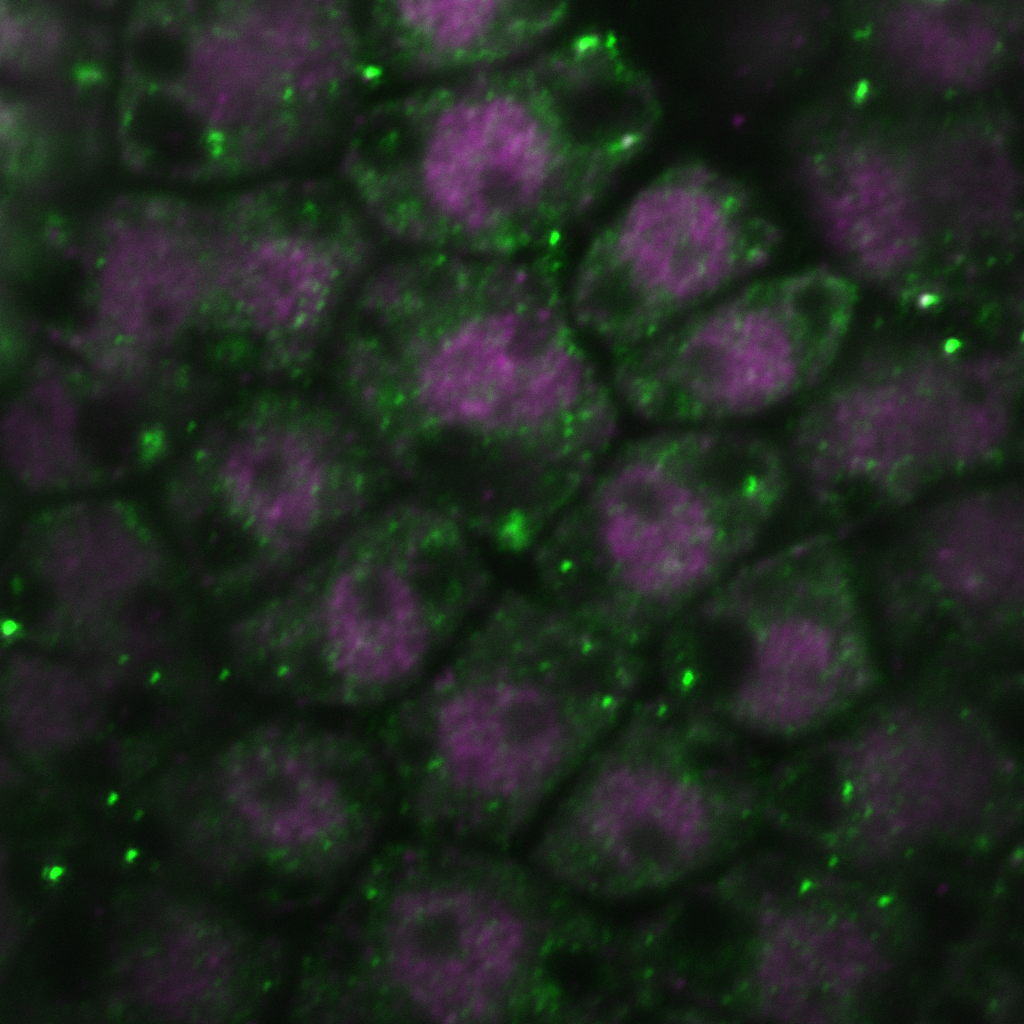

Supplement: Supplementary file 16 — Source_Data_zip [file 41467_2023_38881_MOESM16_ESM.zip › Source_Data_files_RK/Fig_4/m-p/210205 AGO1b-568 AGO1d-488 SD 0.5mm-18Ta-63(2)_c1-3.tif]

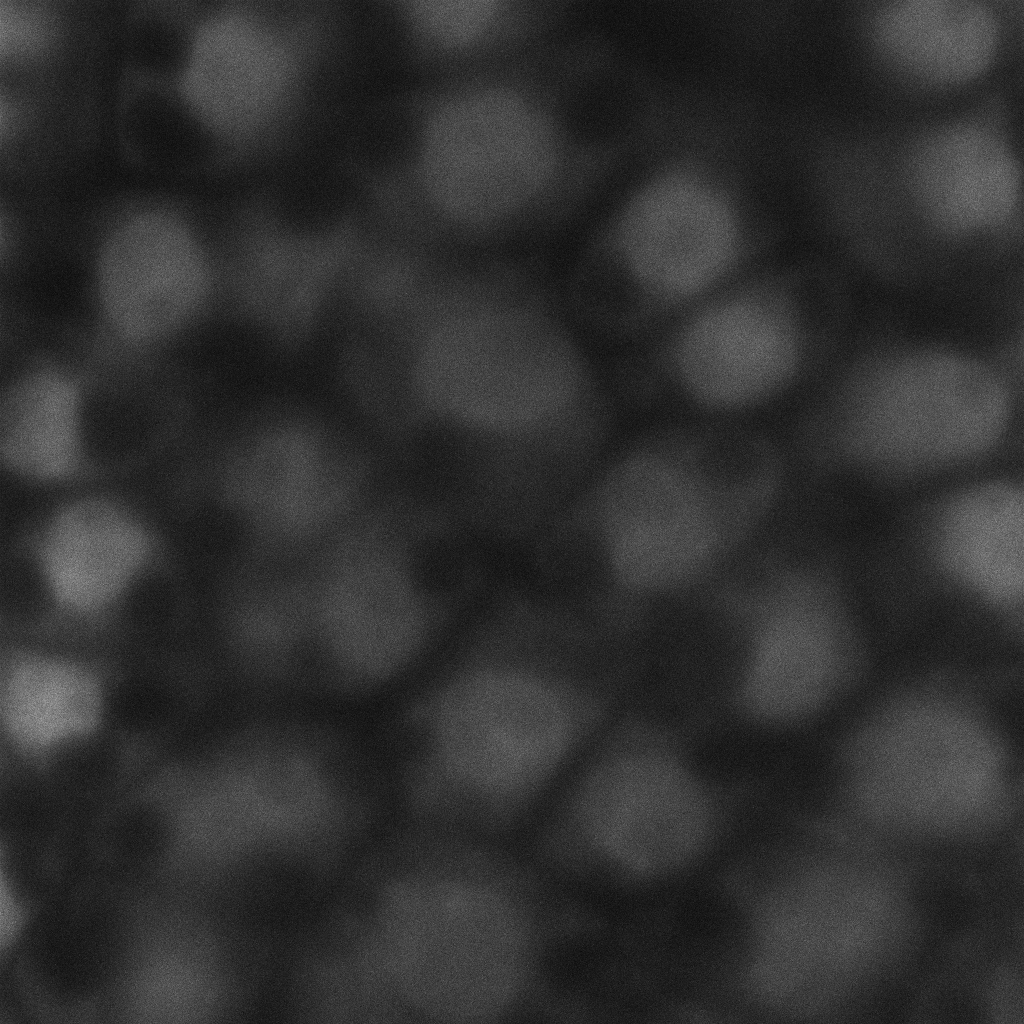

Supplement: Supplementary file 16 — Source_Data_zip [file 41467_2023_38881_MOESM16_ESM.zip › Source_Data_files_RK/Fig_4/m-p/210205 AGO1b-568 AGO1d-488 SD 0.5mm-18Ta-63(W)_c1.tif]

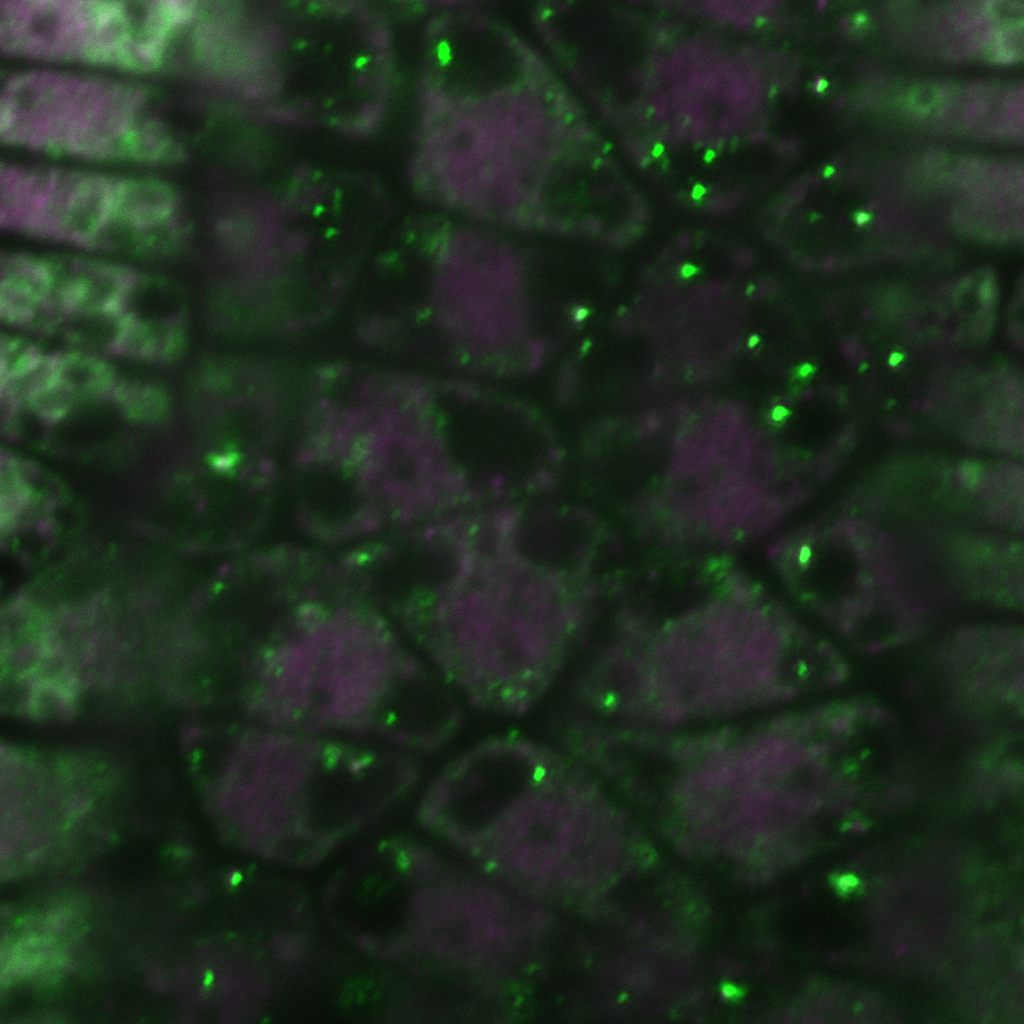

Supplement: Supplementary file 16 — Source_Data_zip [file 41467_2023_38881_MOESM16_ESM.zip › Source_Data_files_RK/Fig_4/i-l/210205 AGO1b-568 AGO1d-488 SD 0.5mm-17ML-63(2)_c1-3.tif]

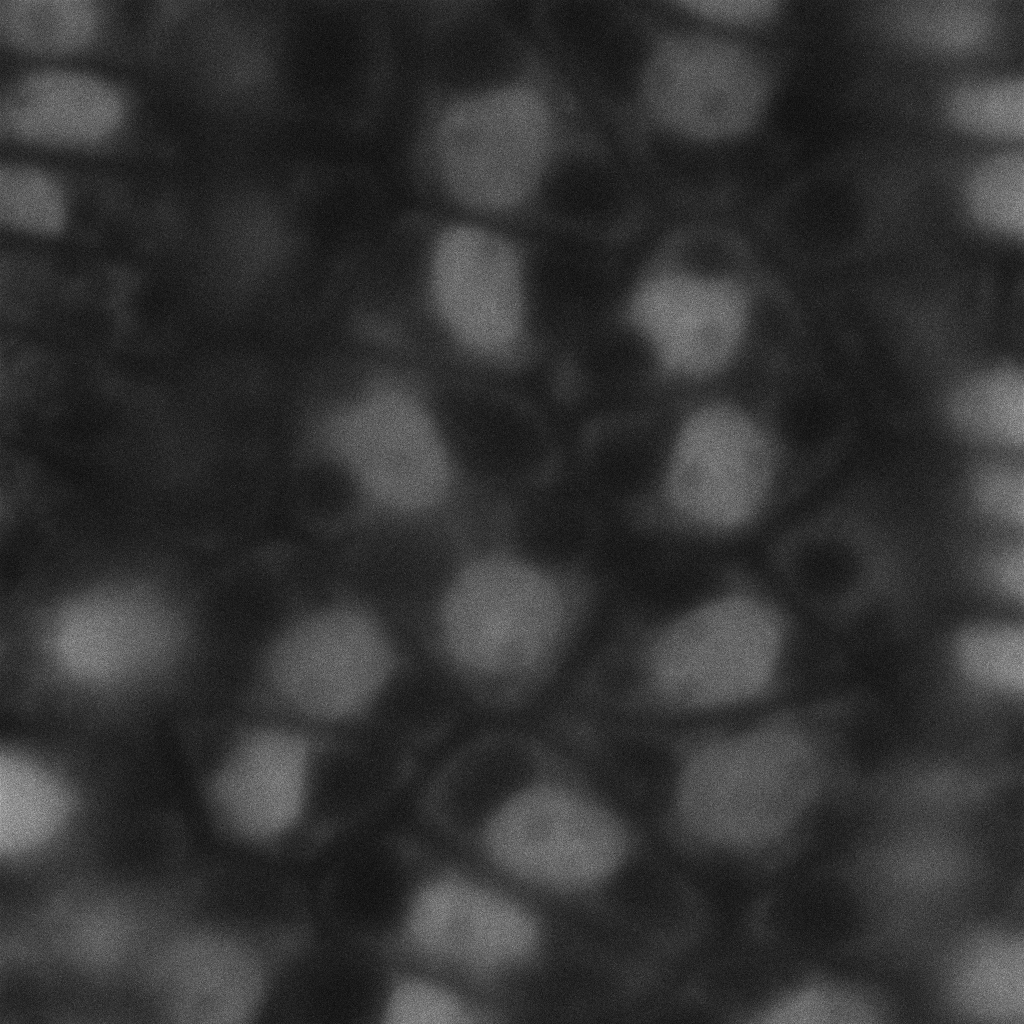

Supplement: Supplementary file 16 — Source_Data_zip [file 41467_2023_38881_MOESM16_ESM.zip › Source_Data_files_RK/Fig_4/i-l/210205 AGO1b-568 AGO1d-488 SD 0.5mm-17ML-63(W)_c1.tif]

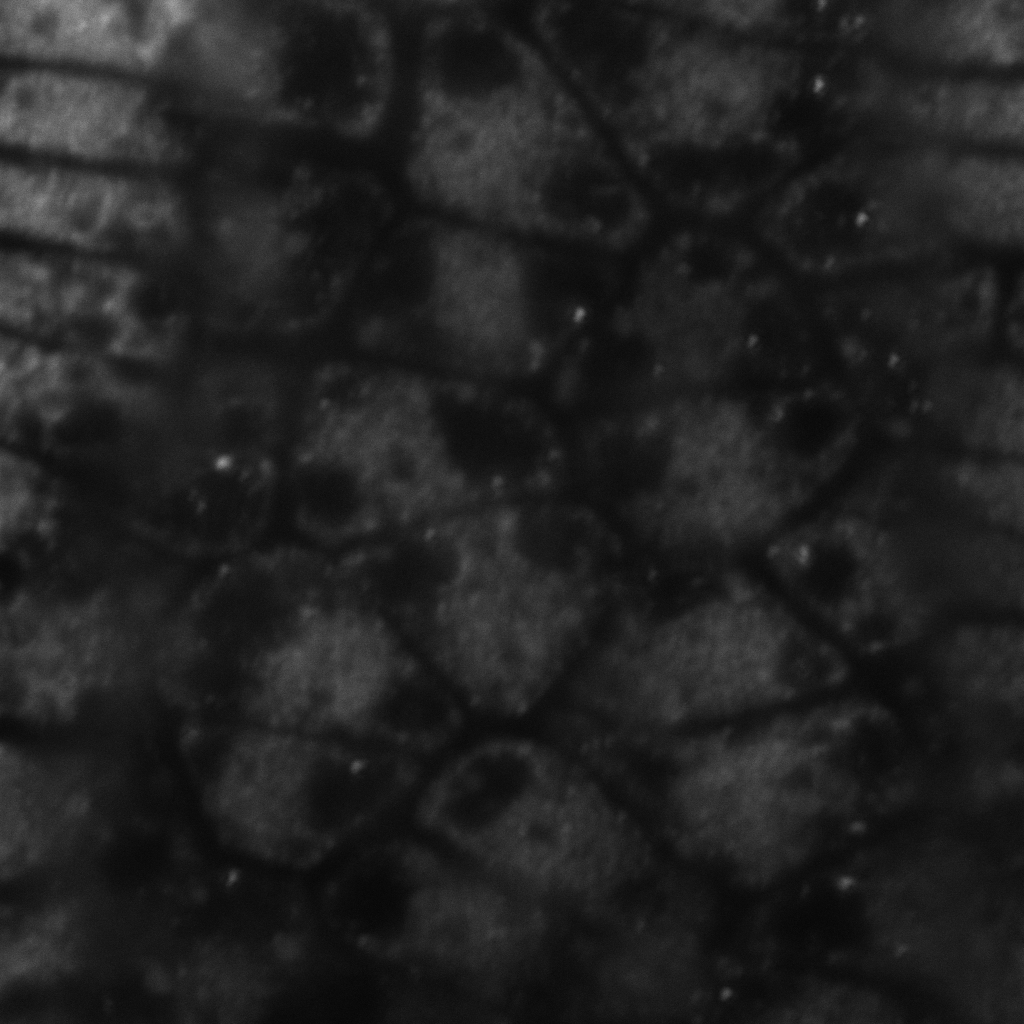

Supplement: Supplementary file 16 — Source_Data_zip [file 41467_2023_38881_MOESM16_ESM.zip › Source_Data_files_RK/Fig_4/i-l/210205 AGO1b-568 AGO1d-488 SD 0.5mm-17ML-63(W)_c3.tif]

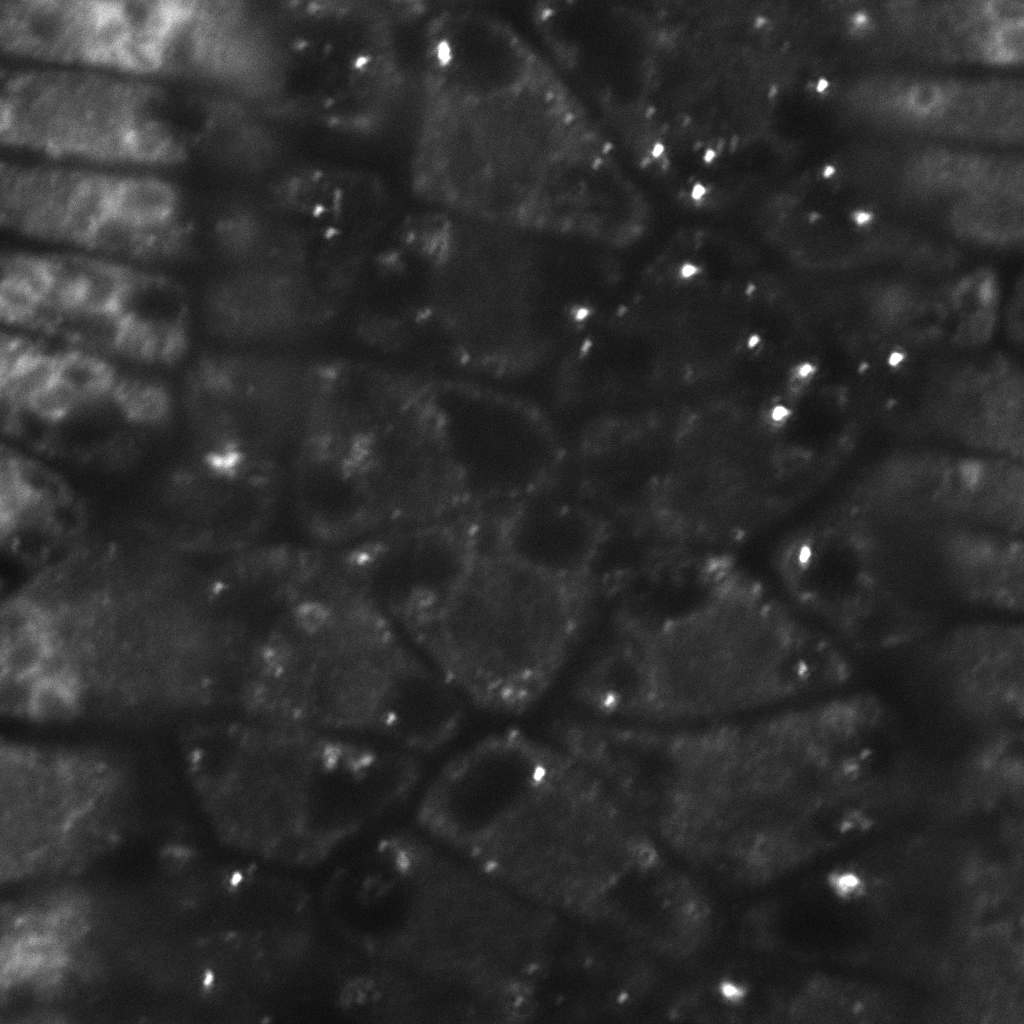

Supplement: Supplementary file 16 — Source_Data_zip [file 41467_2023_38881_MOESM16_ESM.zip › Source_Data_files_RK/Fig_4/i-l/210205 AGO1b-568 AGO1d-488 SD 0.5mm-17ML-63(W)_c2.tif]

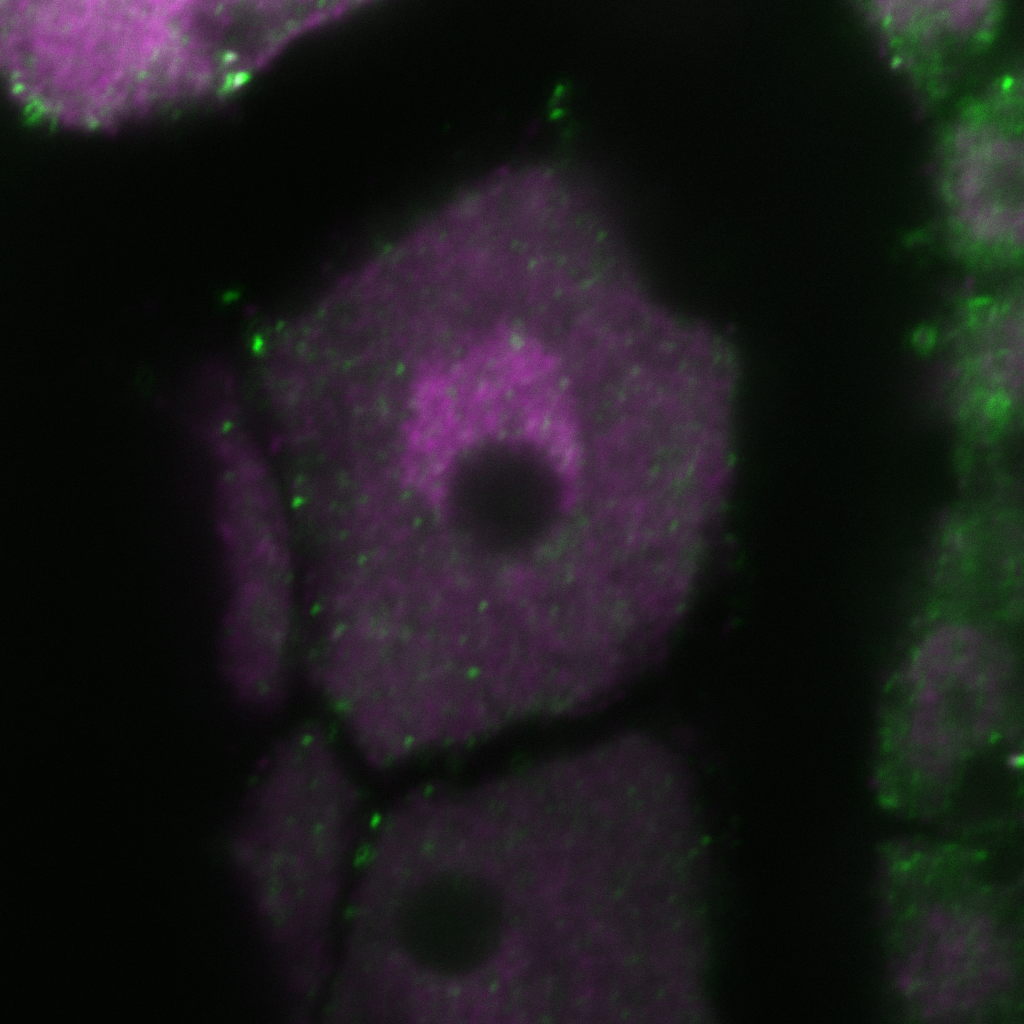

Supplement: Supplementary file 16 — Source_Data_zip [file 41467_2023_38881_MOESM16_ESM.zip › Source_Data_files_RK/Fig_4/q-t/210205 AGO1b-568 AGO1d-488 SD 0.5mm-19PMC-63(2)_c1-3.tif]

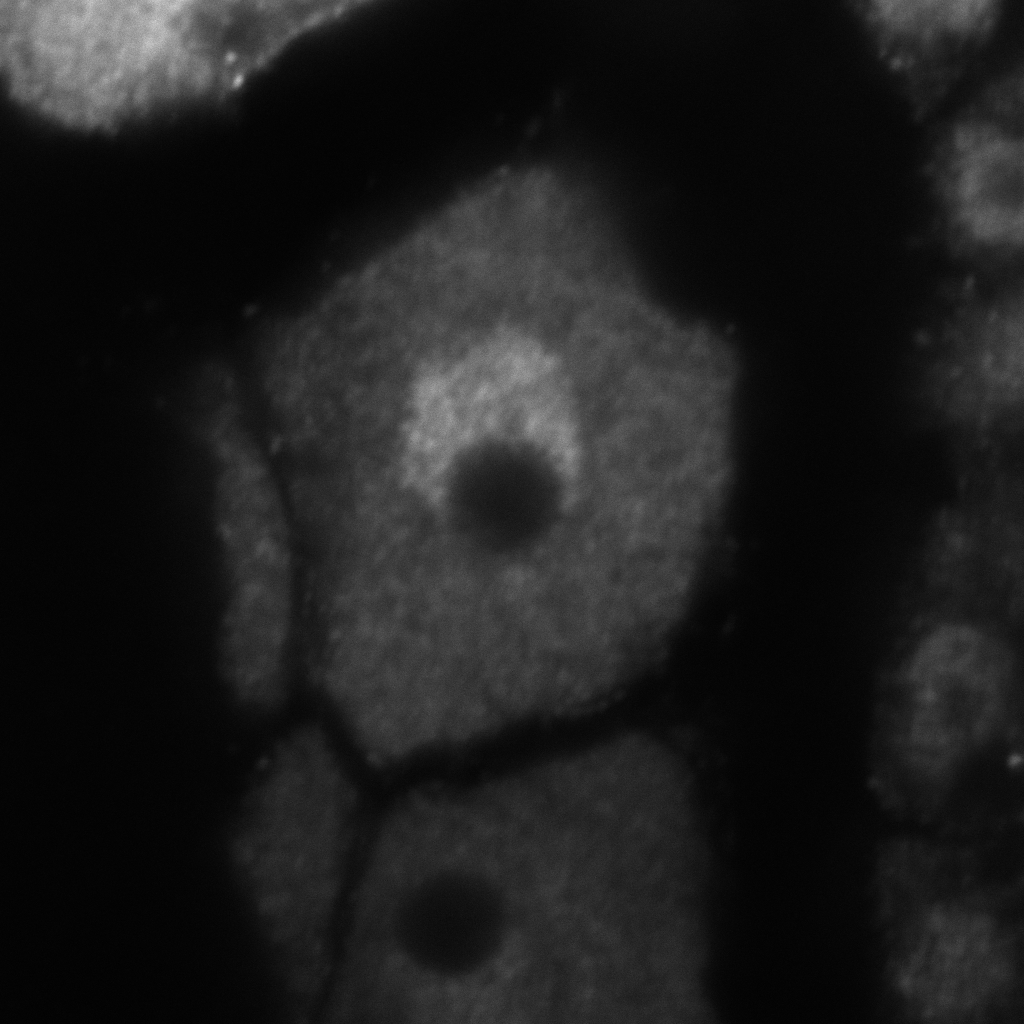

Supplement: Supplementary file 16 — Source_Data_zip [file 41467_2023_38881_MOESM16_ESM.zip › Source_Data_files_RK/Fig_4/q-t/210205 AGO1b-568 AGO1d-488 SD 0.5mm-19PMC-63(W)_c3.tif]

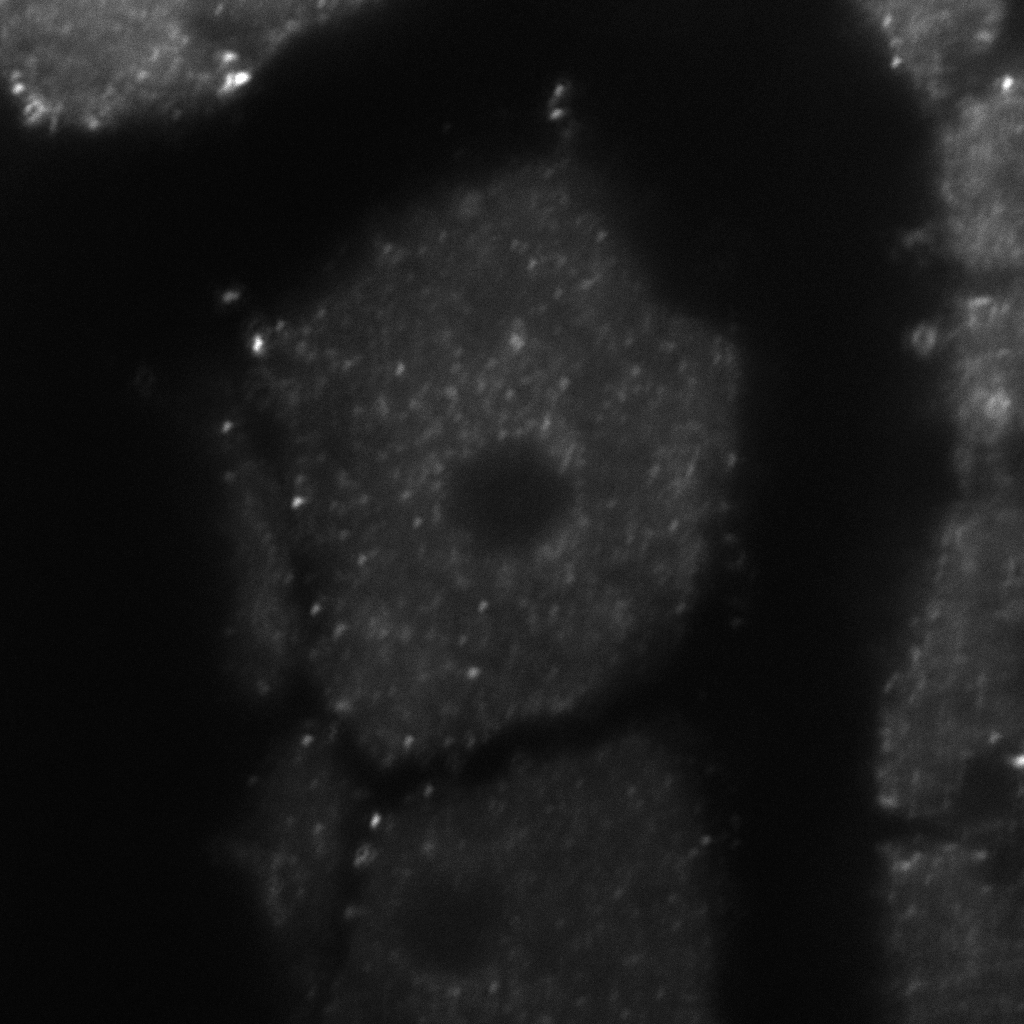

Supplement: Supplementary file 16 — Source_Data_zip [file 41467_2023_38881_MOESM16_ESM.zip › Source_Data_files_RK/Fig_4/q-t/210205 AGO1b-568 AGO1d-488 SD 0.5mm-19PMC-63(W)_c2.tif]

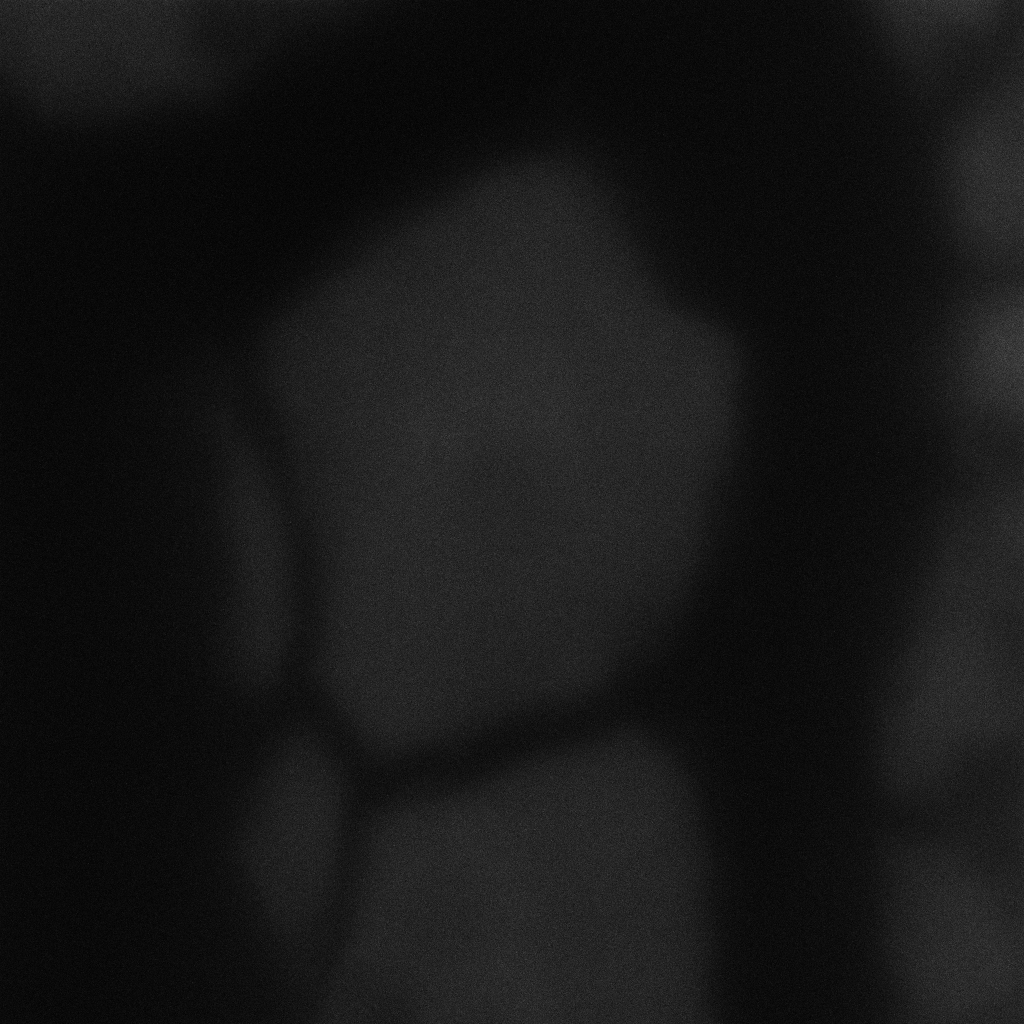

Supplement: Supplementary file 16 — Source_Data_zip [file 41467_2023_38881_MOESM16_ESM.zip › Source_Data_files_RK/Fig_4/q-t/210205 AGO1b-568 AGO1d-488 SD 0.5mm-19PMC-63(W)_c1.tif]

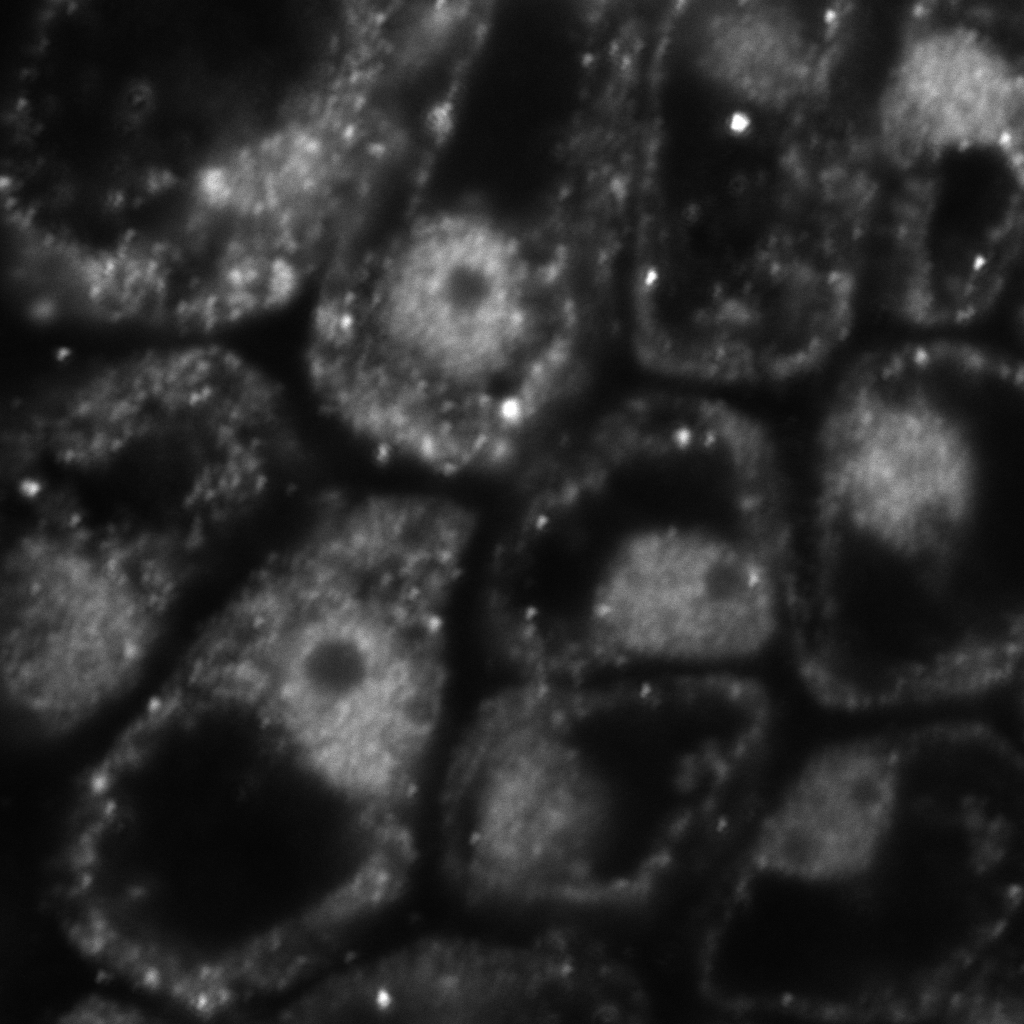

Supplement: Supplementary file 16 — Source_Data_zip [file 41467_2023_38881_MOESM16_ESM.zip › Source_Data_files_RK/Fig_4/a_d/210205 AGO1b-568 AGO1d-488 SD 0.5mm-14Ep-63(W)_c3.tif]

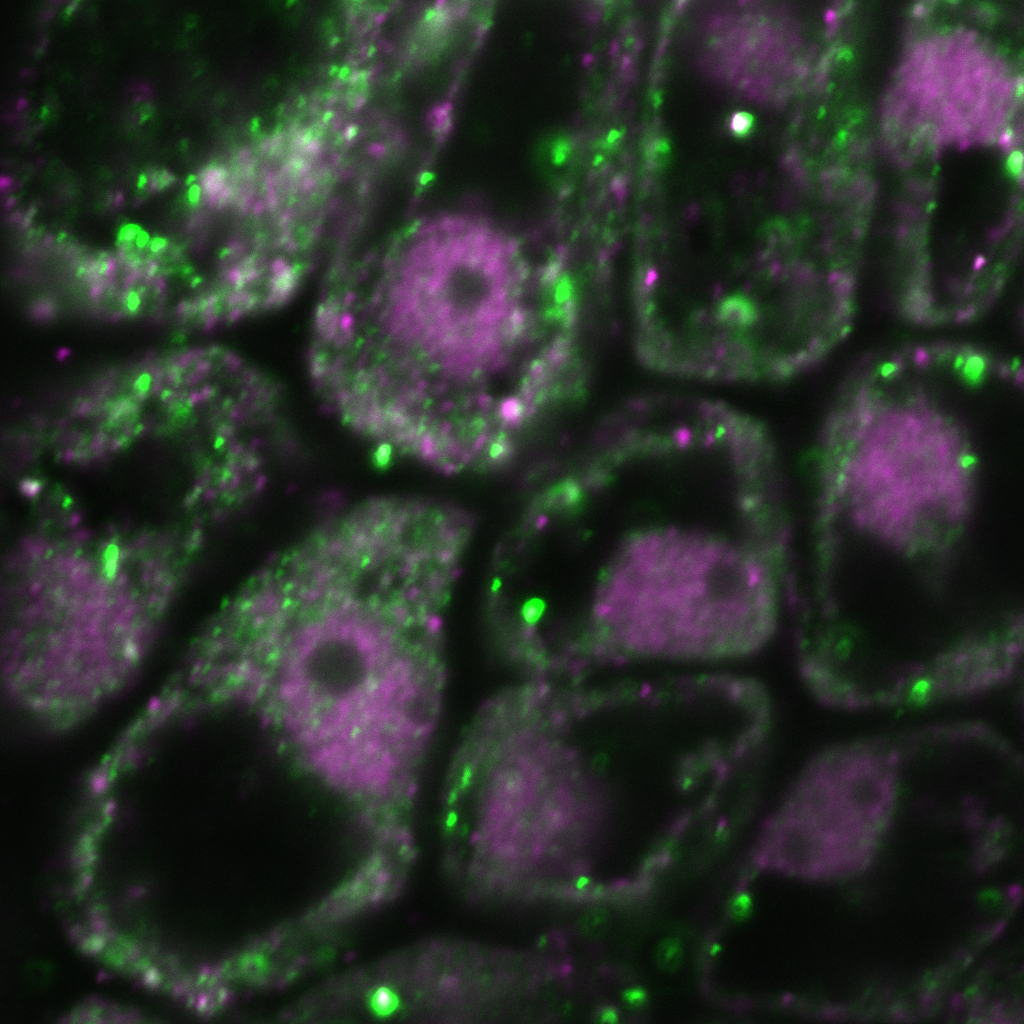

Supplement: Supplementary file 16 — Source_Data_zip [file 41467_2023_38881_MOESM16_ESM.zip › Source_Data_files_RK/Fig_4/a_d/210205 AGO1b-568 AGO1d-488 SD 0.5mm-14Ep-63(2)_c1-3.tif]

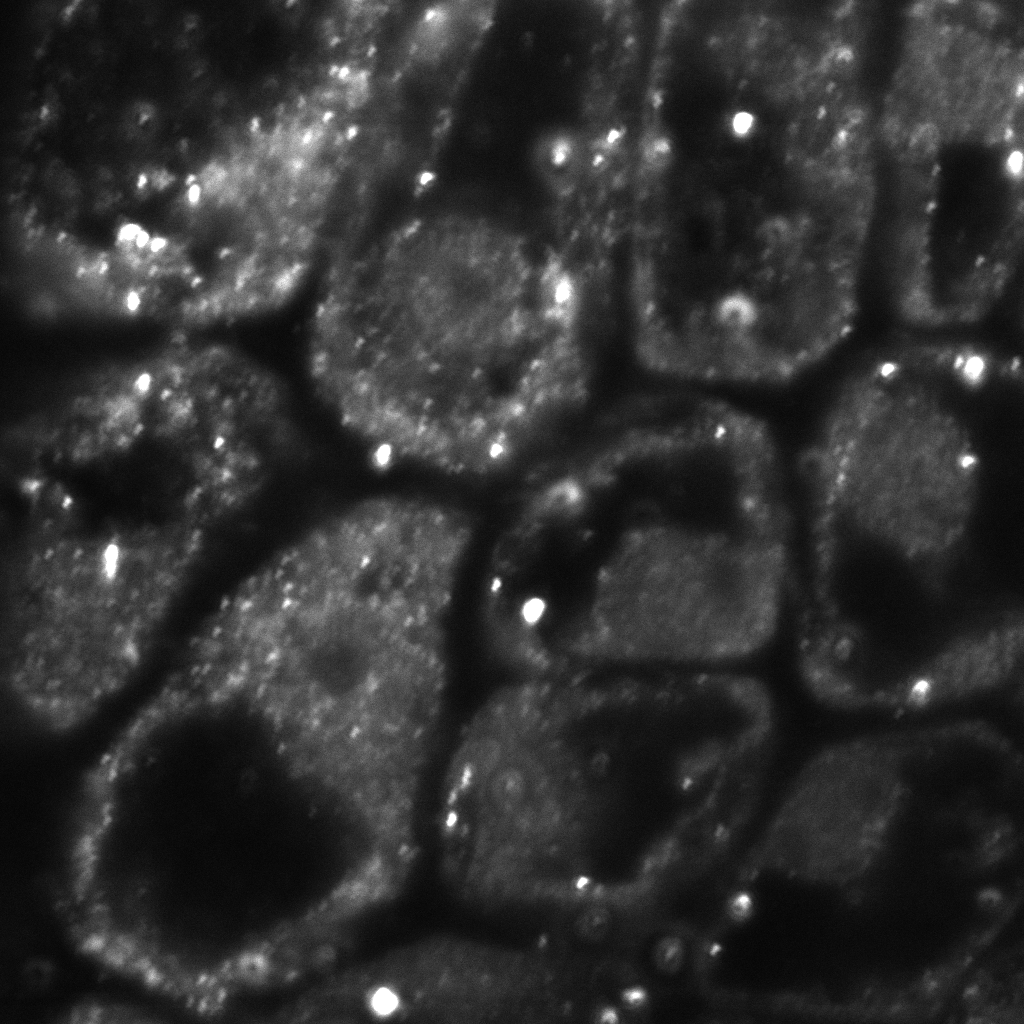

Supplement: Supplementary file 16 — Source_Data_zip [file 41467_2023_38881_MOESM16_ESM.zip › Source_Data_files_RK/Fig_4/a_d/210205 AGO1b-568 AGO1d-488 SD 0.5mm-14Ep-63(W)_c2.tif]

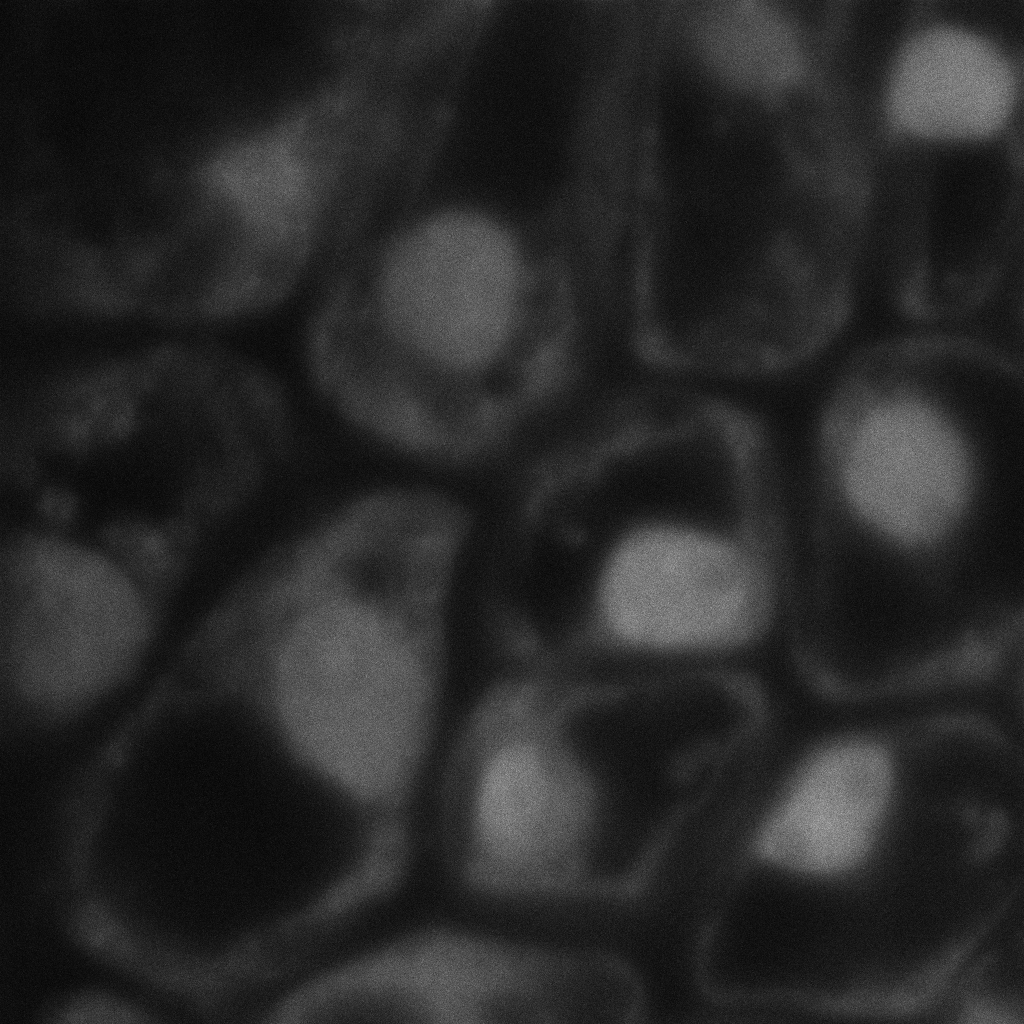

Supplement: Supplementary file 16 — Source_Data_zip [file 41467_2023_38881_MOESM16_ESM.zip › Source_Data_files_RK/Fig_4/a_d/210205 AGO1b-568 AGO1d-488 SD 0.5mm-14Ep-63(W)_c1.tif]

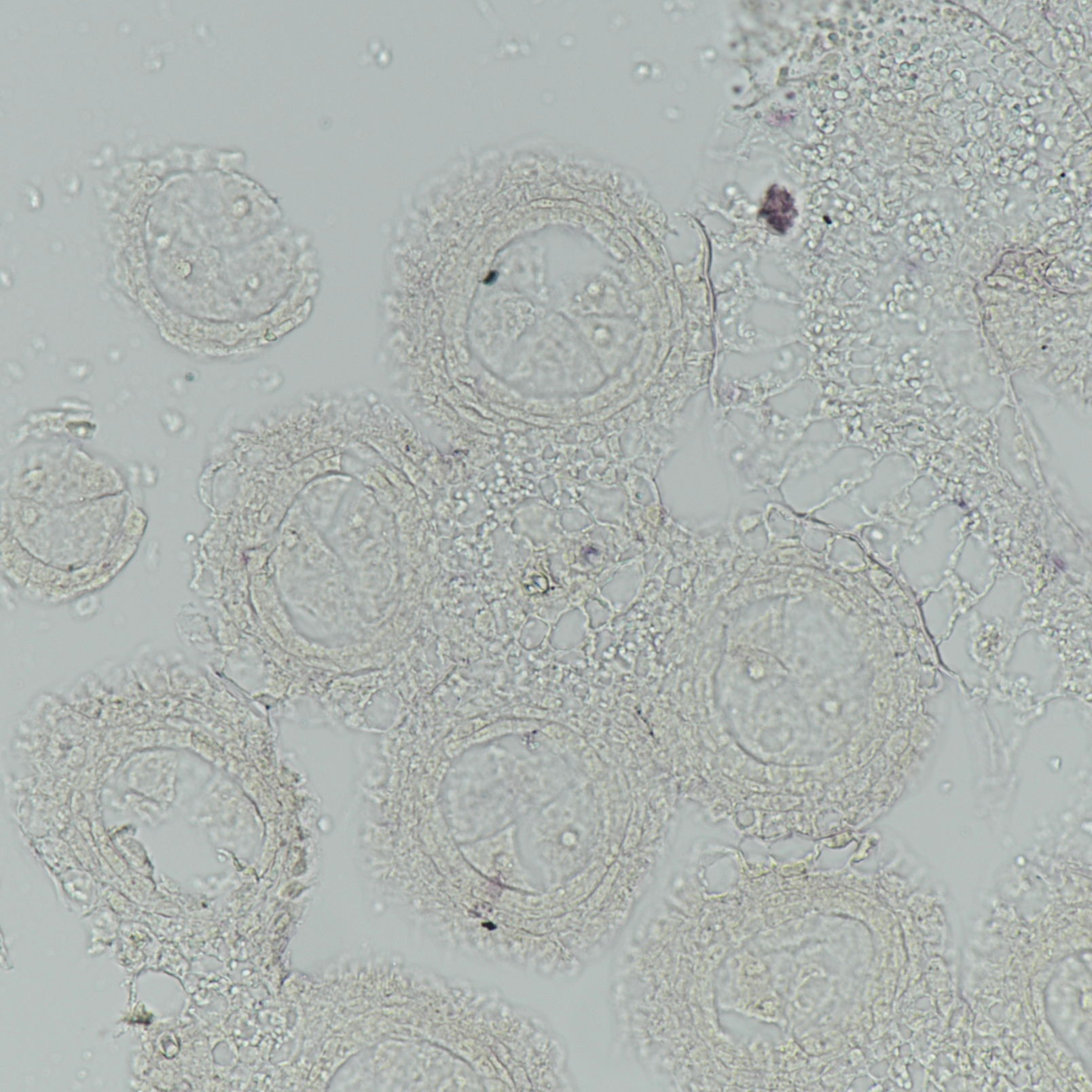

Supplement: Supplementary file 16 — Source_Data_zip [file 41467_2023_38881_MOESM16_ESM.zip › Source_Data_files_RK/SupFig_7/AGO1dN.tif]

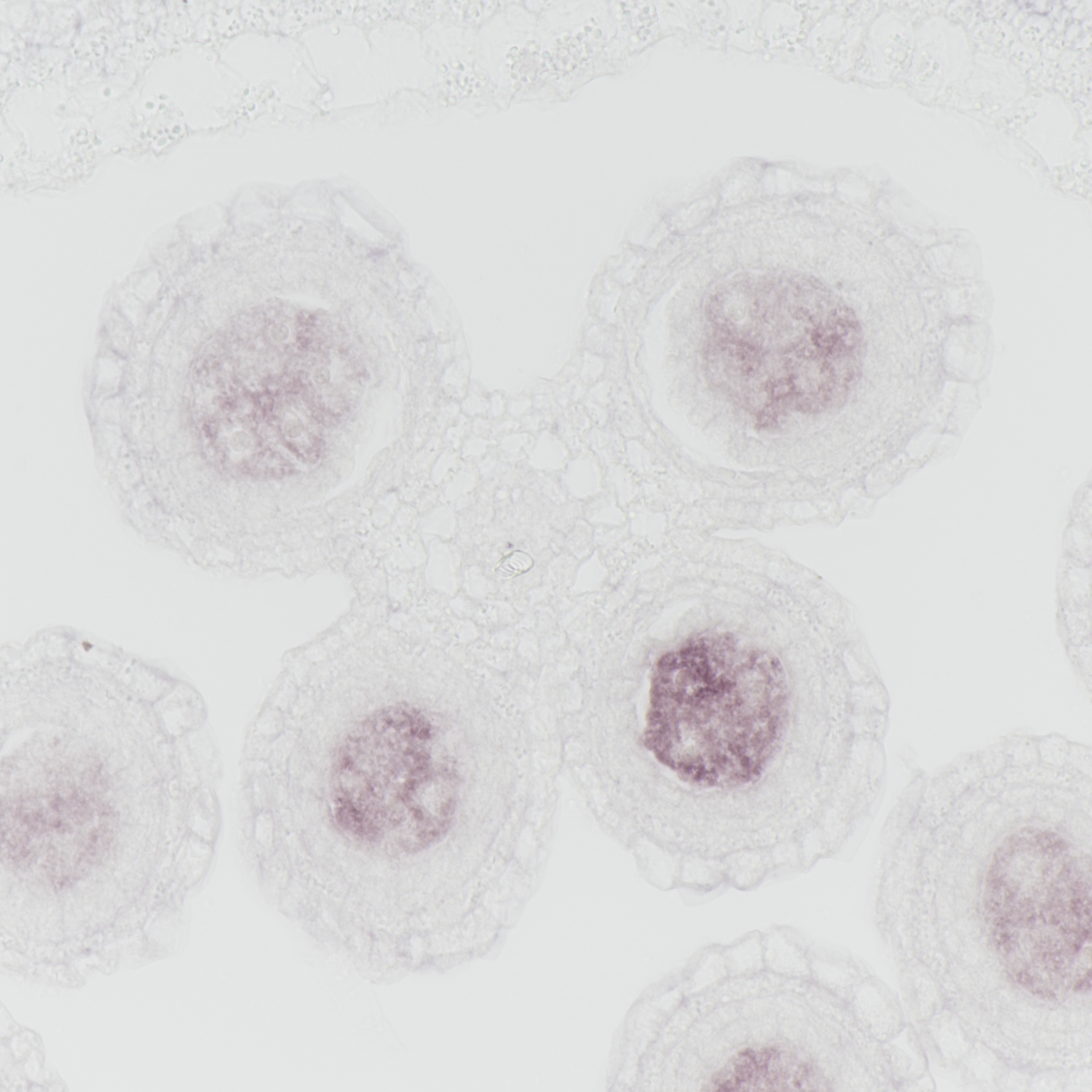

Supplement: Supplementary file 16 — Source_Data_zip [file 41467_2023_38881_MOESM16_ESM.zip › Source_Data_files_RK/SupFig_7/MEL1P.tif]

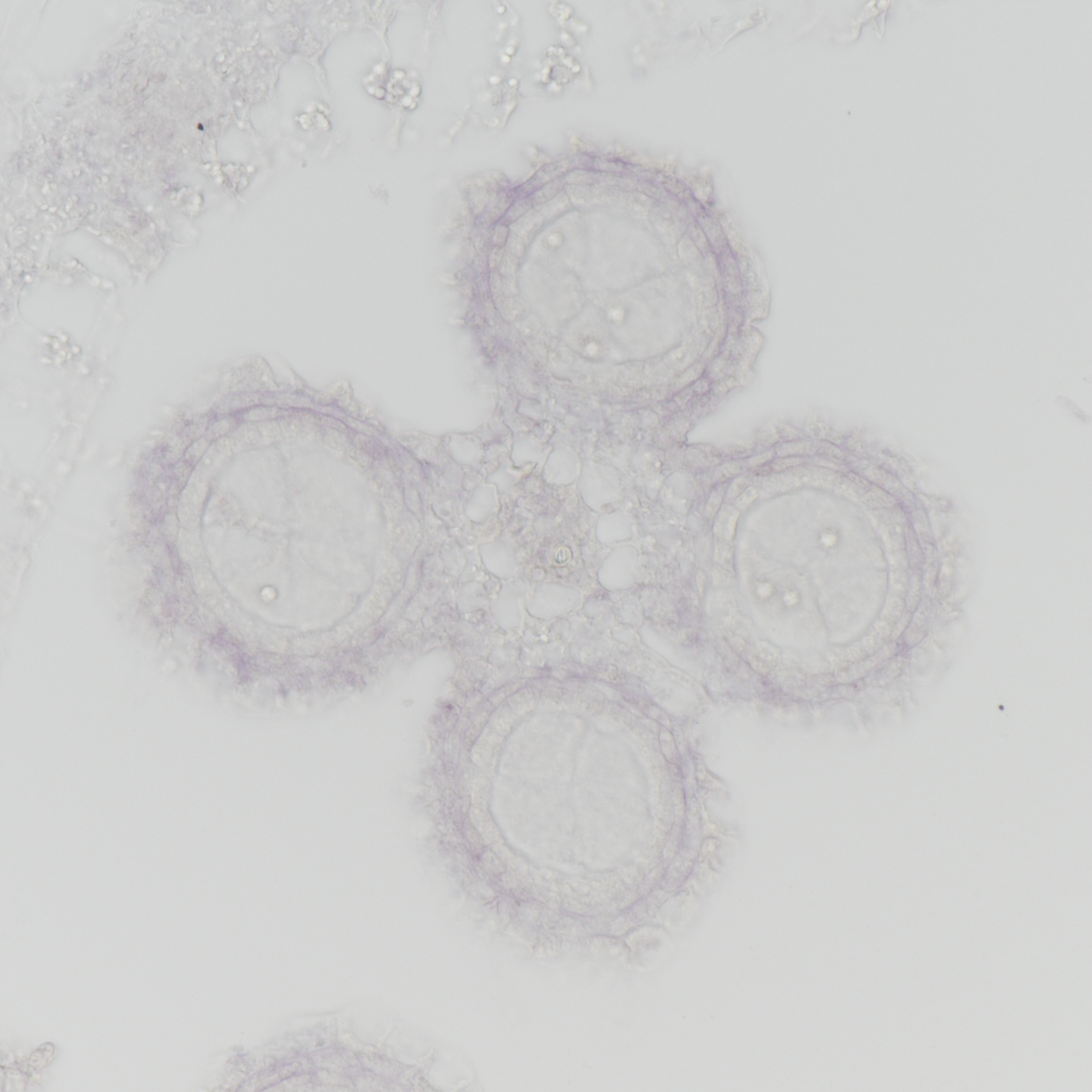

Supplement: Supplementary file 16 — Source_Data_zip [file 41467_2023_38881_MOESM16_ESM.zip › Source_Data_files_RK/SupFig_7/AGO1bP.tif]

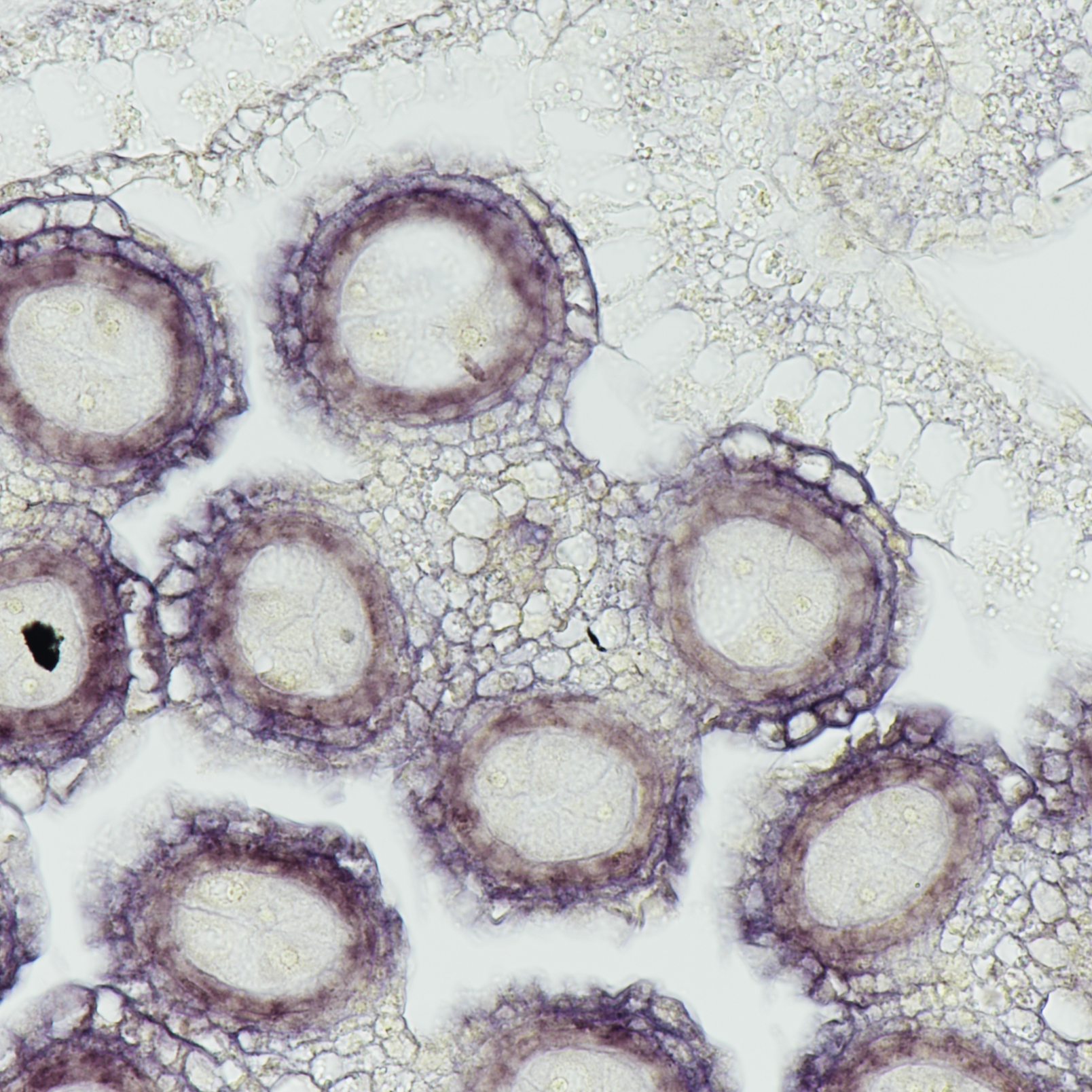

Supplement: Supplementary file 16 — Source_Data_zip [file 41467_2023_38881_MOESM16_ESM.zip › Source_Data_files_RK/SupFig_7/AGO1dP2.tif]

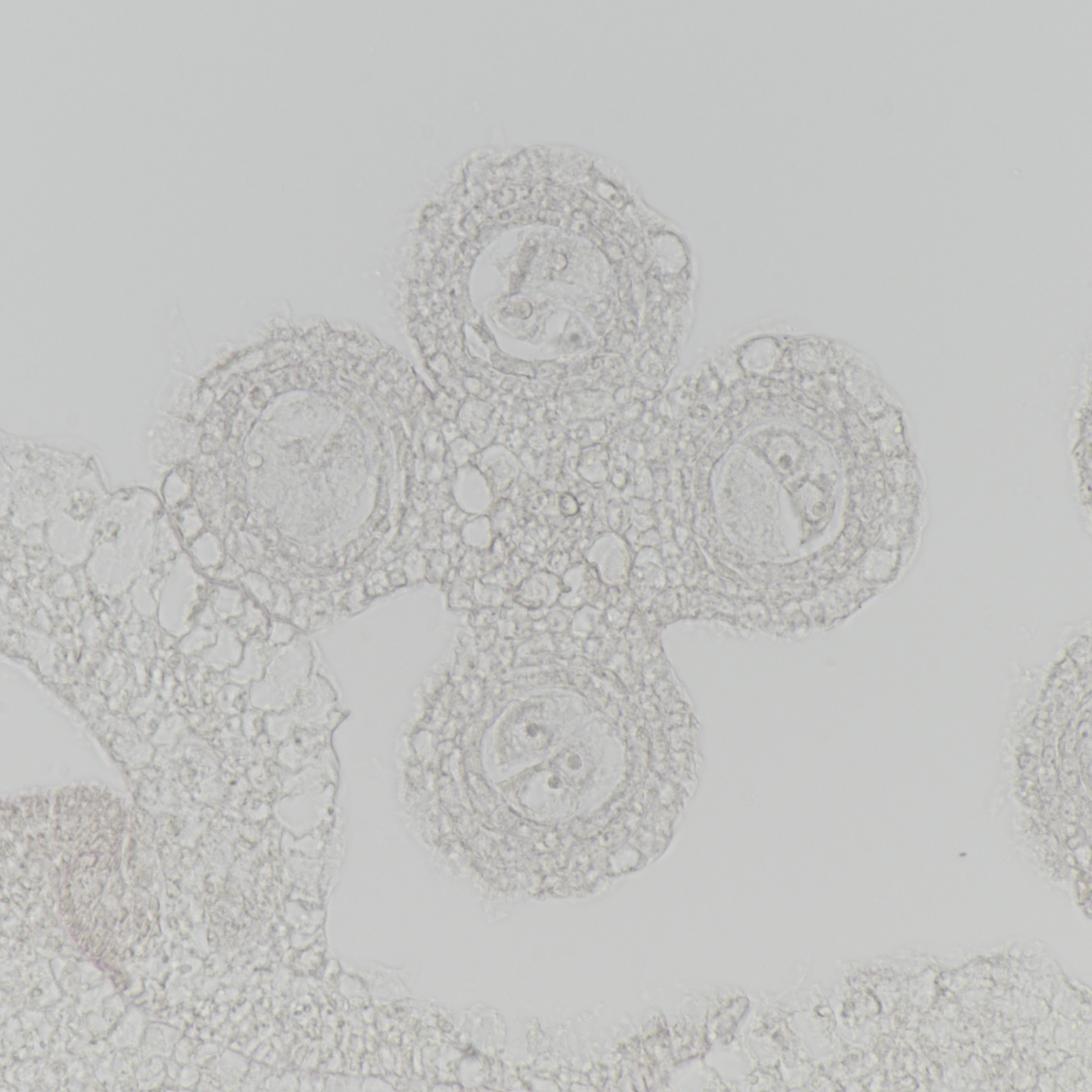

Supplement: Supplementary file 16 — Source_Data_zip [file 41467_2023_38881_MOESM16_ESM.zip › Source_Data_files_RK/SupFig_7/AGO1bN2.tif]

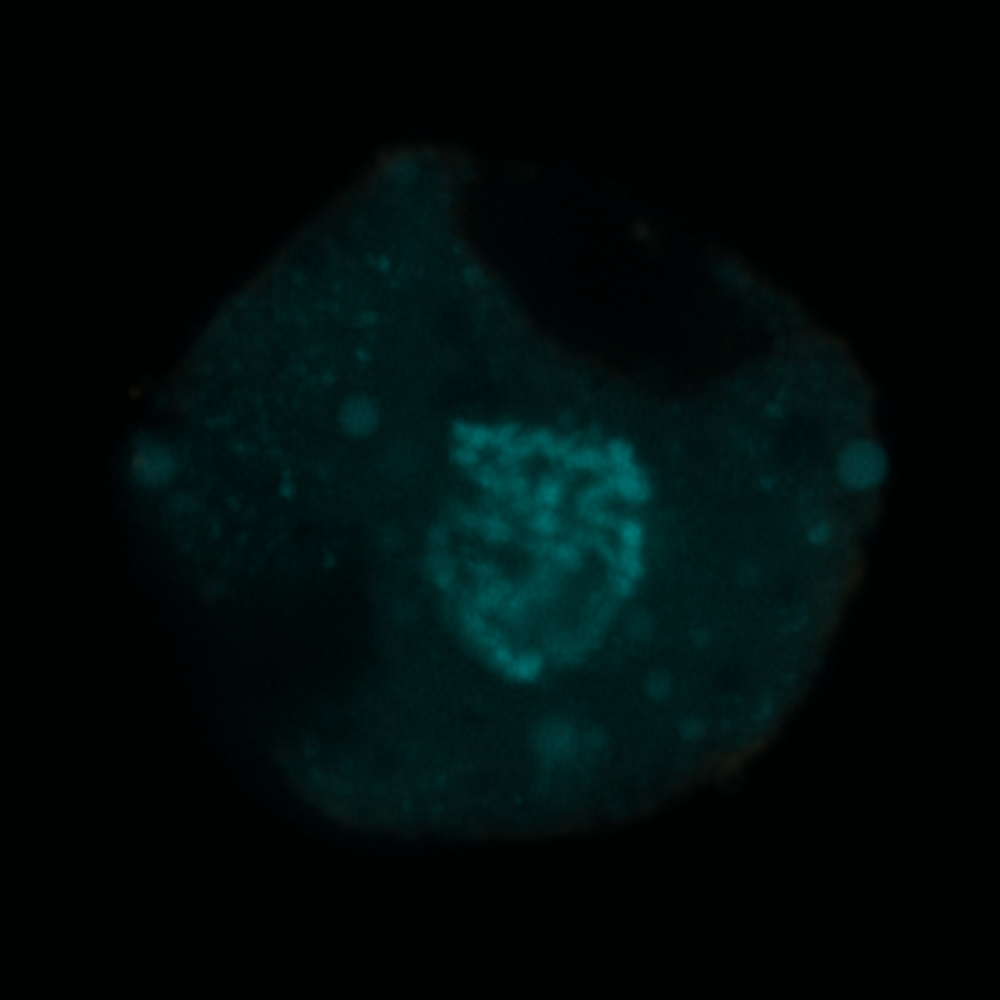

Supplement: Supplementary file 16 — Source_Data_zip [file 41467_2023_38881_MOESM16_ESM.zip › Source_Data_files_RK/SupFig_6/221031 NB SD 0.5A negative control PMC-2_Airyscan Processing_SupFig6a_e/221031 NB SD 0.5A negative control PMC-2_Airyscan Processing.tif]

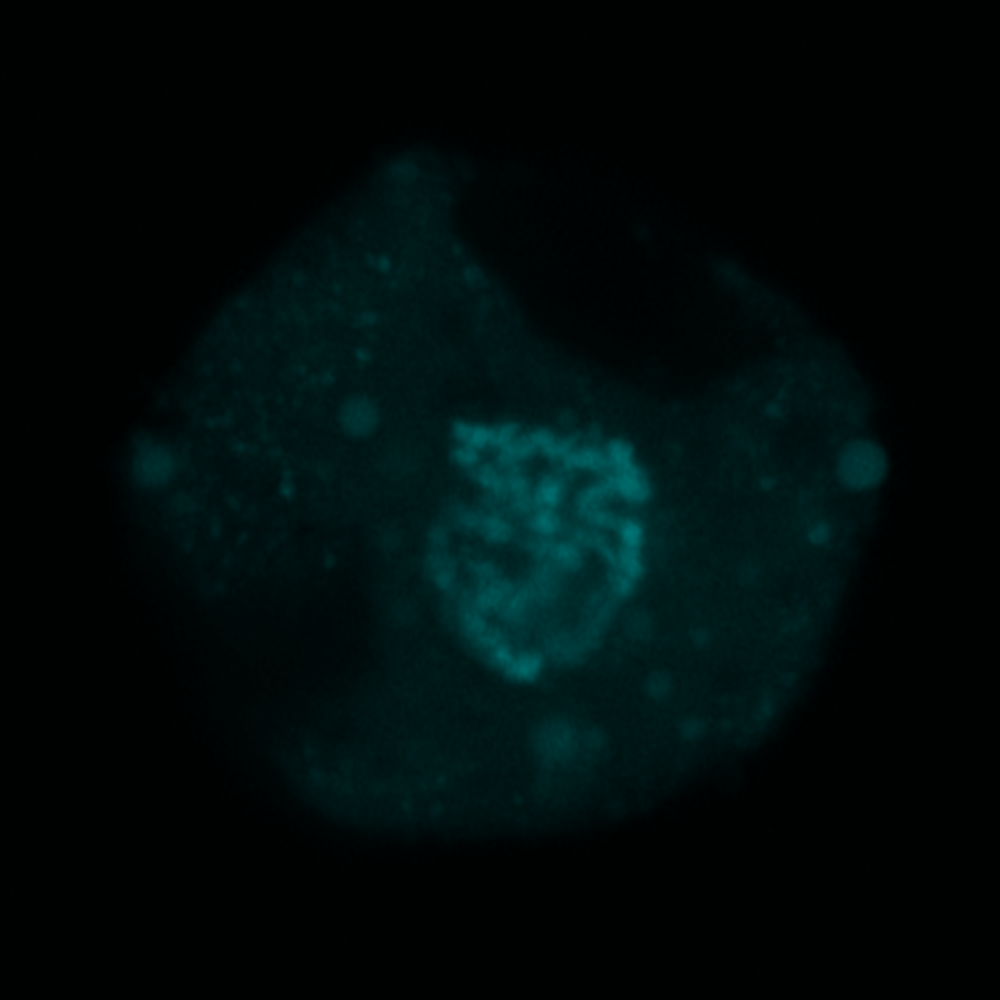

Supplement: Supplementary file 16 — Source_Data_zip [file 41467_2023_38881_MOESM16_ESM.zip › Source_Data_files_RK/SupFig_6/221031 NB SD 0.5A negative control PMC-2_Airyscan Processing_SupFig6a_e/221031 NB SD 0.5A negative control PMC-2_Airyscan Processing_ChA-T4.tif]

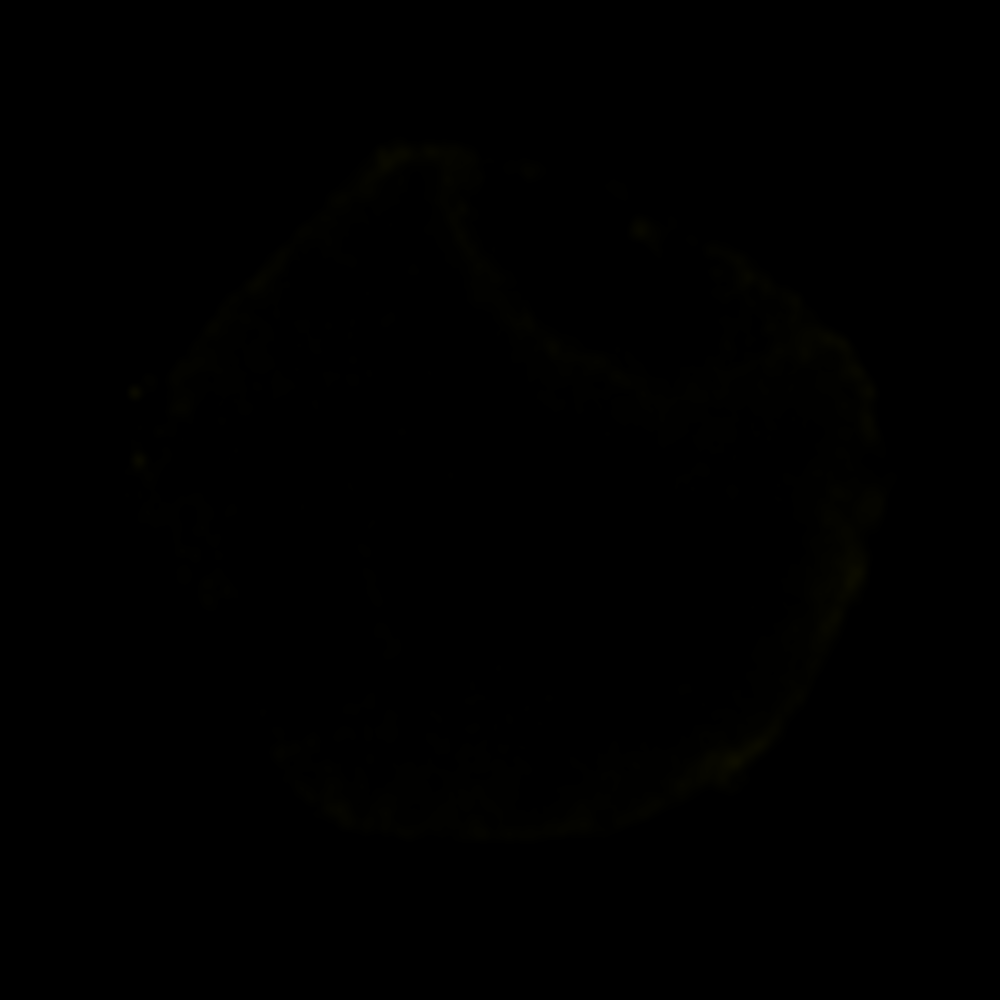

Supplement: Supplementary file 16 — Source_Data_zip [file 41467_2023_38881_MOESM16_ESM.zip › Source_Data_files_RK/SupFig_6/221031 NB SD 0.5A negative control PMC-2_Airyscan Processing_SupFig6a_e/221031 NB SD 0.5A negative control PMC-2_Airyscan Processing_ChA-T1.tif]

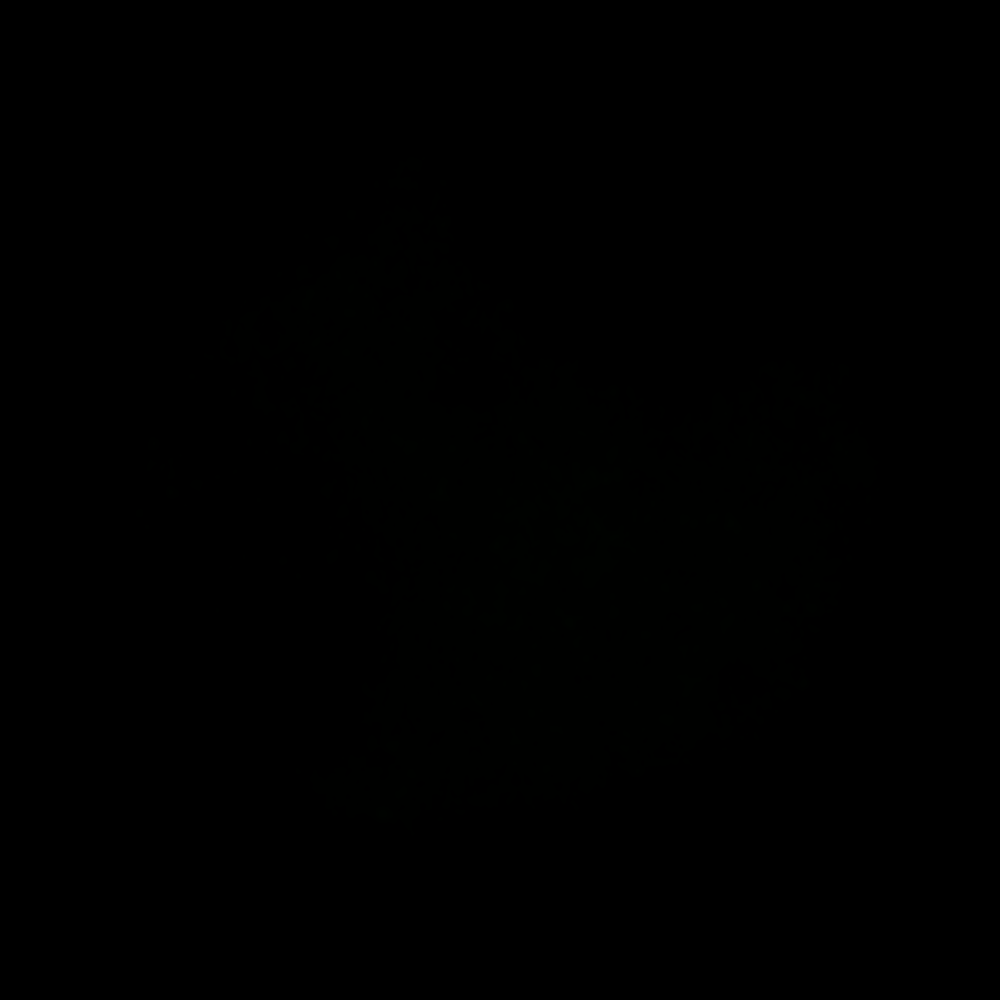

Supplement: Supplementary file 16 — Source_Data_zip [file 41467_2023_38881_MOESM16_ESM.zip › Source_Data_files_RK/SupFig_6/221031 NB SD 0.5A negative control PMC-2_Airyscan Processing_SupFig6a_e/221031 NB SD 0.5A negative control PMC-2_Airyscan Processing_ChA-T3.tif]

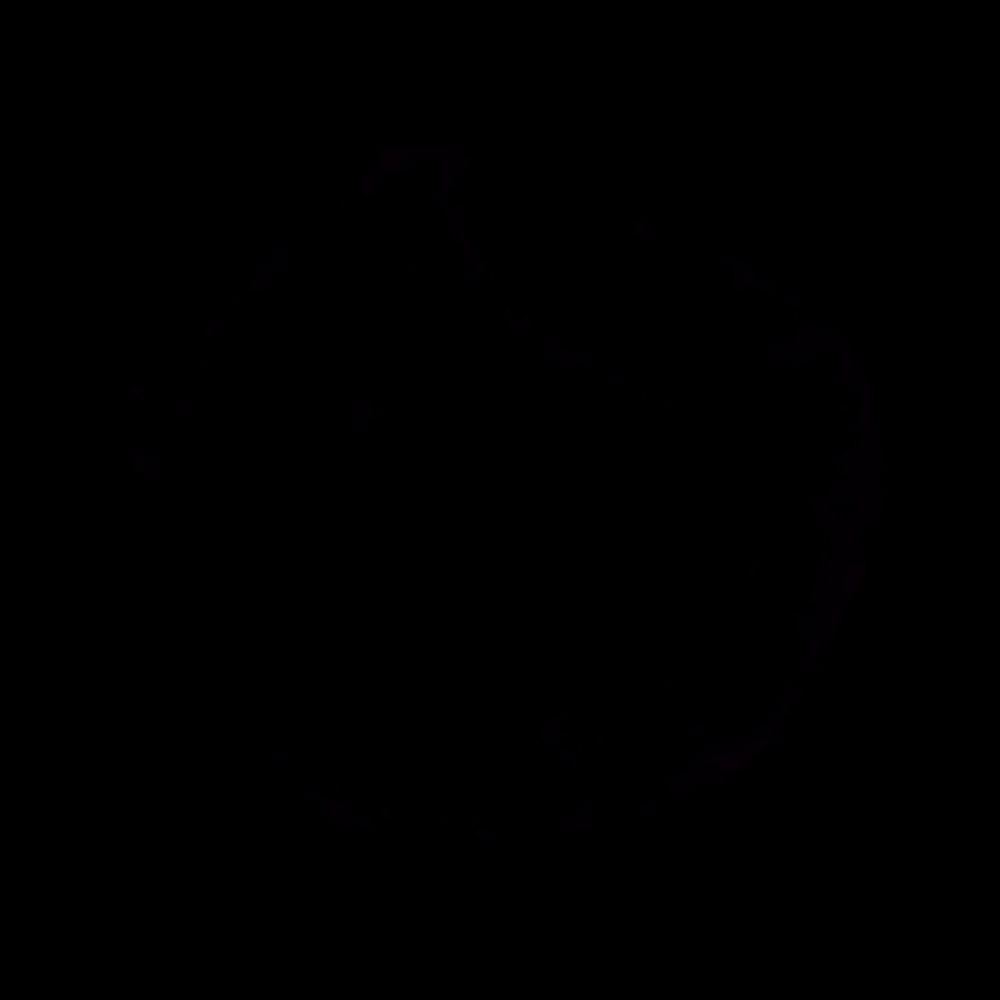

Supplement: Supplementary file 16 — Source_Data_zip [file 41467_2023_38881_MOESM16_ESM.zip › Source_Data_files_RK/SupFig_6/221031 NB SD 0.5A negative control PMC-2_Airyscan Processing_SupFig6a_e/221031 NB SD 0.5A negative control PMC-2_Airyscan Processing_ChA-T2.tif]

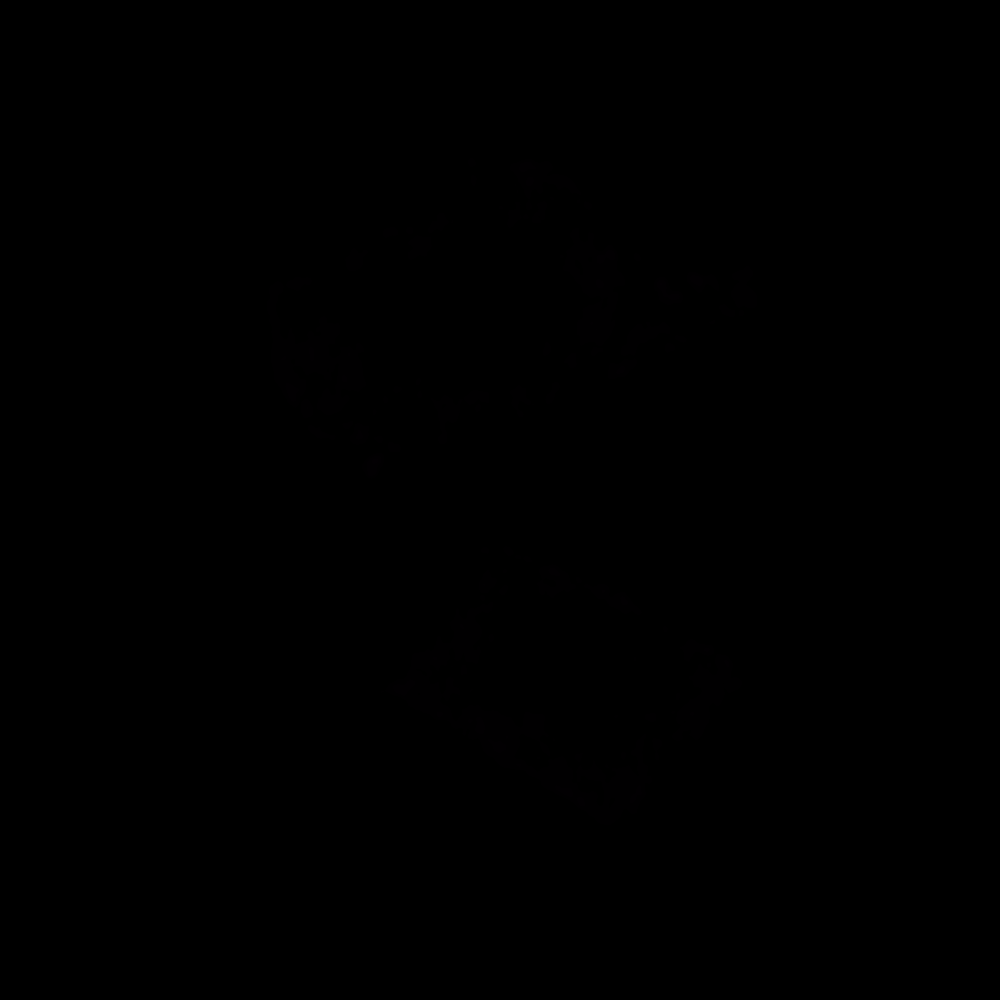

Supplement: Supplementary file 16 — Source_Data_zip [file 41467_2023_38881_MOESM16_ESM.zip › Source_Data_files_RK/SupFig_6/221031 NB SD 0.5A negative control SC-6_Airyscan ProcessingSupFig6f_j/221031 NB SD 0.5A negative control SC-6_Airyscan Processing_ChA-T2.tif]

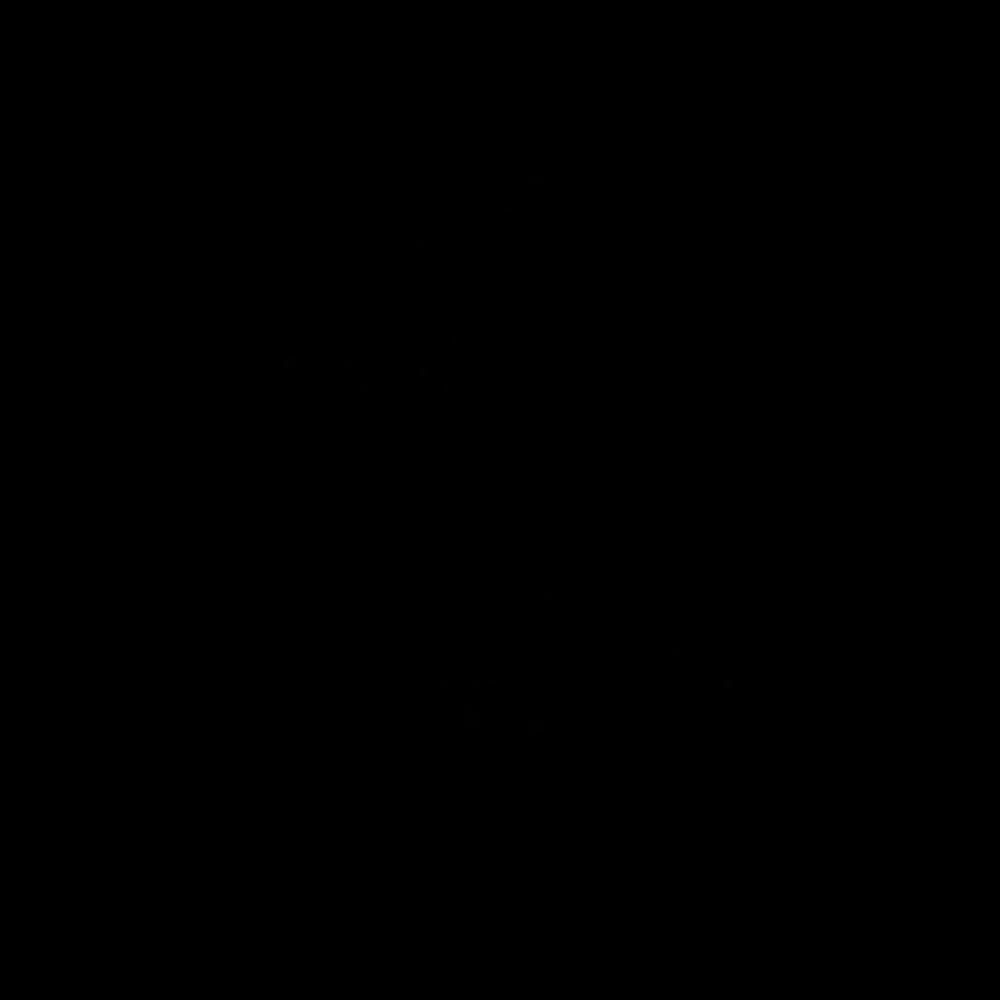

Supplement: Supplementary file 16 — Source_Data_zip [file 41467_2023_38881_MOESM16_ESM.zip › Source_Data_files_RK/SupFig_6/221031 NB SD 0.5A negative control SC-6_Airyscan ProcessingSupFig6f_j/221031 NB SD 0.5A negative control SC-6_Airyscan Processing_ChA-T3.tif]

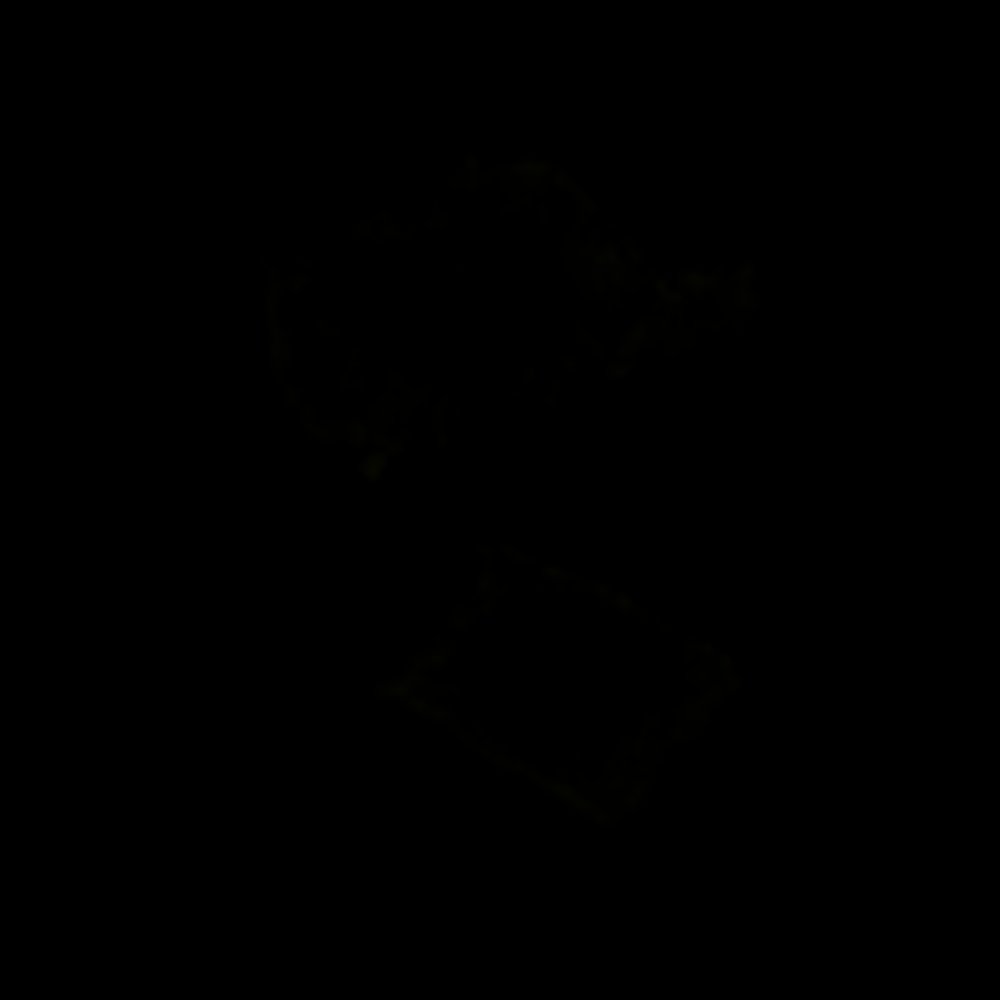

Supplement: Supplementary file 16 — Source_Data_zip [file 41467_2023_38881_MOESM16_ESM.zip › Source_Data_files_RK/SupFig_6/221031 NB SD 0.5A negative control SC-6_Airyscan ProcessingSupFig6f_j/221031 NB SD 0.5A negative control SC-6_Airyscan Processing_ChA-T1.tif]

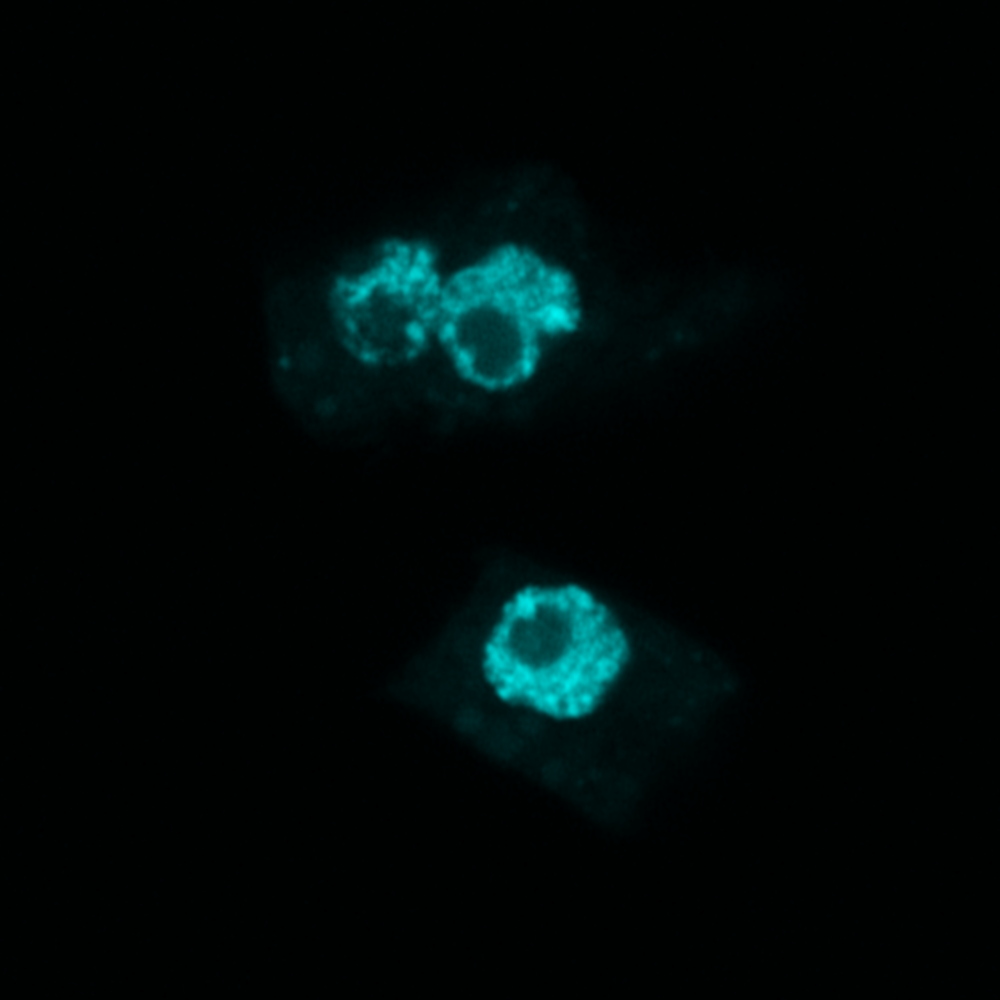

Supplement: Supplementary file 16 — Source_Data_zip [file 41467_2023_38881_MOESM16_ESM.zip › Source_Data_files_RK/SupFig_6/221031 NB SD 0.5A negative control SC-6_Airyscan ProcessingSupFig6f_j/221031 NB SD 0.5A negative control SC-6_Airyscan Processing_ChA-T4.tif]

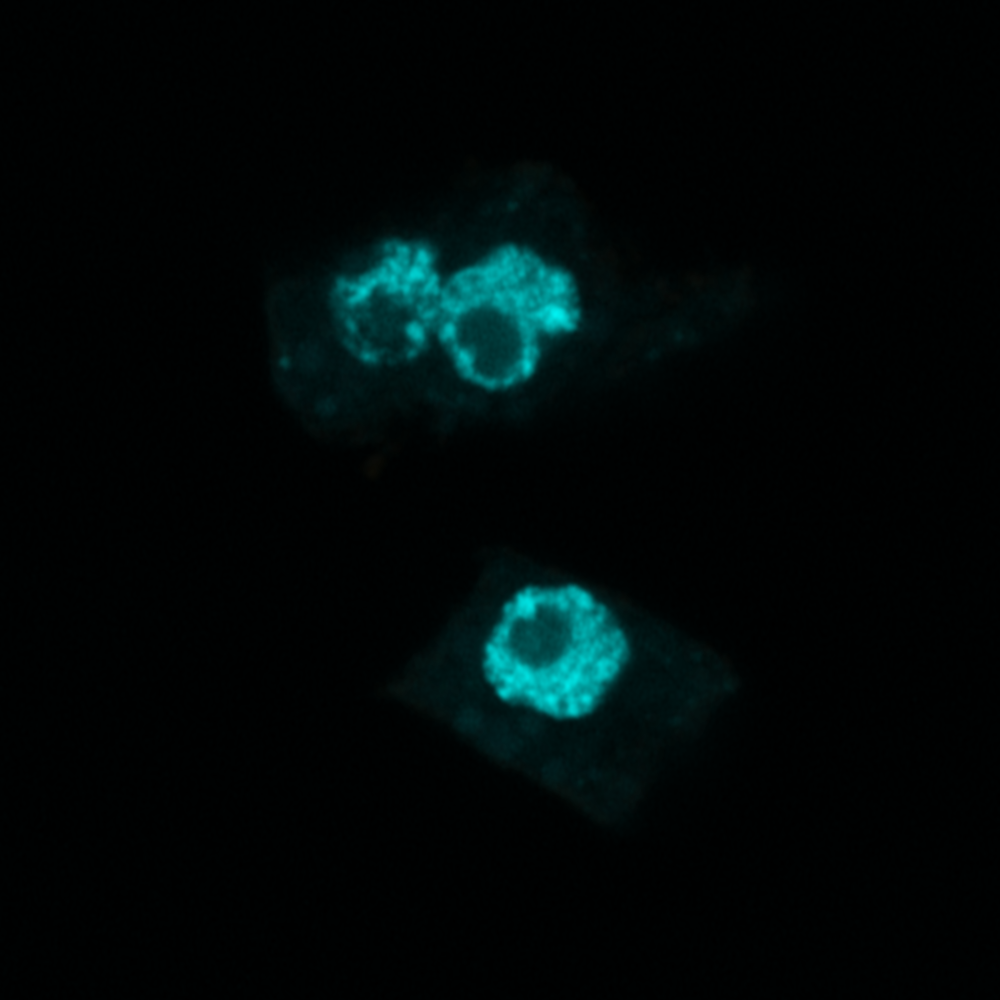

Supplement: Supplementary file 16 — Source_Data_zip [file 41467_2023_38881_MOESM16_ESM.zip › Source_Data_files_RK/SupFig_6/221031 NB SD 0.5A negative control SC-6_Airyscan ProcessingSupFig6f_j/221031 NB SD 0.5A negative control SC-6_Airyscan Processing.tif]

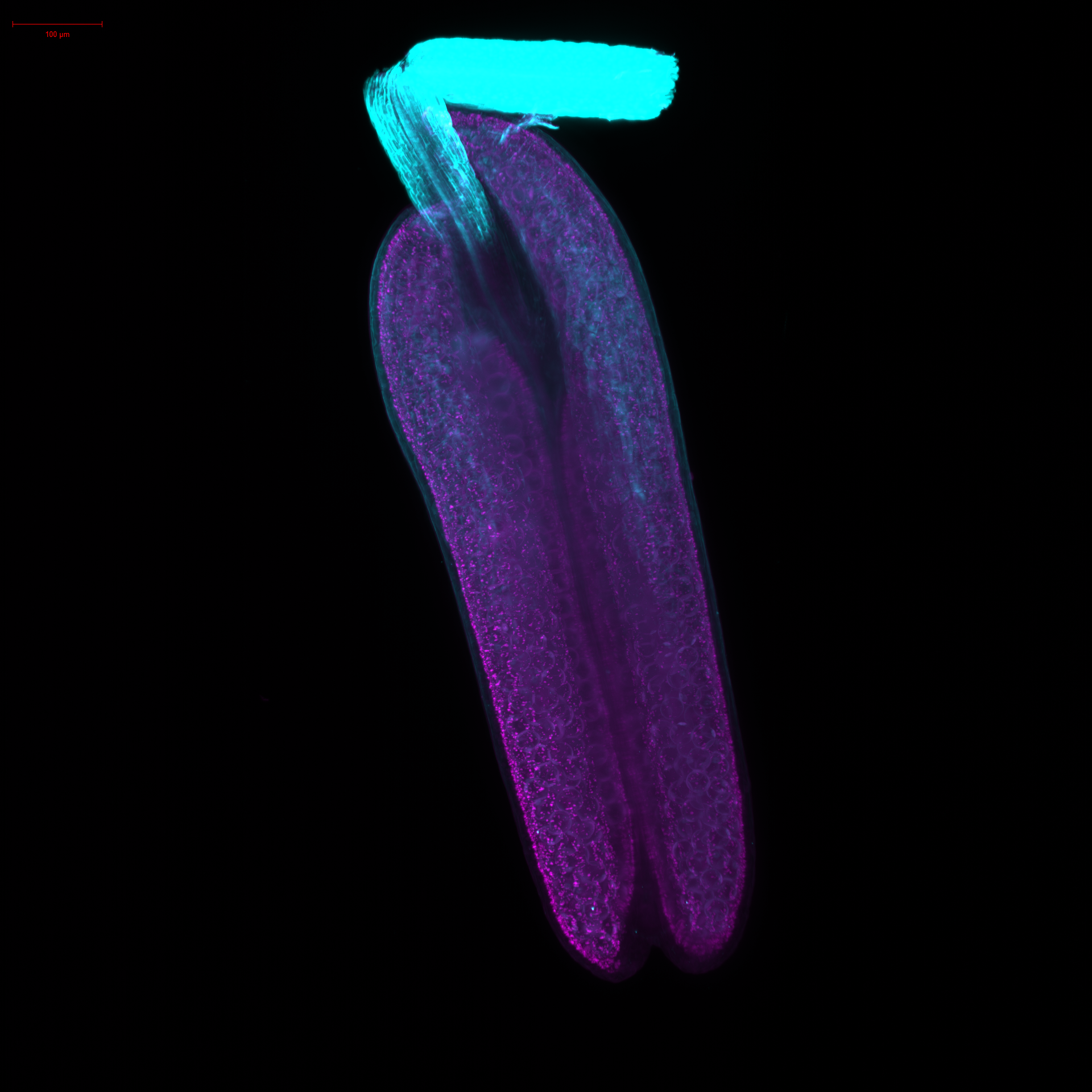

Supplement: Supplementary file 16 — Source_Data_zip [file 41467_2023_38881_MOESM16_ESM.zip › Source_Data_files_RK/Fig_1/Fig1_h-o_3Danther/Fig1_h2WT98mmSRPI20x036xNo2a15195MIPD195tif.tif]

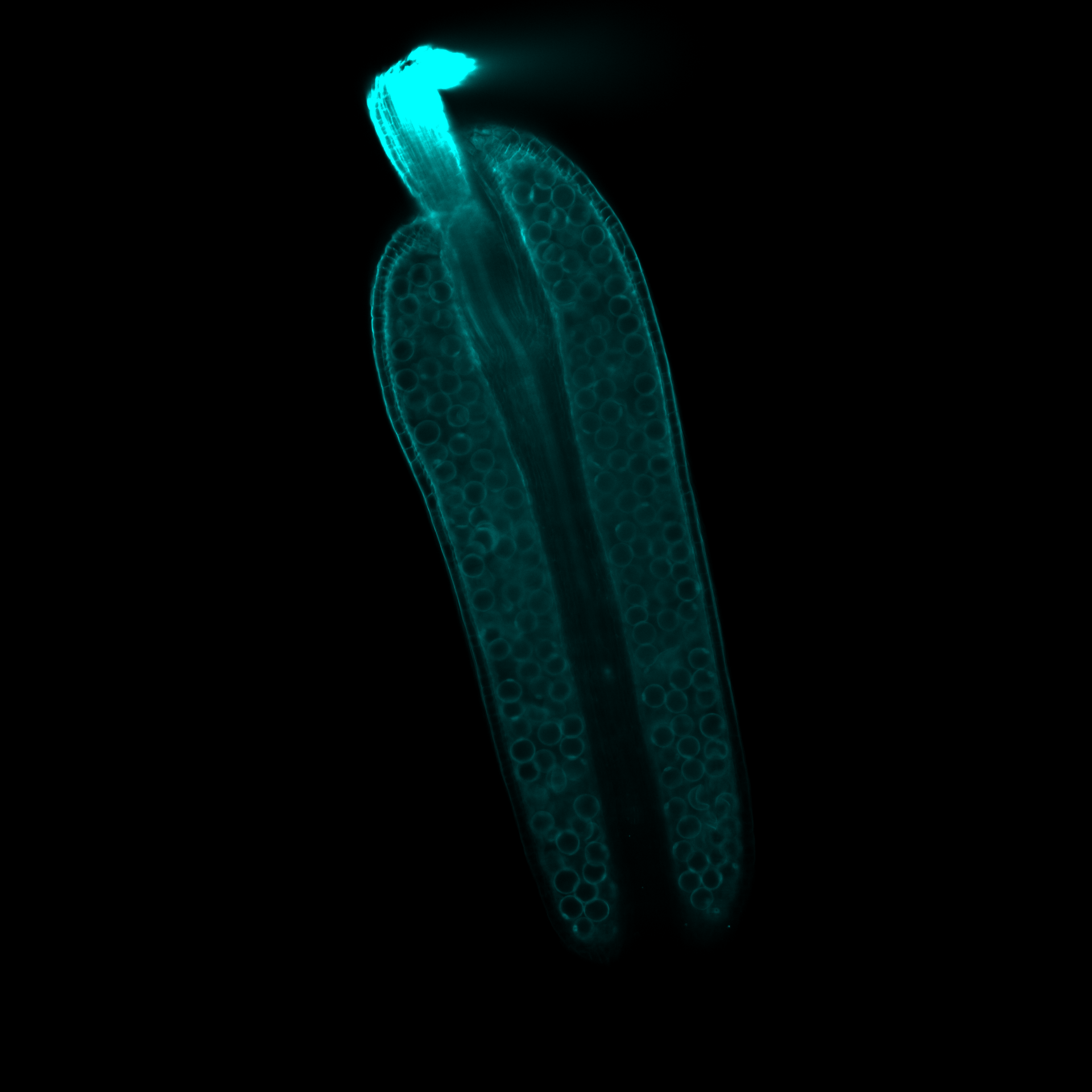

Supplement: Supplementary file 16 — Source_Data_zip [file 41467_2023_38881_MOESM16_ESM.zip › Source_Data_files_RK/Fig_1/Fig1_h-o_3Danther/Fig1_i_2WT98mmSRPI20x036xNo2a15195s163SR2200.tif]

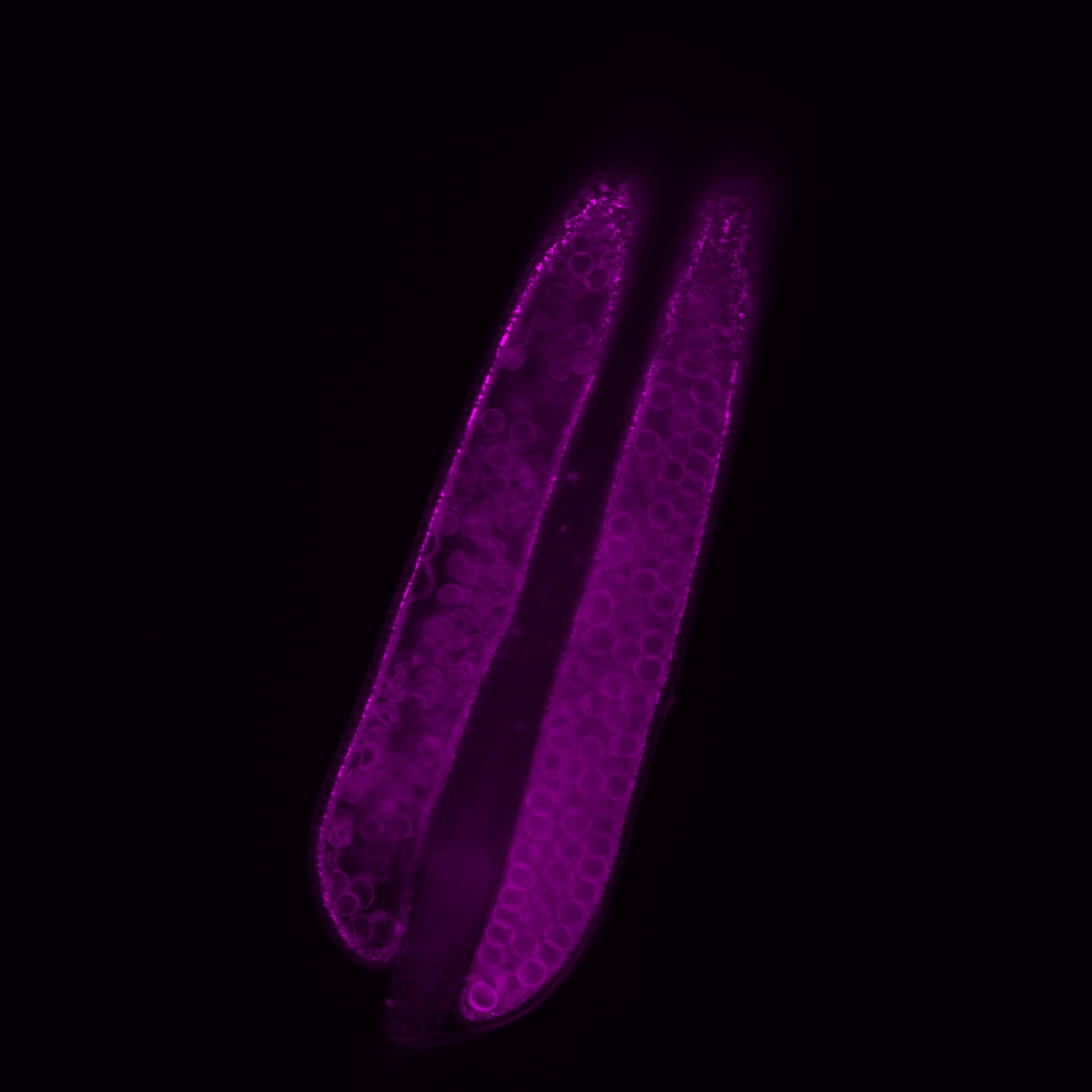

Supplement: Supplementary file 16 — Source_Data_zip [file 41467_2023_38881_MOESM16_ESM.zip › Source_Data_files_RK/Fig_1/Fig1_h-o_3Danther/Fig1_n_5aa90mmSRPI20x036xNo2a70250a20S205PI.tif]

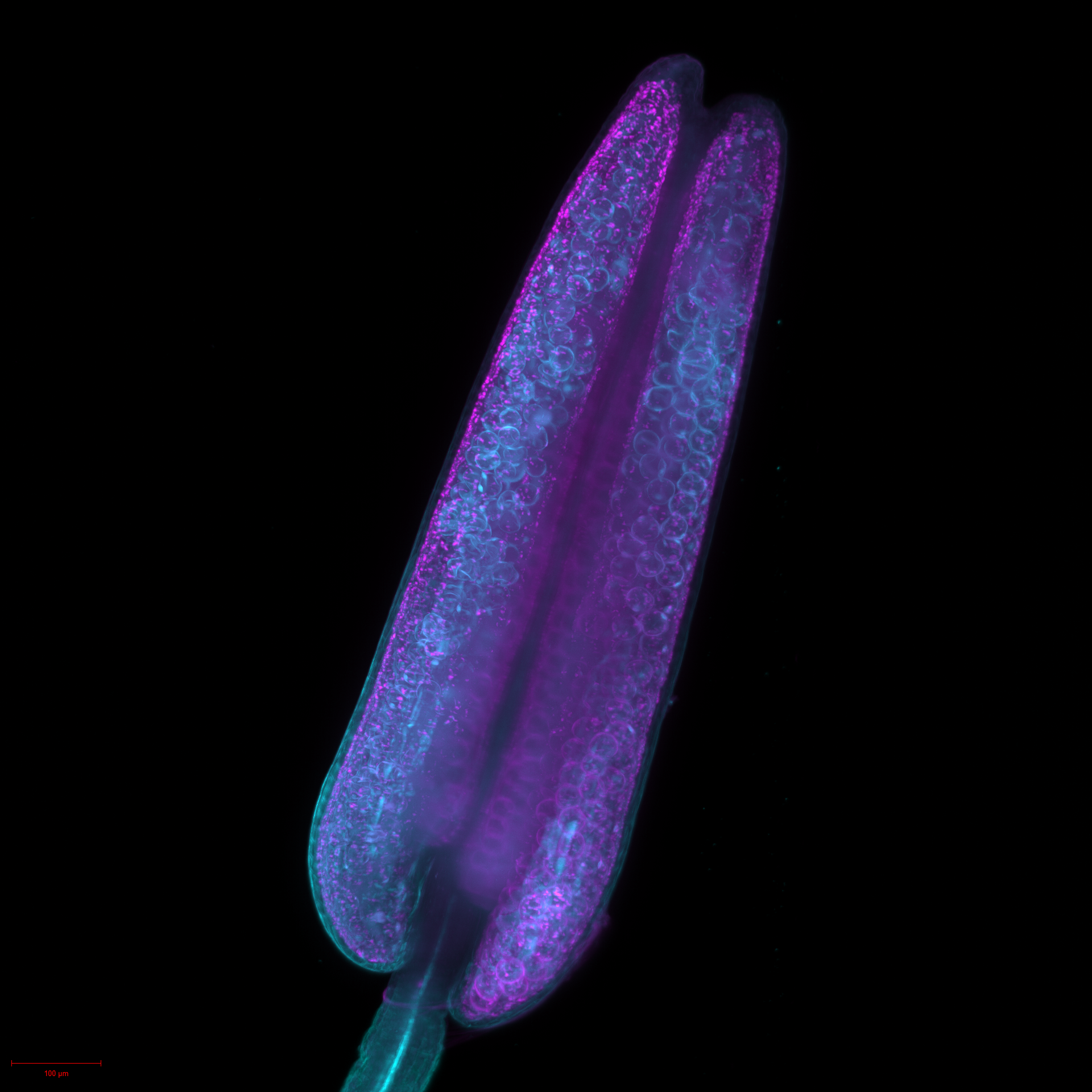

Supplement: Supplementary file 16 — Source_Data_zip [file 41467_2023_38881_MOESM16_ESM.zip › Source_Data_files_RK/Fig_1/Fig1_h-o_3Danther/Fig1_l_5aa90mmSRPI20x036xNo2a70250MIPD70.tif]

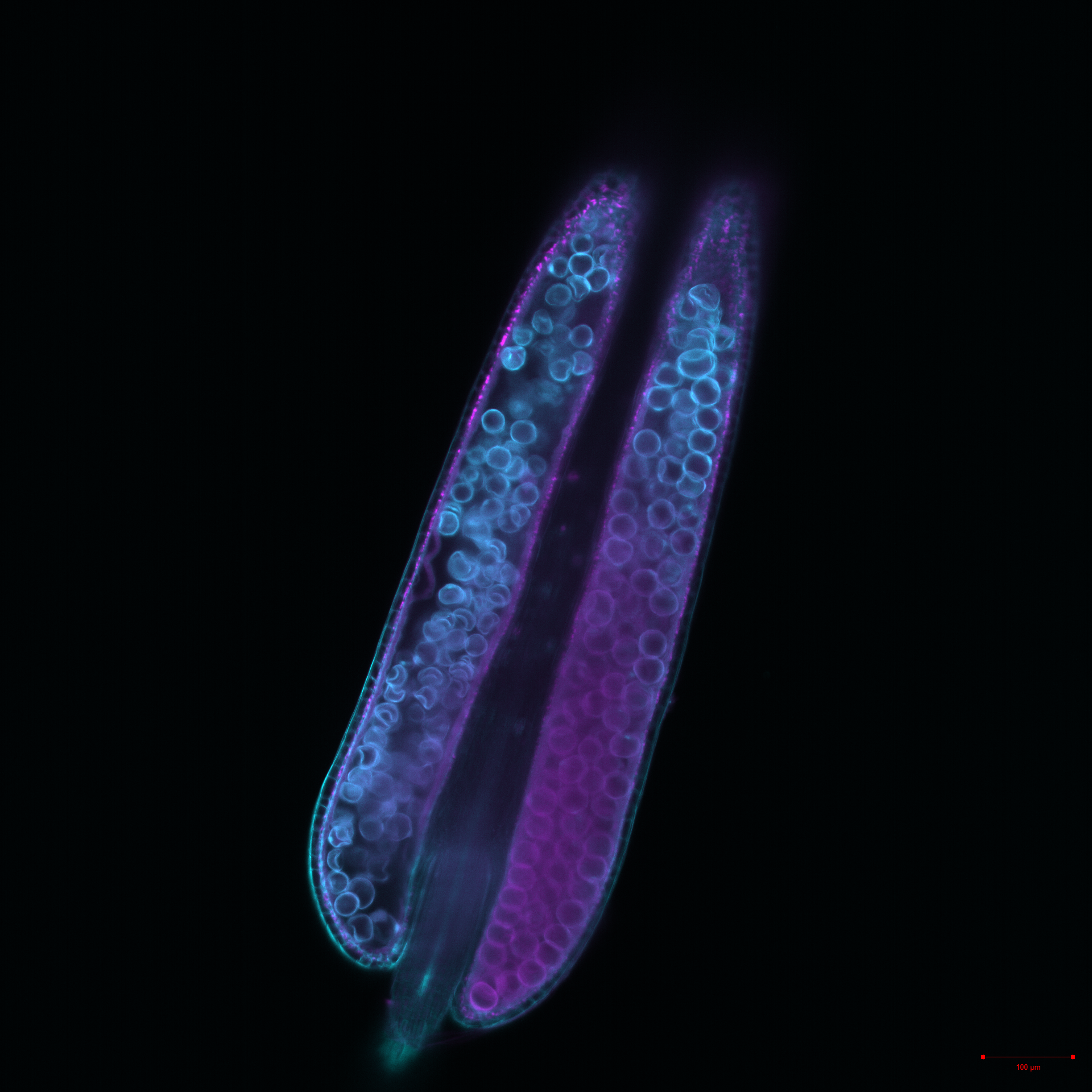

Supplement: Supplementary file 16 — Source_Data_zip [file 41467_2023_38881_MOESM16_ESM.zip › Source_Data_files_RK/Fig_1/Fig1_h-o_3Danther/Fig1_o_5aa90mmSRPI20x036xNo2a70250D70S205.tif]

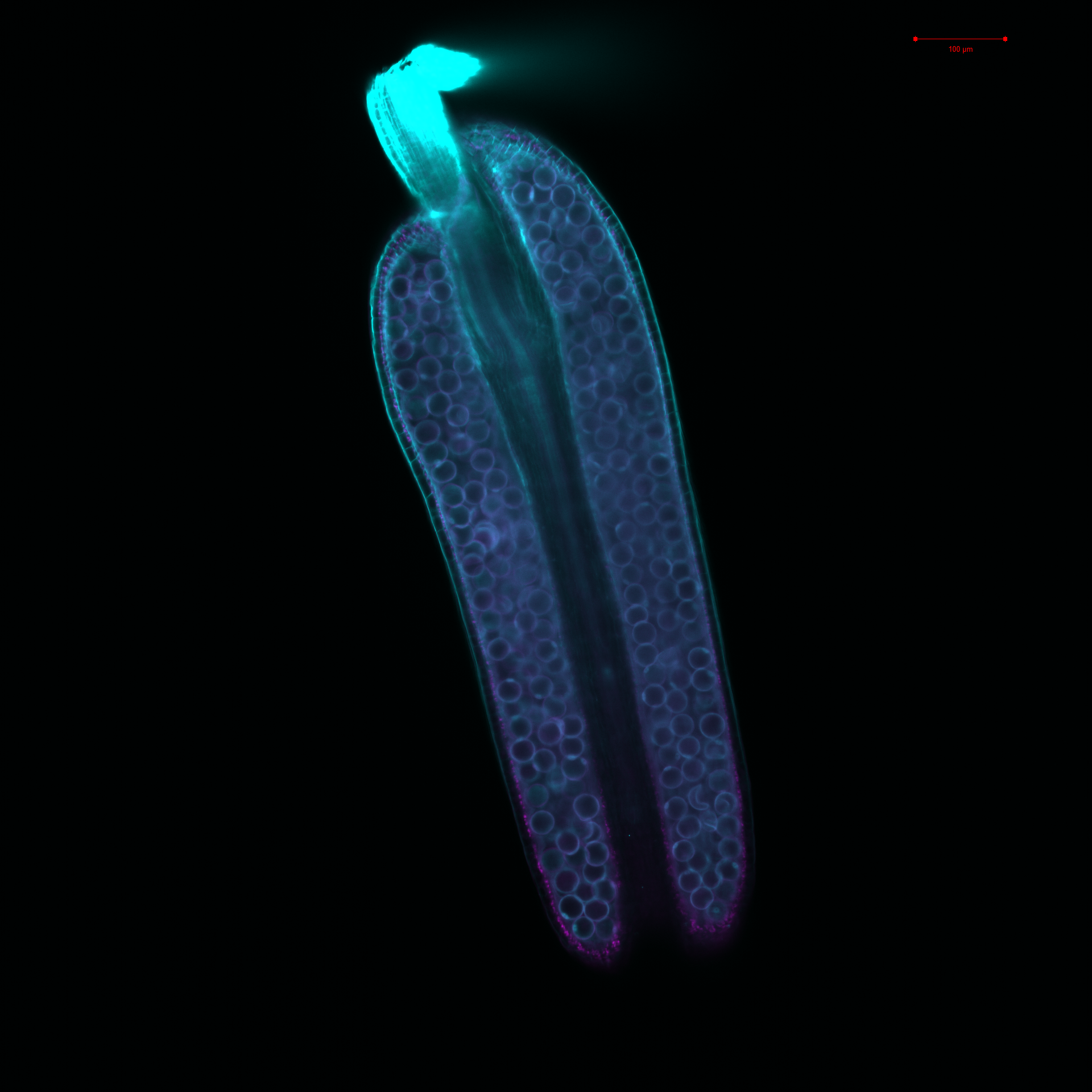

Supplement: Supplementary file 16 — Source_Data_zip [file 41467_2023_38881_MOESM16_ESM.zip › Source_Data_files_RK/Fig_1/Fig1_h-o_3Danther/Fig1_k2WT98mmSRPI20x036xNo2a15195D195S163.tif]

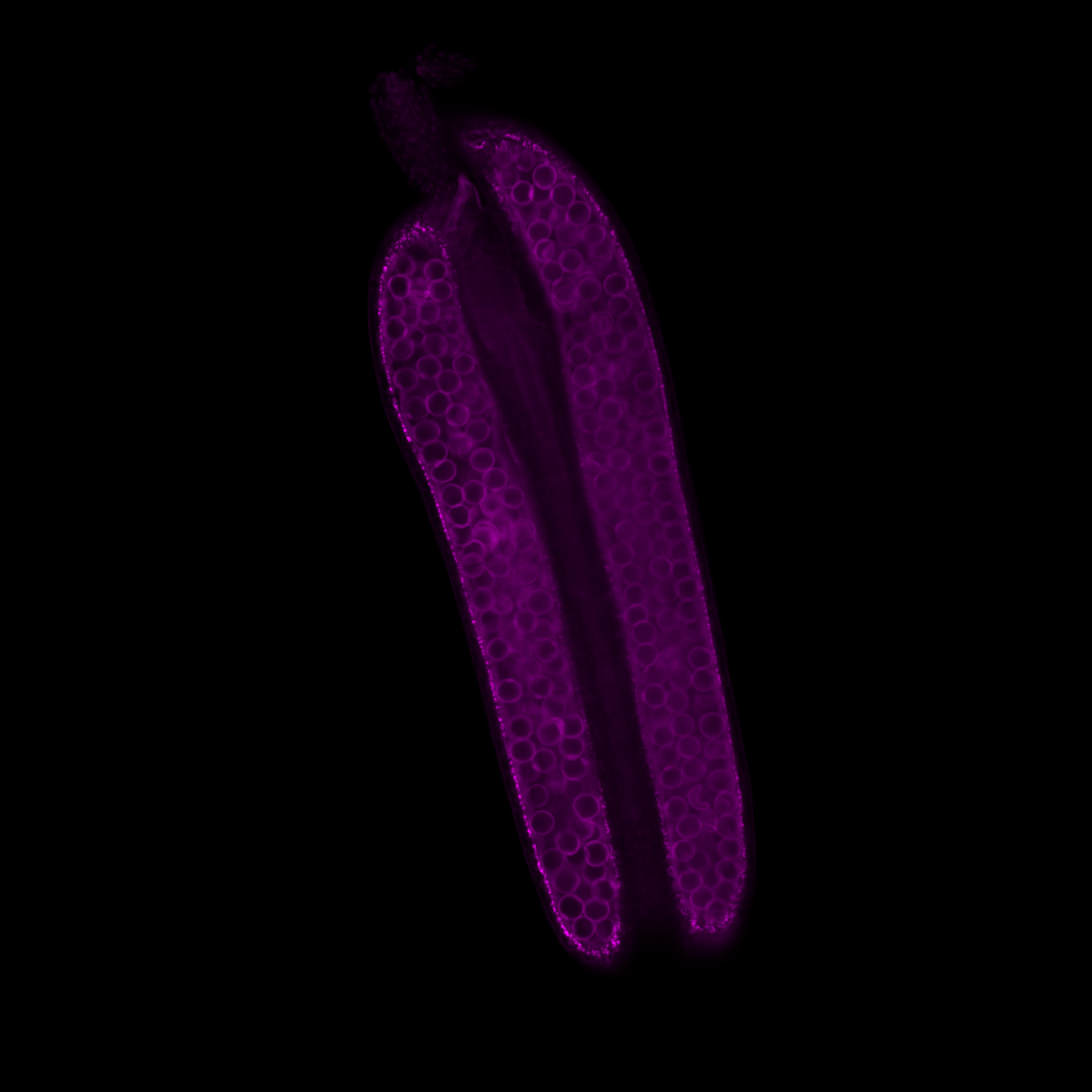

Supplement: Supplementary file 16 — Source_Data_zip [file 41467_2023_38881_MOESM16_ESM.zip › Source_Data_files_RK/Fig_1/Fig1_h-o_3Danther/Fig1_j_2WT98mmSRPI20x036xNo2a15195s163PI.tif]

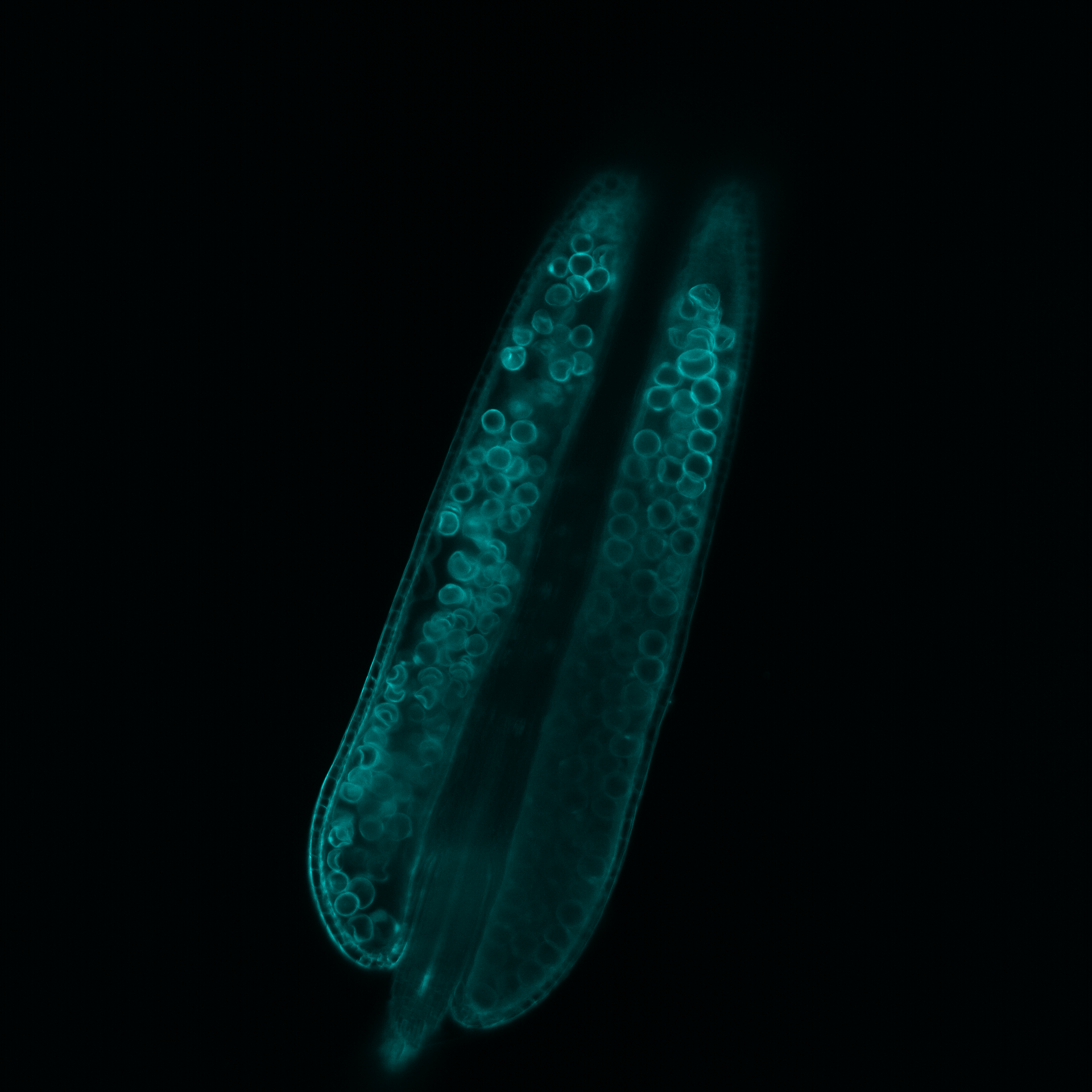

Supplement: Supplementary file 16 — Source_Data_zip [file 41467_2023_38881_MOESM16_ESM.zip › Source_Data_files_RK/Fig_1/Fig1_h-o_3Danther/Fig1_m5aa90mmSRPI20x036xNo2a70250a70S205SR2200.tif]

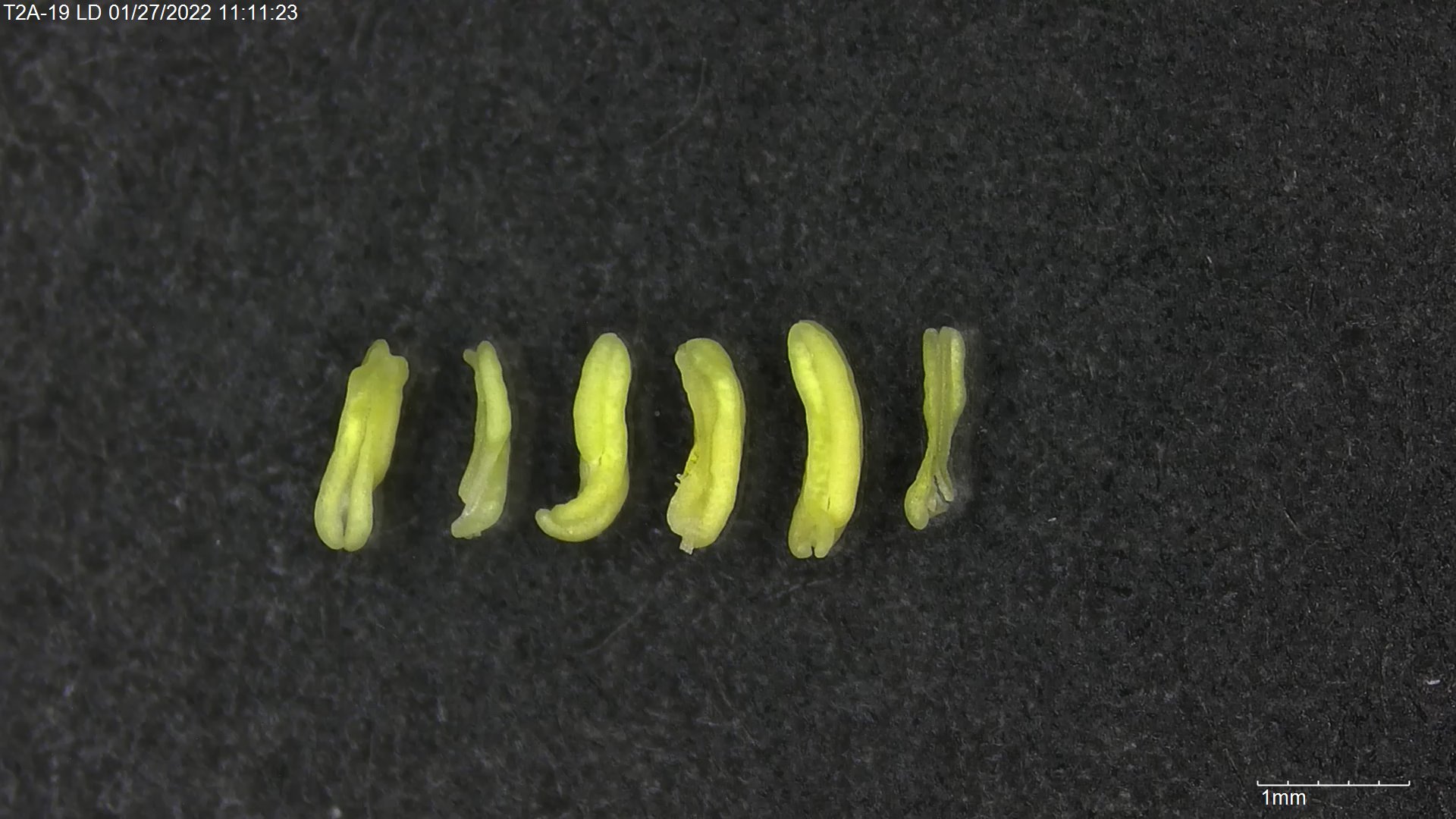

Supplement: Supplementary file 16 — Source_Data_zip [file 41467_2023_38881_MOESM16_ESM.zip › Source_Data_files_RK/Fig_1/Fig1_d-g/Fig1_e_aa d3d1-123 anther-3.jpg]

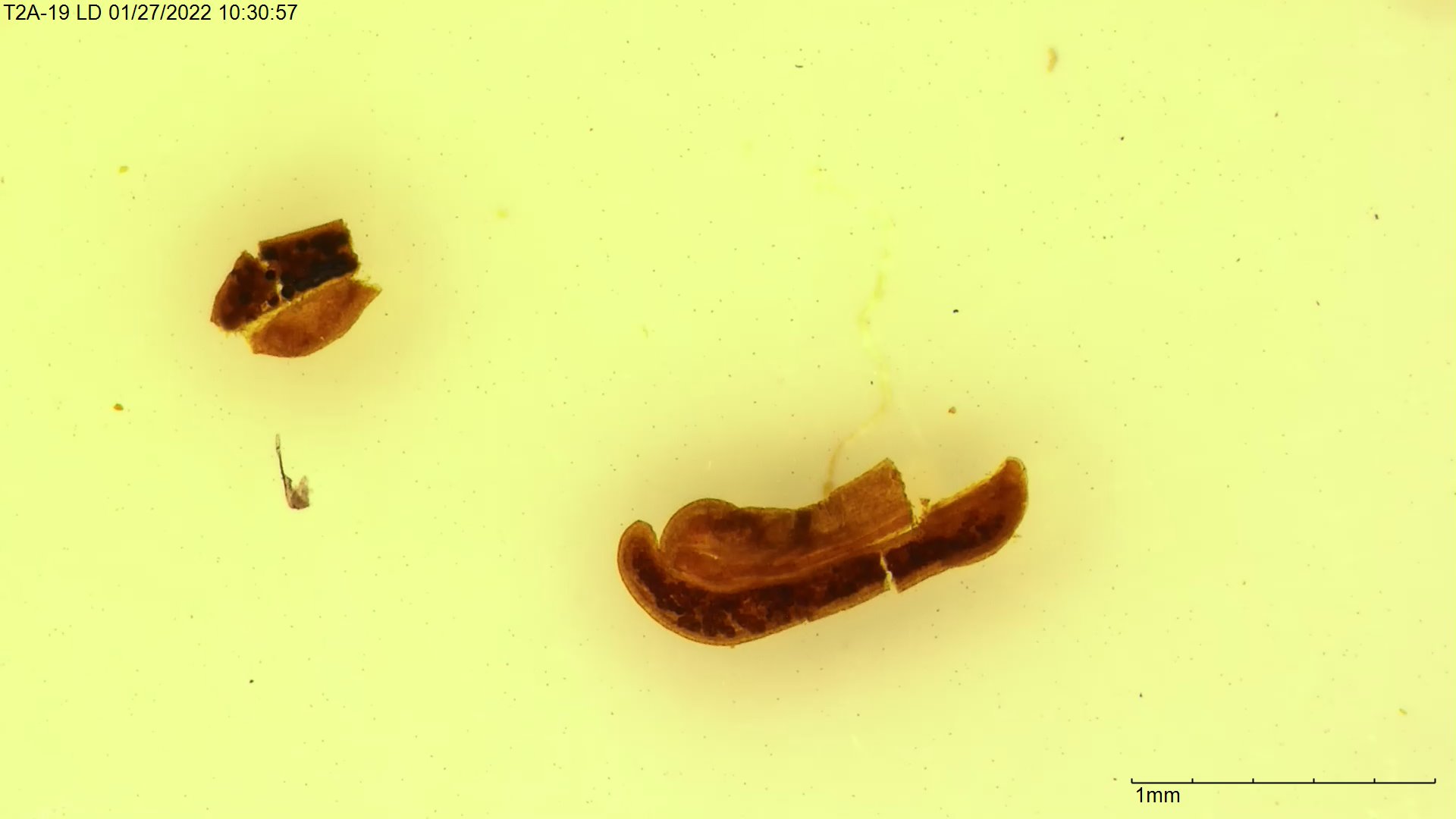

Supplement: Supplementary file 16 — Source_Data_zip [file 41467_2023_38881_MOESM16_ESM.zip › Source_Data_files_RK/Fig_1/Fig1_d-g/Fig1_g_aa d3d1-123 pollenx2-5.jpg]

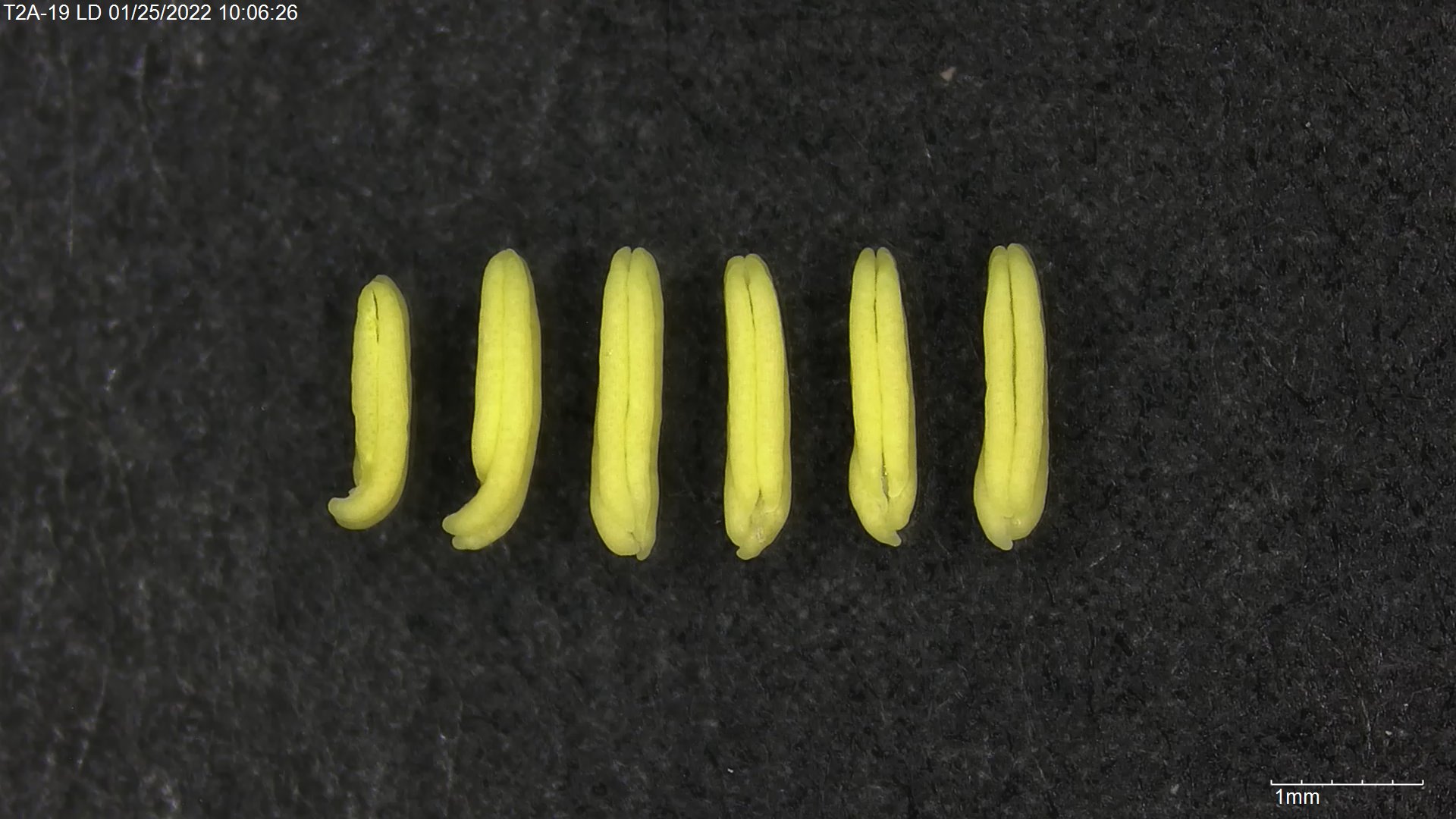

Supplement: Supplementary file 16 — Source_Data_zip [file 41467_2023_38881_MOESM16_ESM.zip › Source_Data_files_RK/Fig_1/Fig1_d-g/Fig1_d_WT d3d1-173 anther-2.jpg]

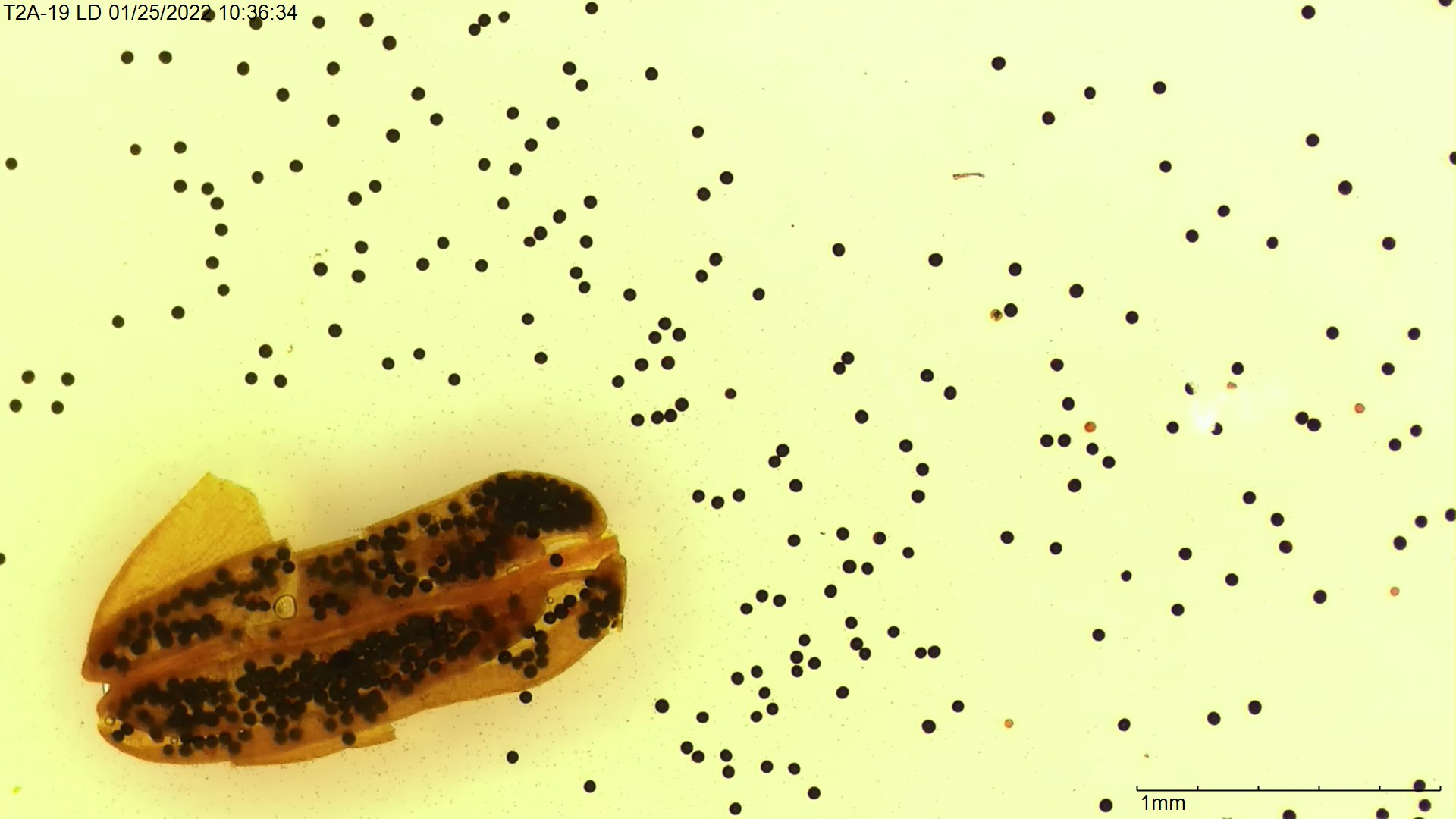

Supplement: Supplementary file 16 — Source_Data_zip [file 41467_2023_38881_MOESM16_ESM.zip › Source_Data_files_RK/Fig_1/Fig1_d-g/Fig1_f_WT d3d1-173 pollenx2-2.jpg]

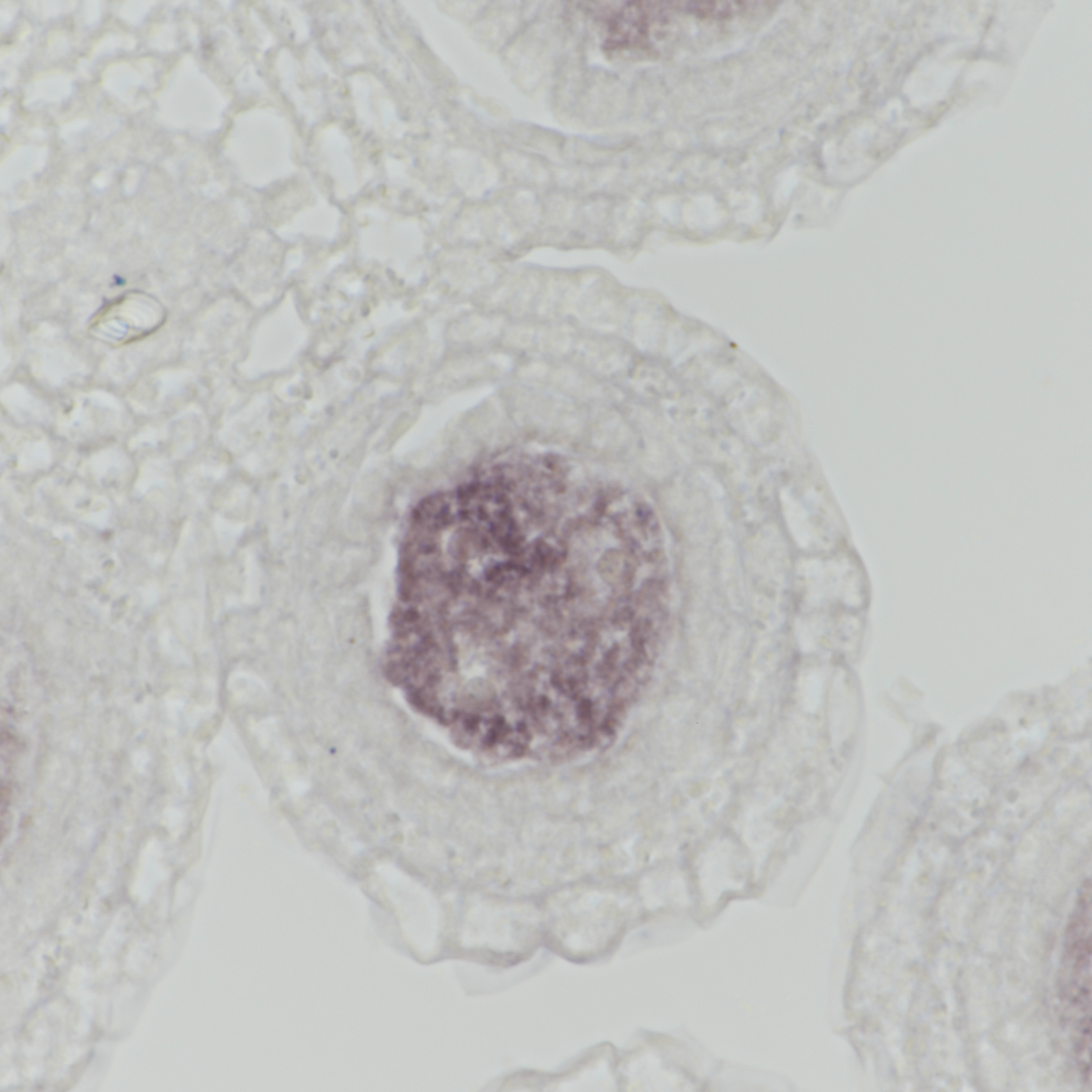

Supplement: Supplementary file 16 — Source_Data_zip [file 41467_2023_38881_MOESM16_ESM.zip › Source_Data_files_RK/Fig_6/Fig_6_a-c/MEL1.tif]

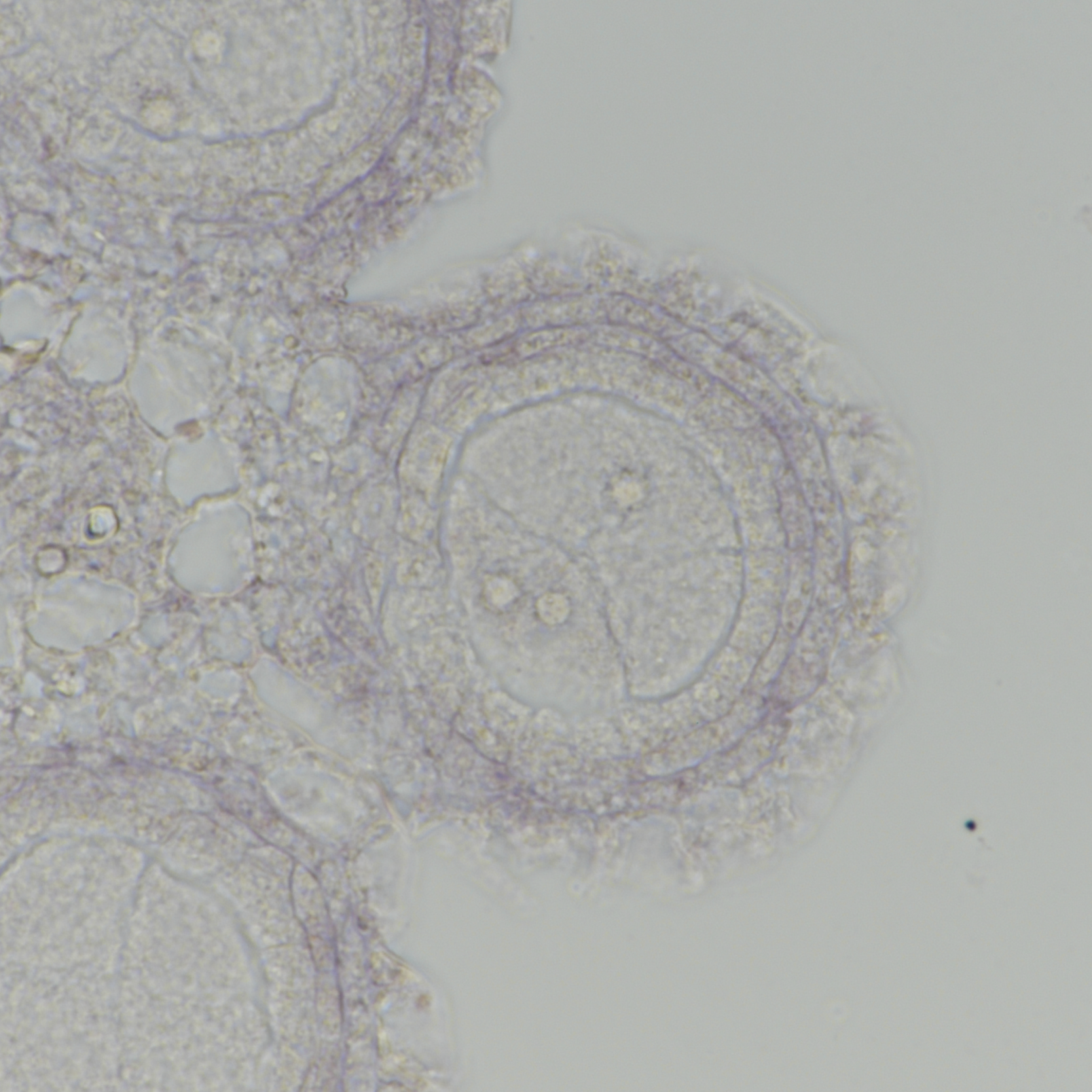

Supplement: Supplementary file 16 — Source_Data_zip [file 41467_2023_38881_MOESM16_ESM.zip › Source_Data_files_RK/Fig_6/Fig_6_a-c/AGO1b.tif]

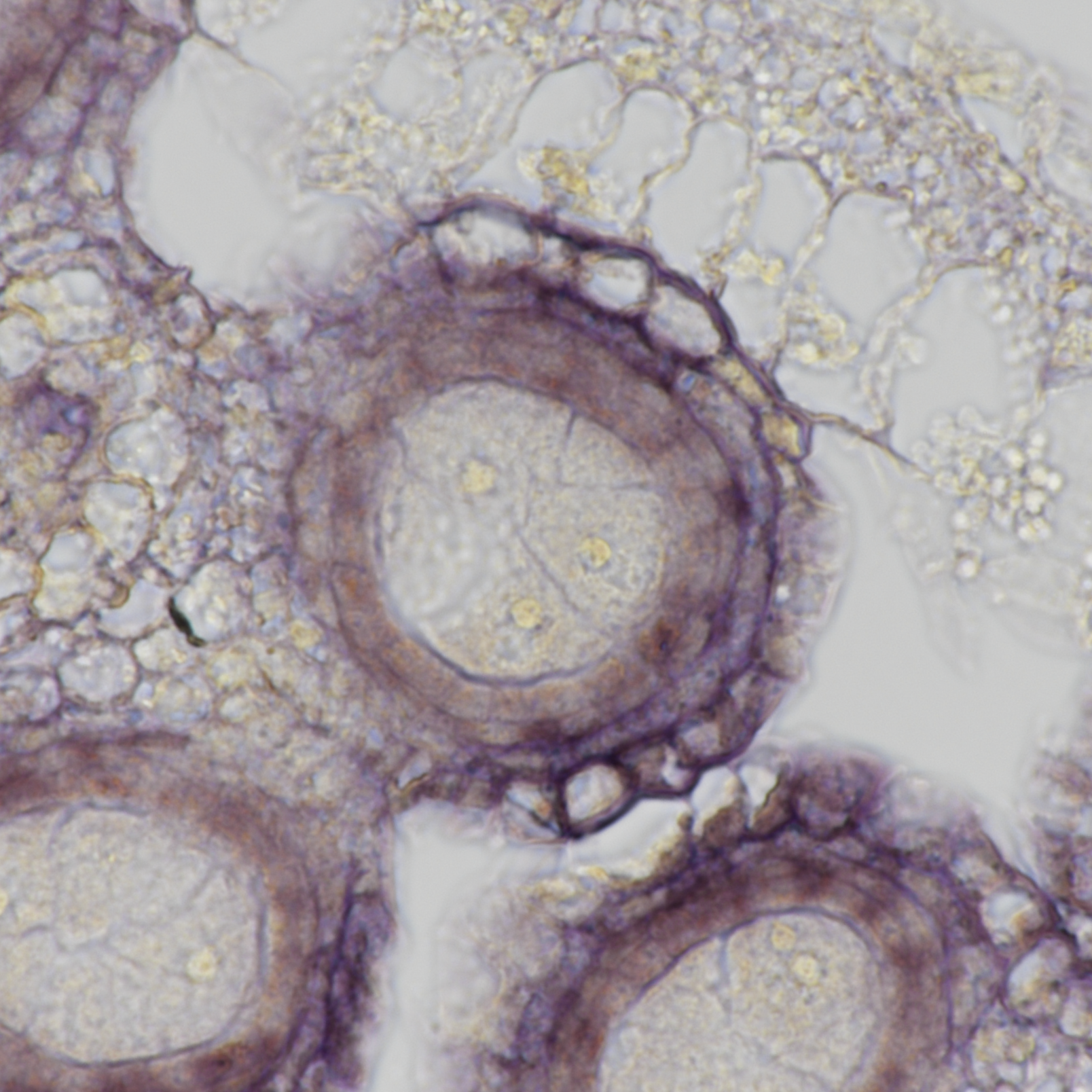

Supplement: Supplementary file 16 — Source_Data_zip [file 41467_2023_38881_MOESM16_ESM.zip › Source_Data_files_RK/Fig_6/Fig_6_a-c/AGO1d_revise2.tif]

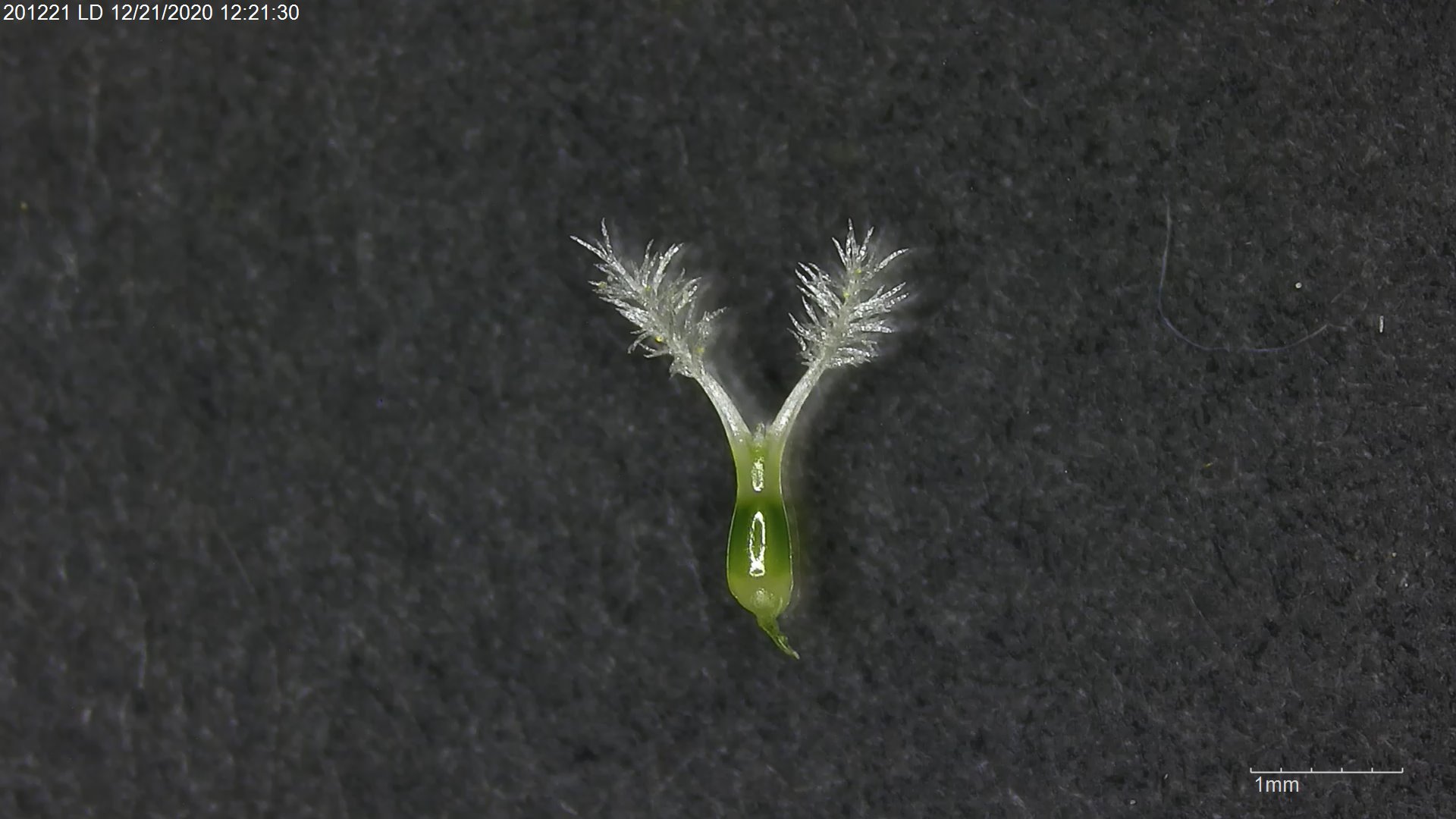

Supplement: Supplementary file 16 — Source_Data_zip [file 41467_2023_38881_MOESM16_ESM.zip › Source_Data_files_RK/Sup Fig_2/SupFig2_b_LD d g4-3-7-2 20dele pistil2.jpg]

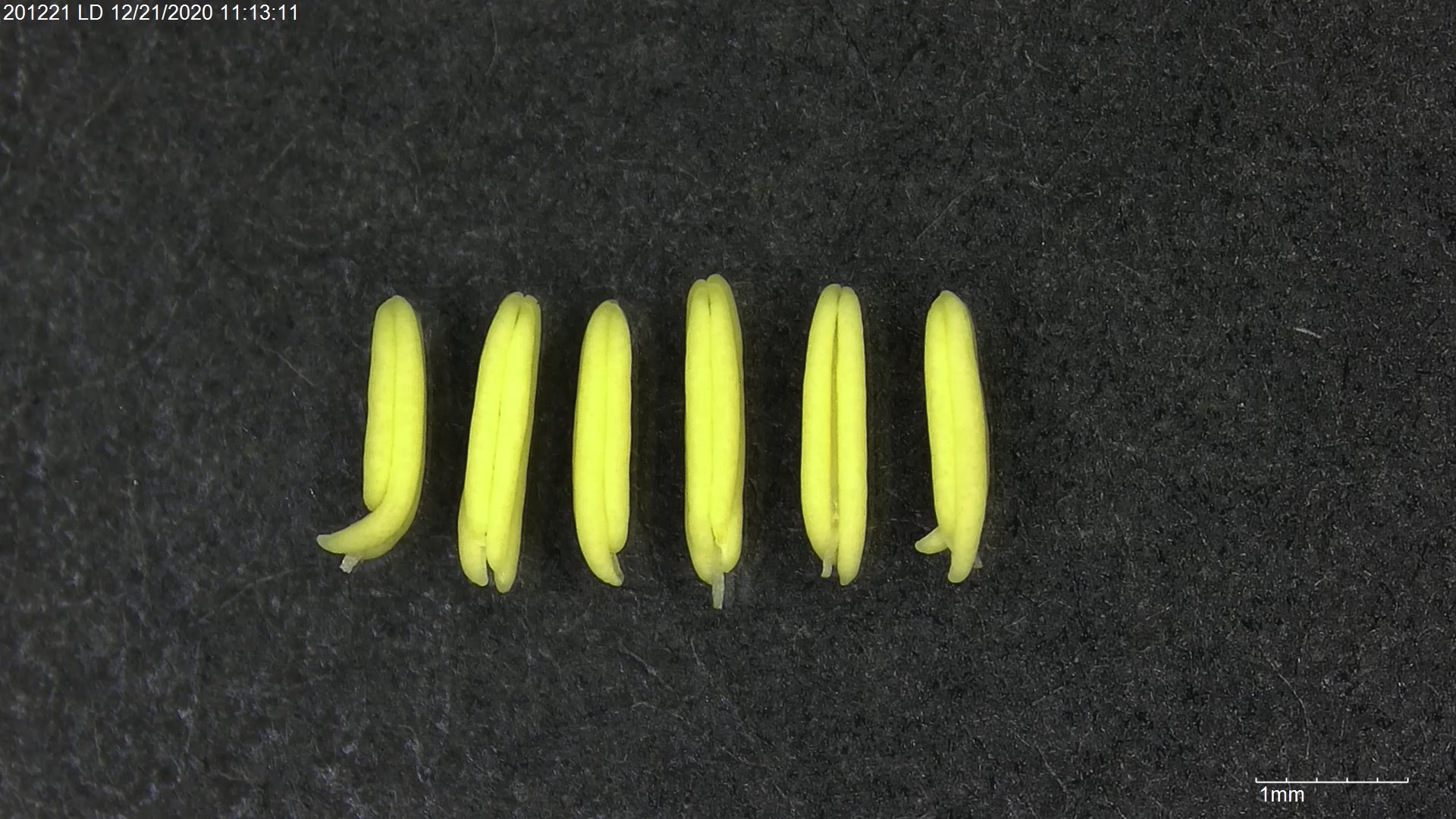

Supplement: Supplementary file 16 — Source_Data_zip [file 41467_2023_38881_MOESM16_ESM.zip › Source_Data_files_RK/Sup Fig_2/SupFig2_b_LD NB anther1.jpg]

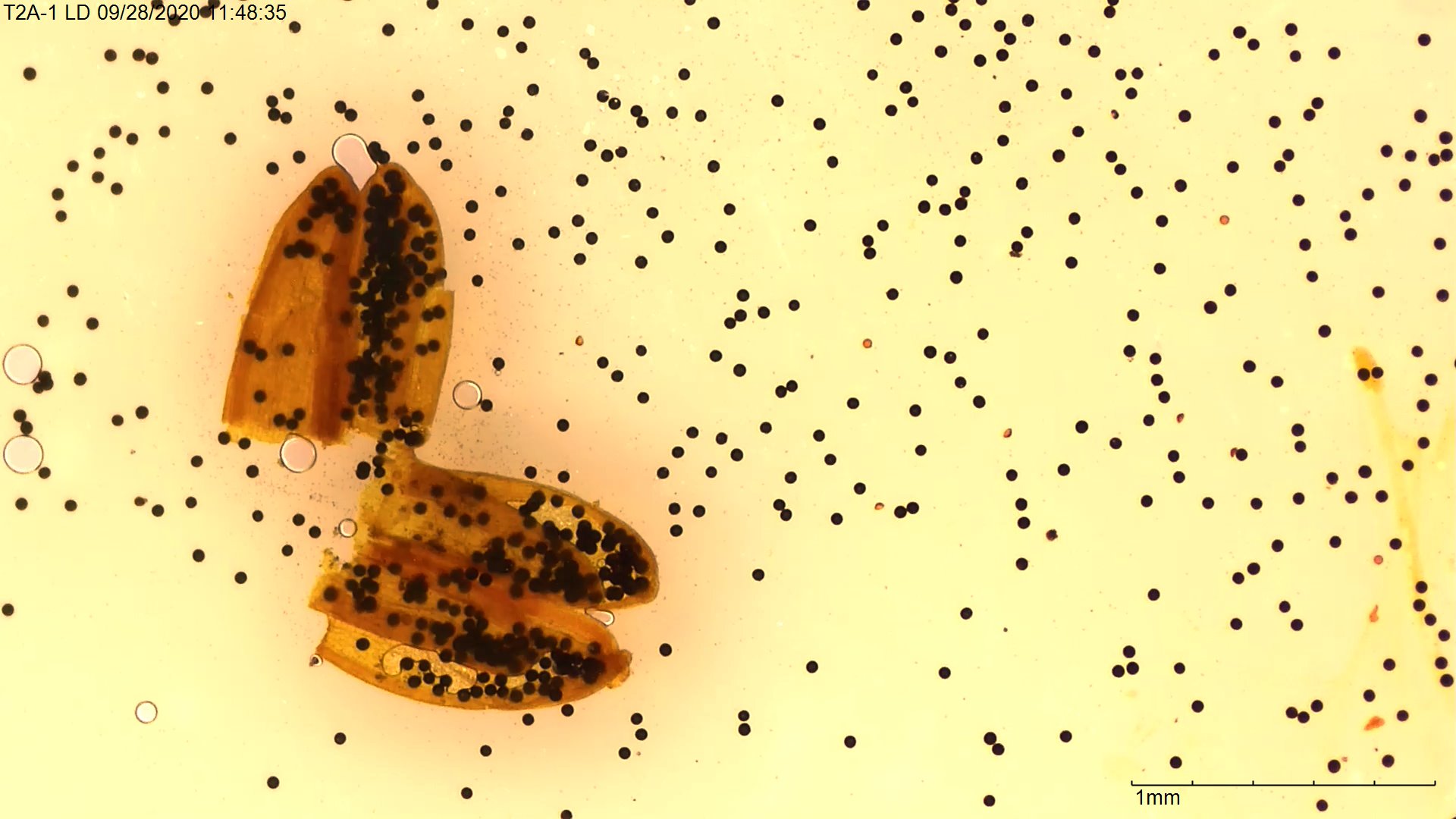

Supplement: Supplementary file 16 — Source_Data_zip [file 41467_2023_38881_MOESM16_ESM.zip › Source_Data_files_RK/Sup Fig_2/SupFig2_b_LD NB pollenx2-1.jpg]

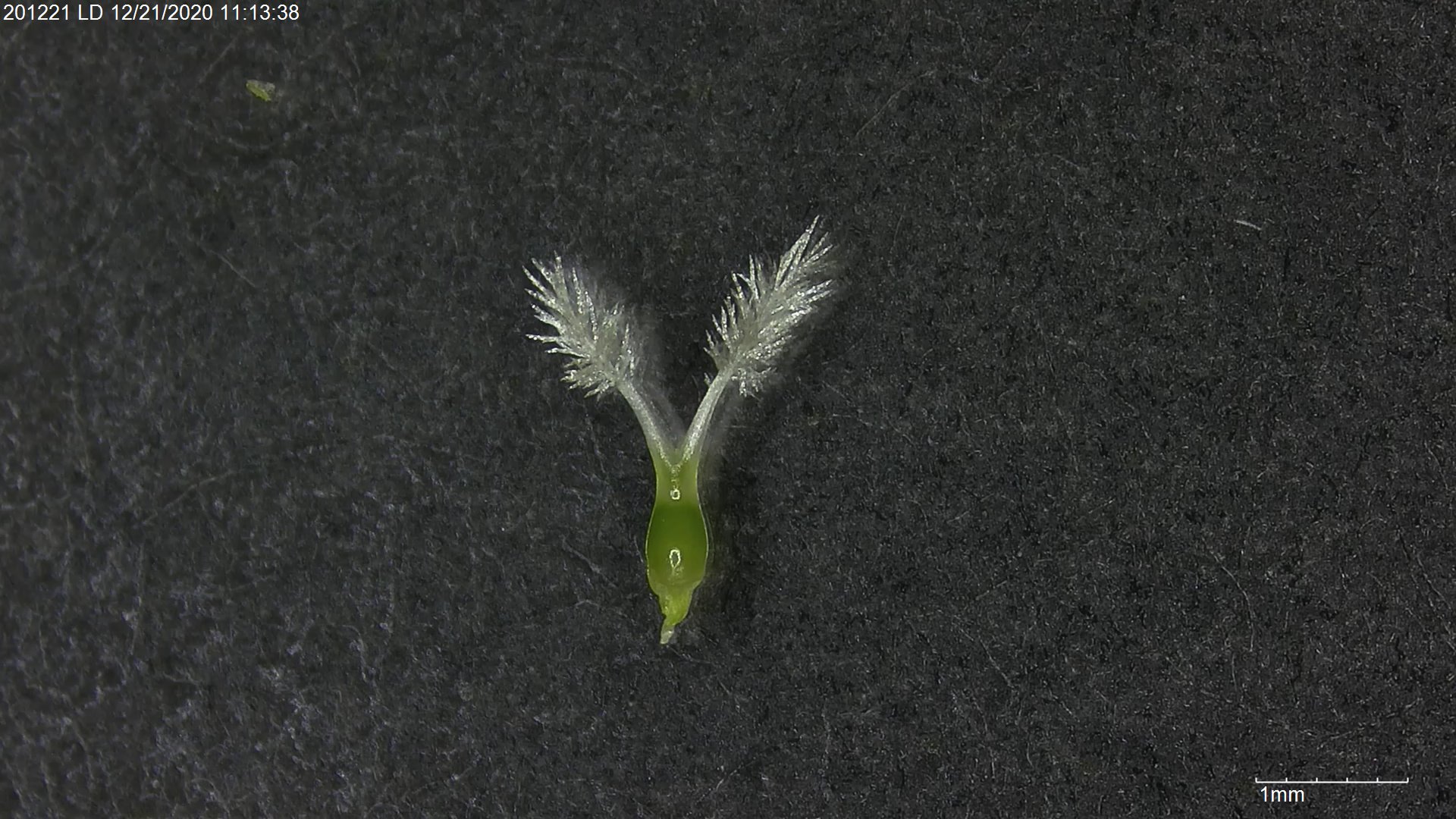

Supplement: Supplementary file 16 — Source_Data_zip [file 41467_2023_38881_MOESM16_ESM.zip › Source_Data_files_RK/Sup Fig_2/SupFig2_b_LD NB pistil.jpg]

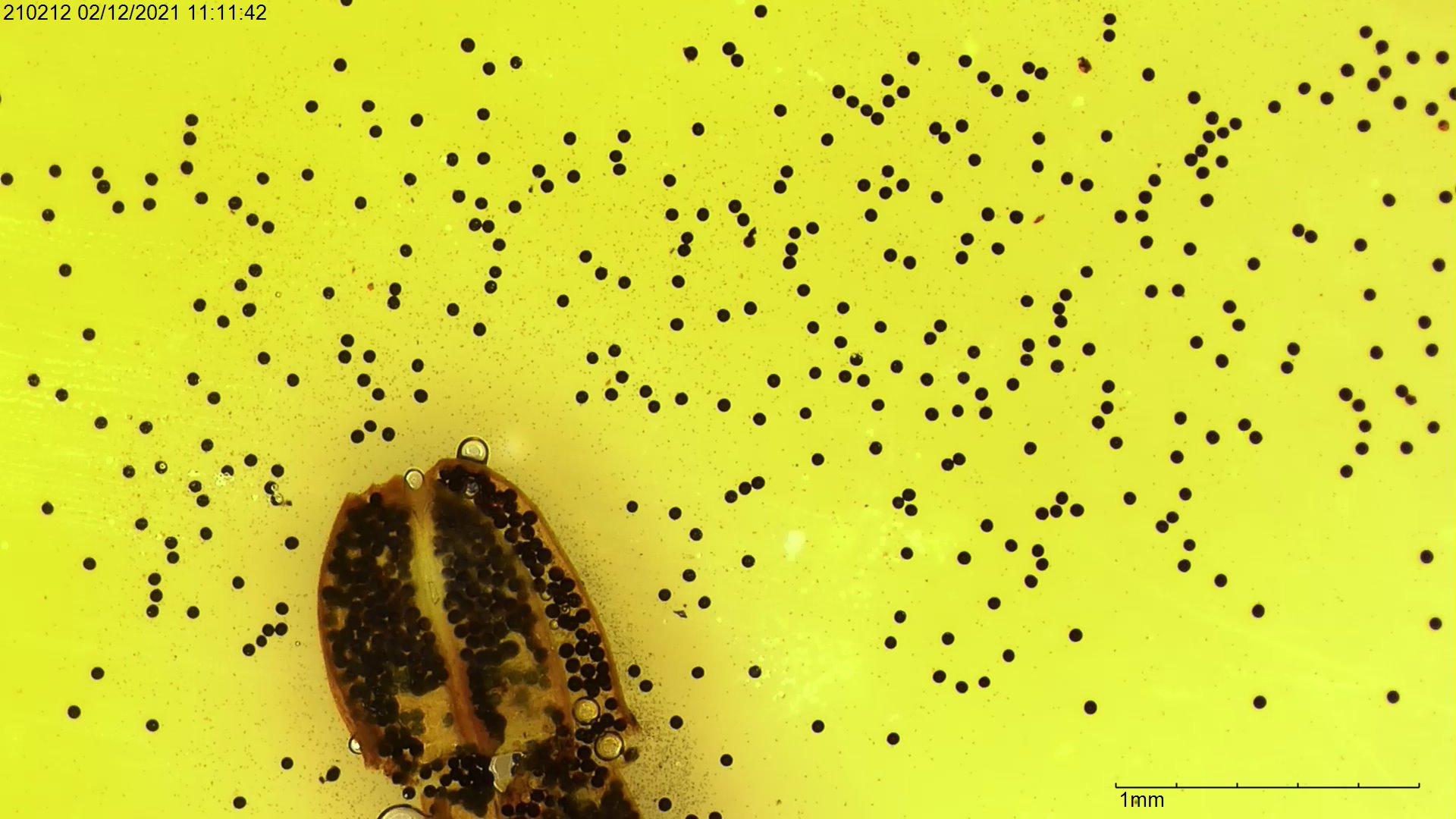

Supplement: Supplementary file 16 — Source_Data_zip [file 41467_2023_38881_MOESM16_ESM.zip › Source_Data_files_RK/Sup Fig_2/SupFig2_b_LD b-1-12 pollen_x2-3.jpg]

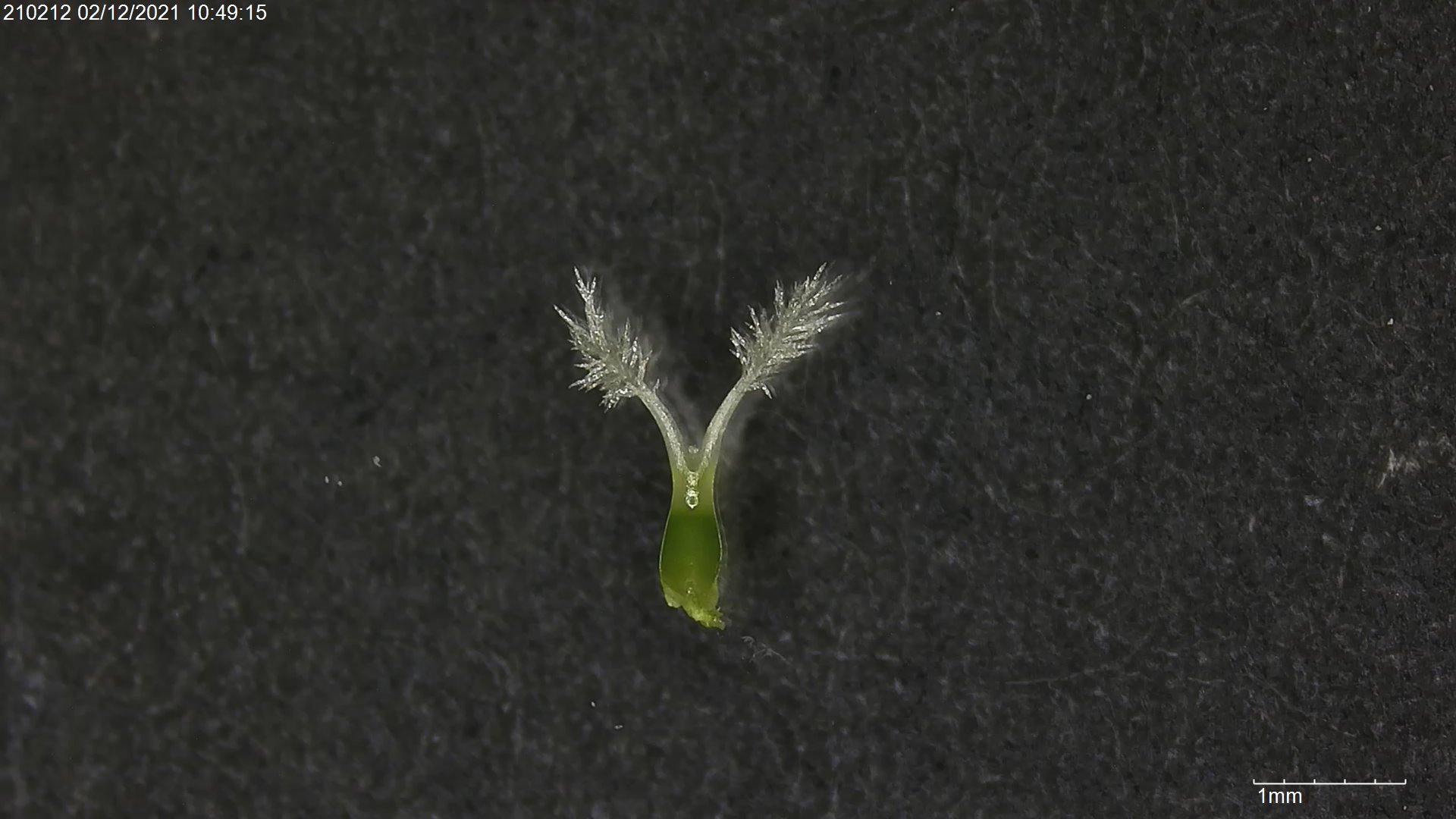

Supplement: Supplementary file 16 — Source_Data_zip [file 41467_2023_38881_MOESM16_ESM.zip › Source_Data_files_RK/Sup Fig_2/SupFig2_b_LD b-1-12 pistil_3.jpg]

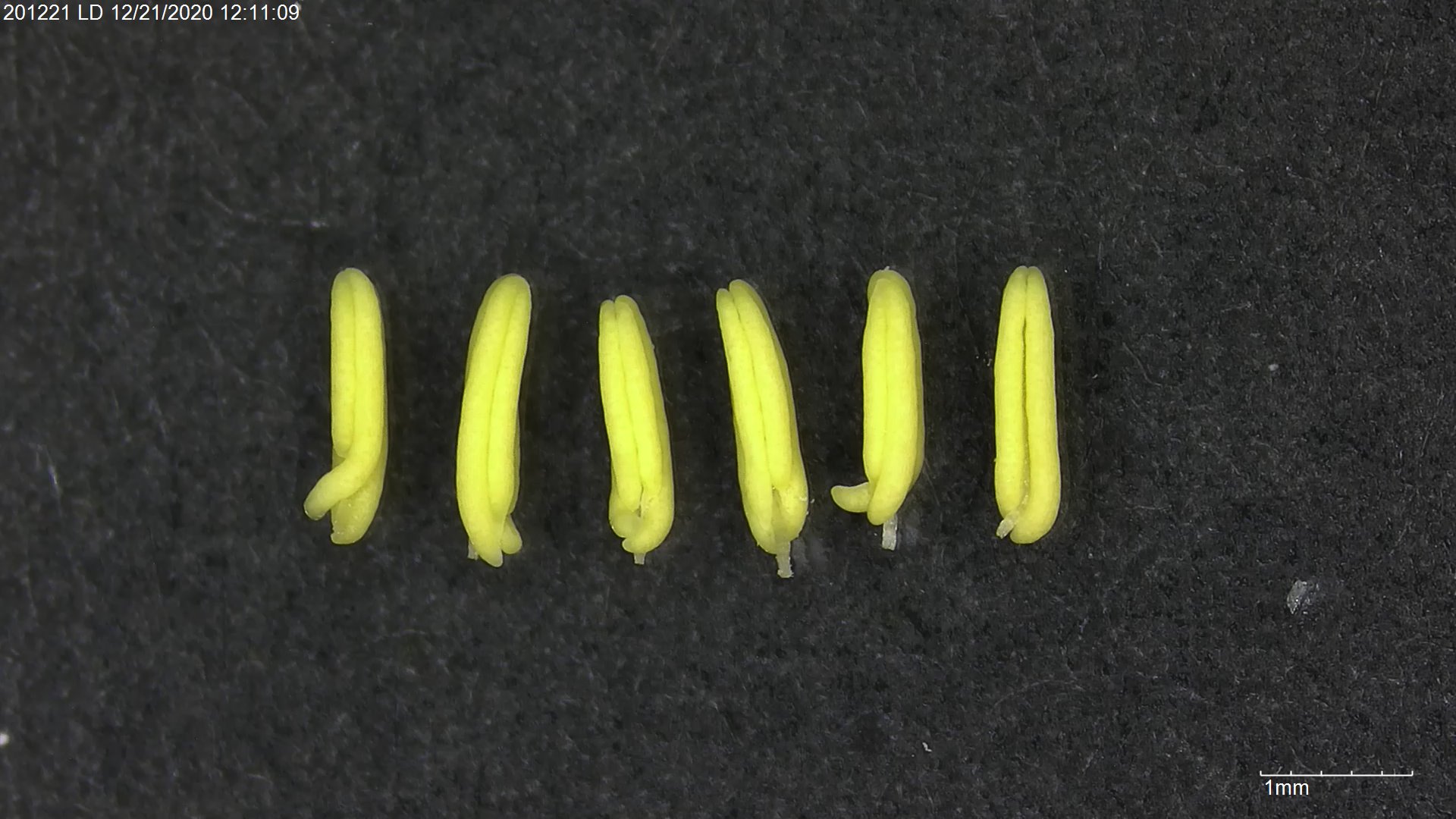

Supplement: Supplementary file 16 — Source_Data_zip [file 41467_2023_38881_MOESM16_ESM.zip › Source_Data_files_RK/Sup Fig_2/SupFig2_b_LD d g4-3-7-2 20dele anther2.jpg]

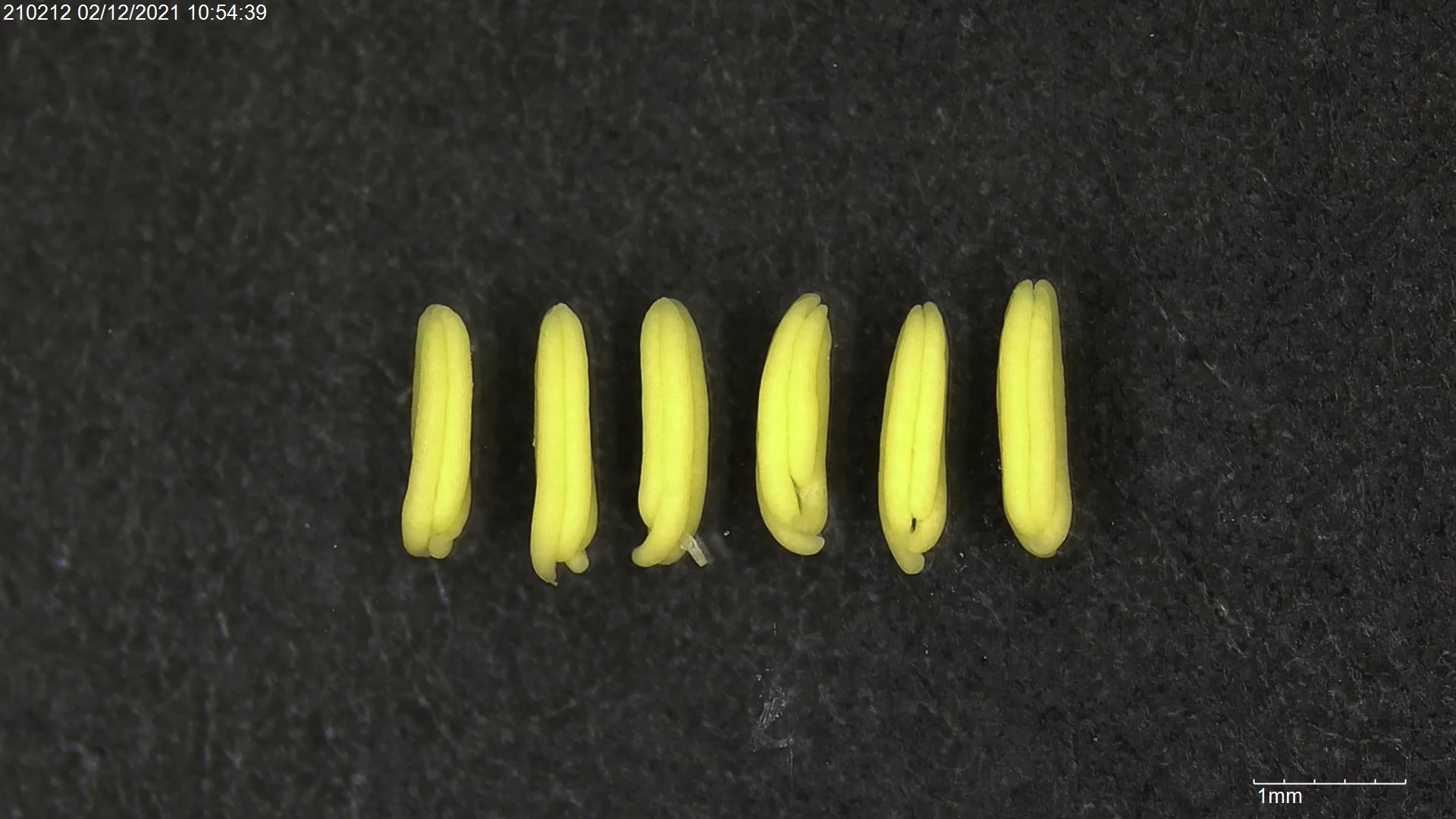

Supplement: Supplementary file 16 — Source_Data_zip [file 41467_2023_38881_MOESM16_ESM.zip › Source_Data_files_RK/Sup Fig_2/SupFig2_b_LD b-1-12 anther_2.jpg]

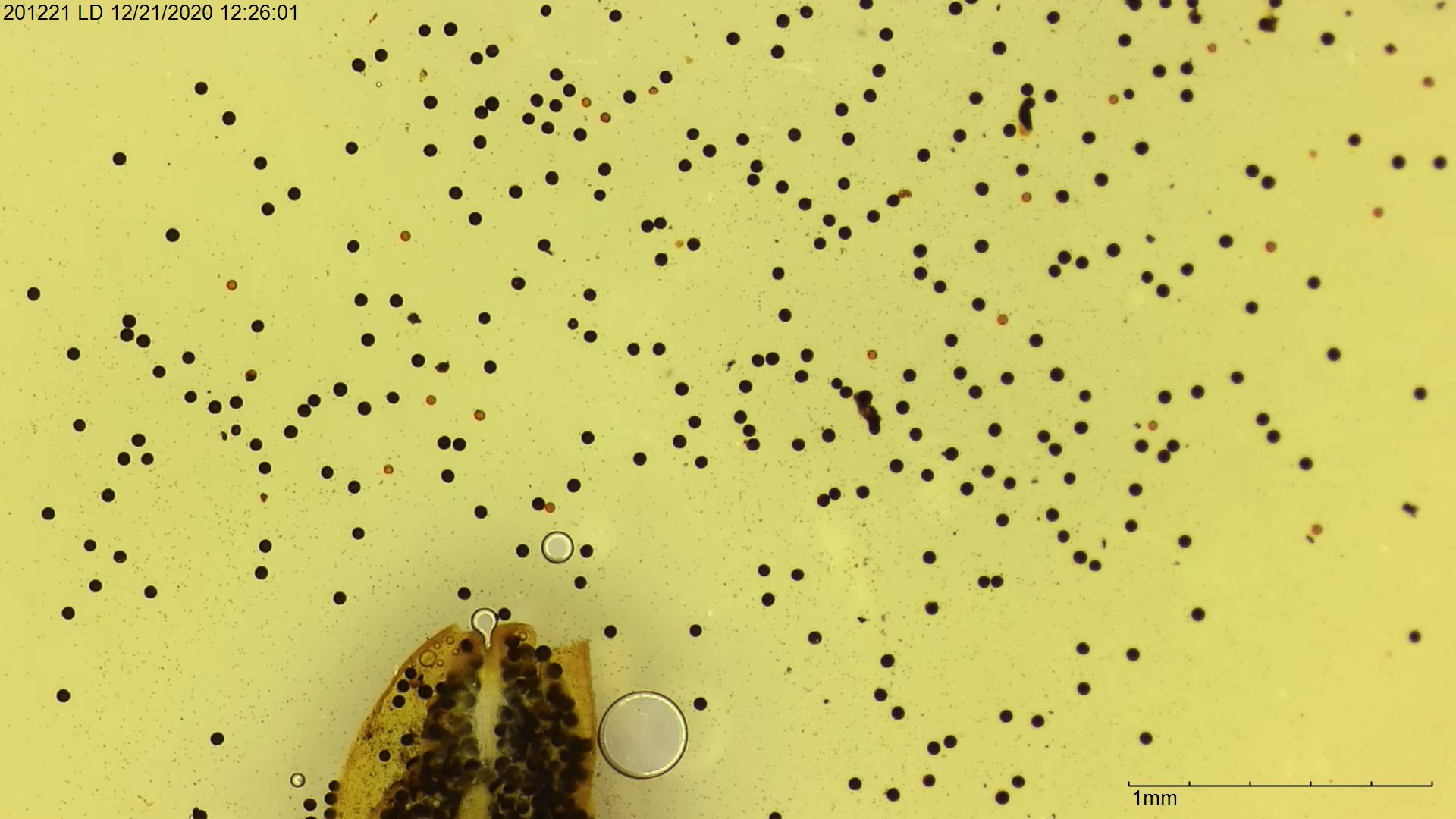

Supplement: Supplementary file 16 — Source_Data_zip [file 41467_2023_38881_MOESM16_ESM.zip › Source_Data_files_RK/Sup Fig_2/SupFig2_b_LD d g4-3-7-2 20dele pollenx2-3.jpg]

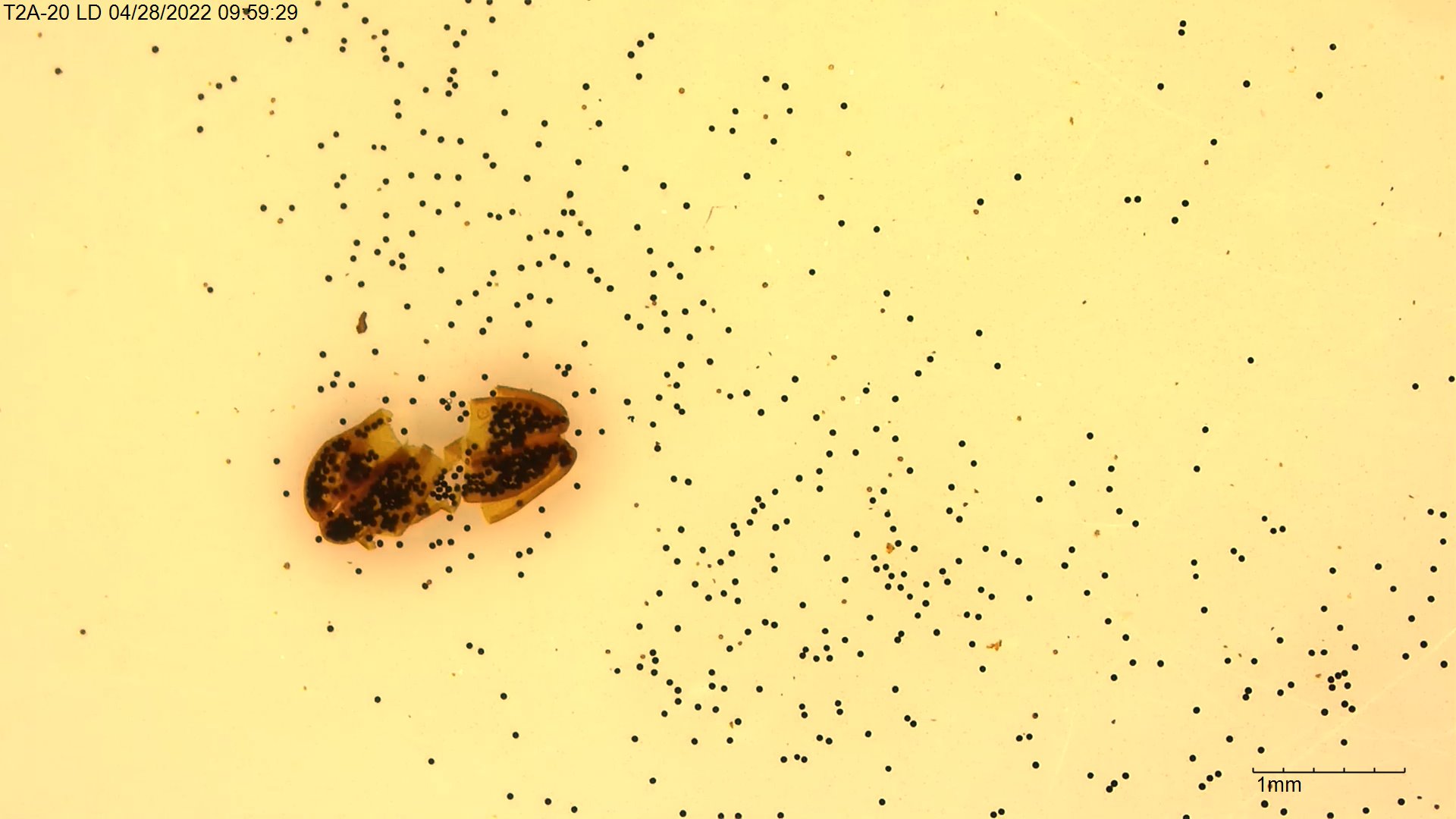

Supplement: Supplementary file 16 — Source_Data_zip [file 41467_2023_38881_MOESM16_ESM.zip › Source_Data_files_RK/Sup Fig_3/SupFig3_b_T2A-20 LD WT pollen96-1x1.jpg]

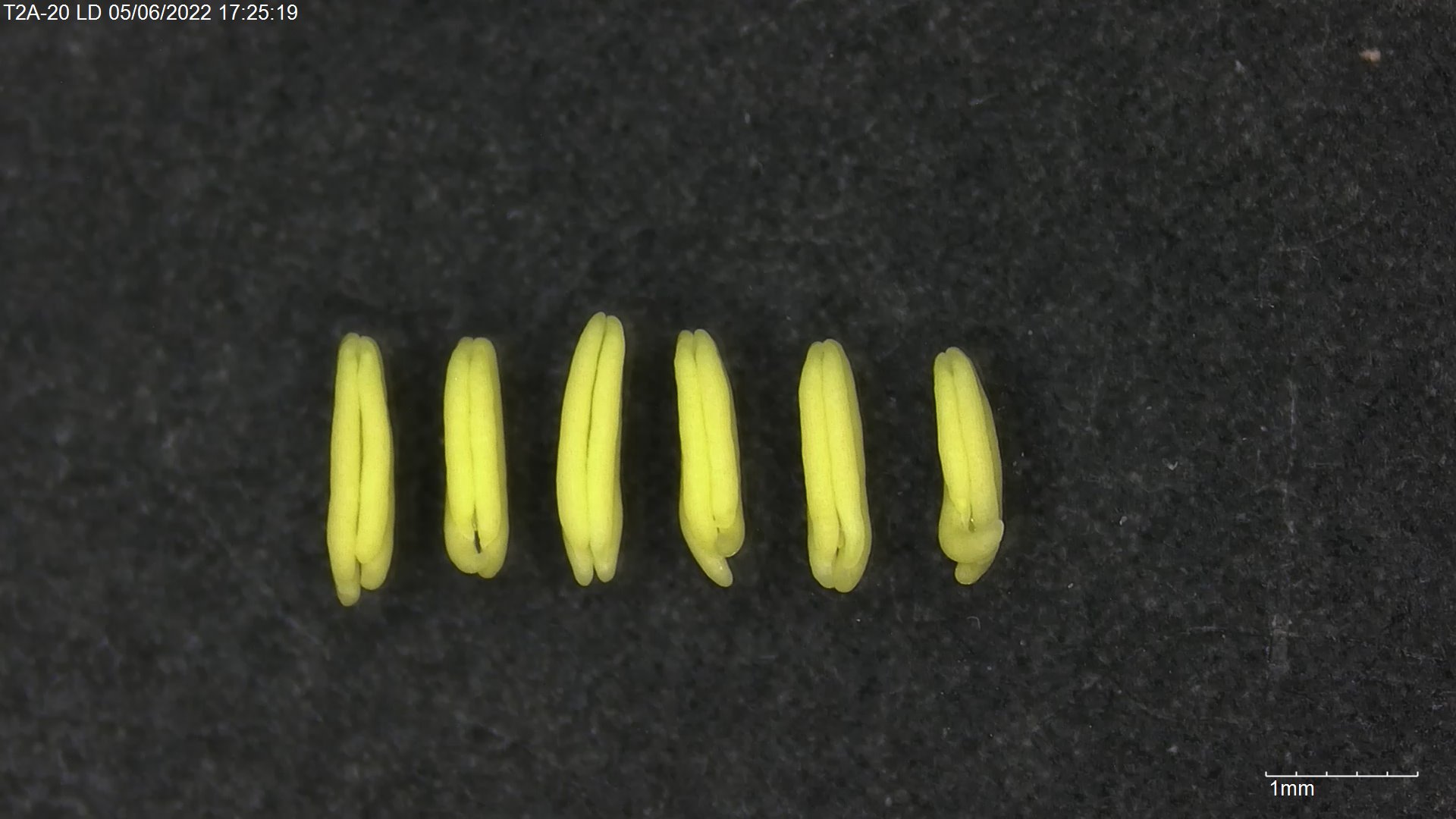

Supplement: Supplementary file 16 — Source_Data_zip [file 41467_2023_38881_MOESM16_ESM.zip › Source_Data_files_RK/Sup Fig_3/SupFig3_a_T2A-20 LD WT anther96-3.jpg]

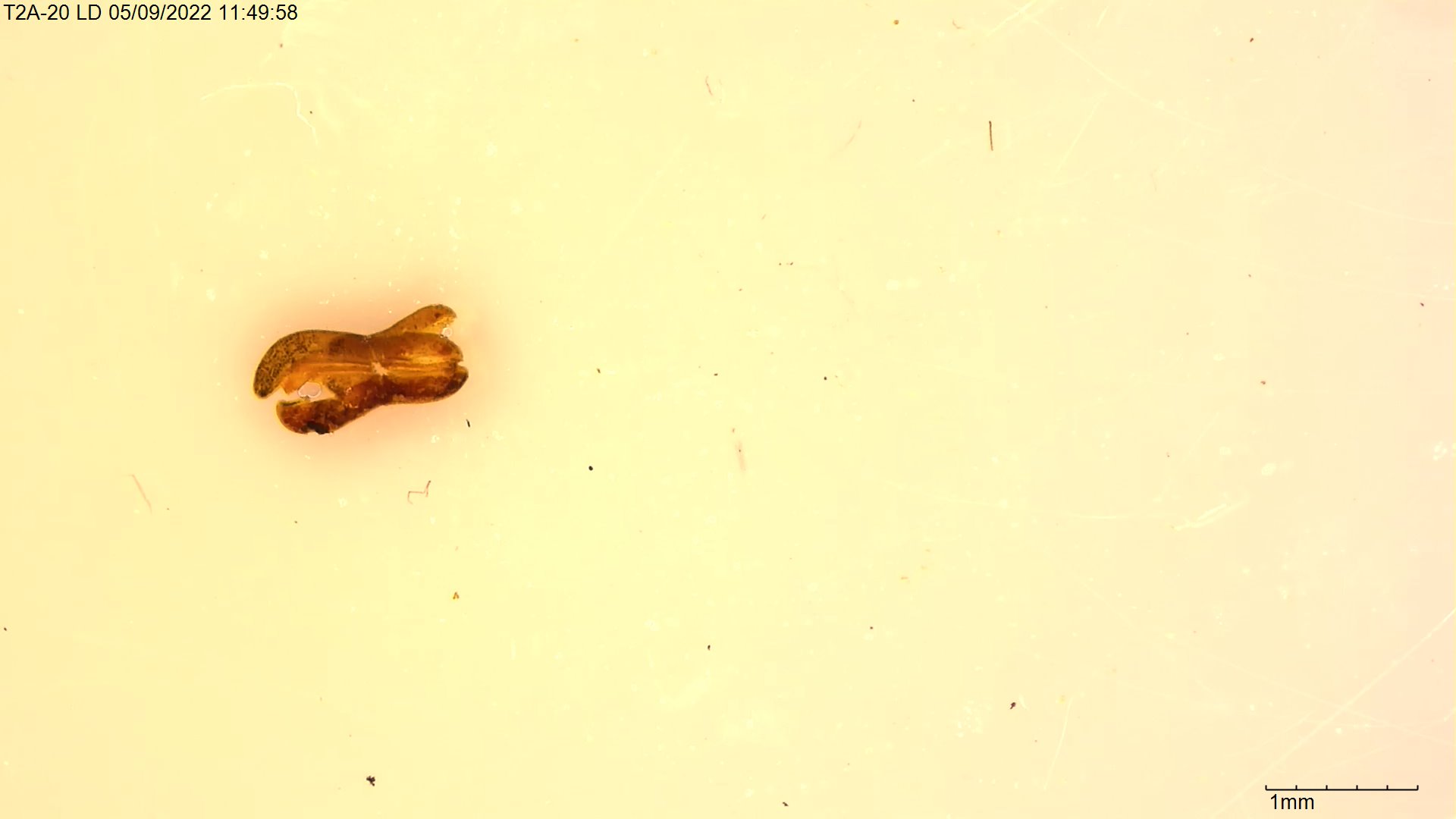

Supplement: Supplementary file 16 — Source_Data_zip [file 41467_2023_38881_MOESM16_ESM.zip › Source_Data_files_RK/Sup Fig_3/SupFig3_d_T2A-20 LD aa anther75-1.jpg]

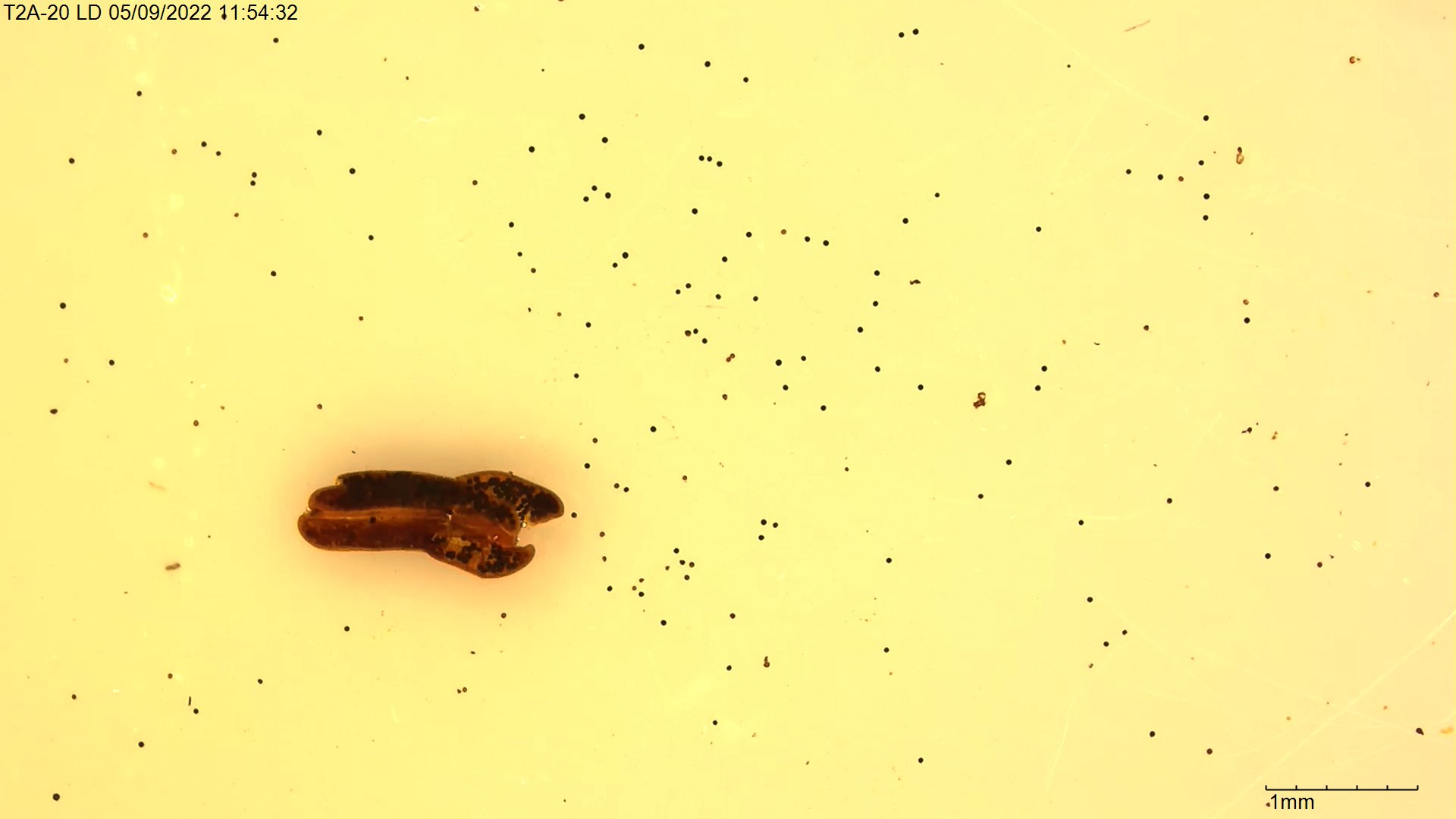

Supplement: Supplementary file 16 — Source_Data_zip [file 41467_2023_38881_MOESM16_ESM.zip › Source_Data_files_RK/Sup Fig_3/SupFig3_e_T2A-20 LD aa anther75-2.jpg]

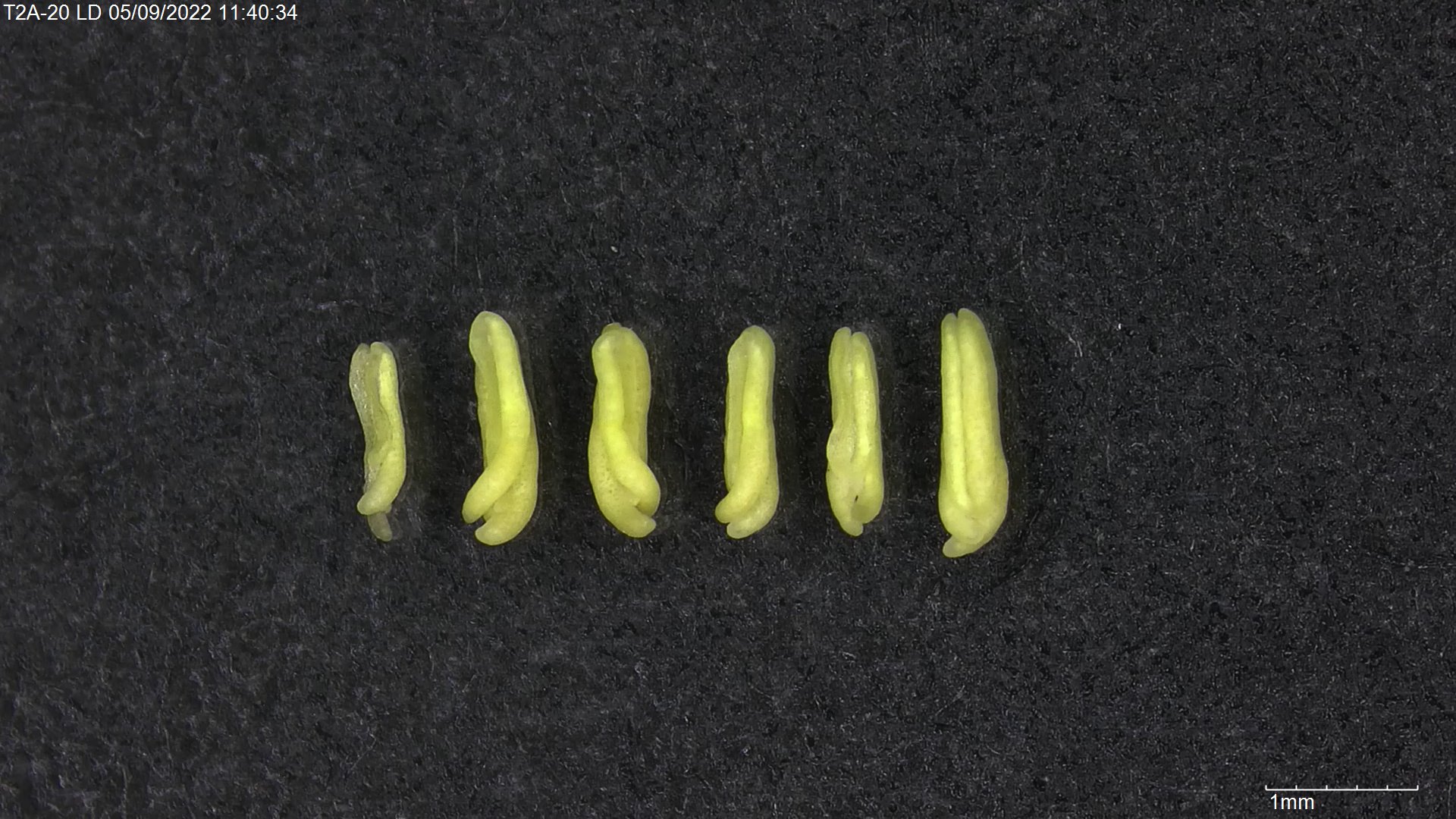

Supplement: Supplementary file 16 — Source_Data_zip [file 41467_2023_38881_MOESM16_ESM.zip › Source_Data_files_RK/Sup Fig_3/SupFig3_c_T2A-20 LD aa anther75.jpg]

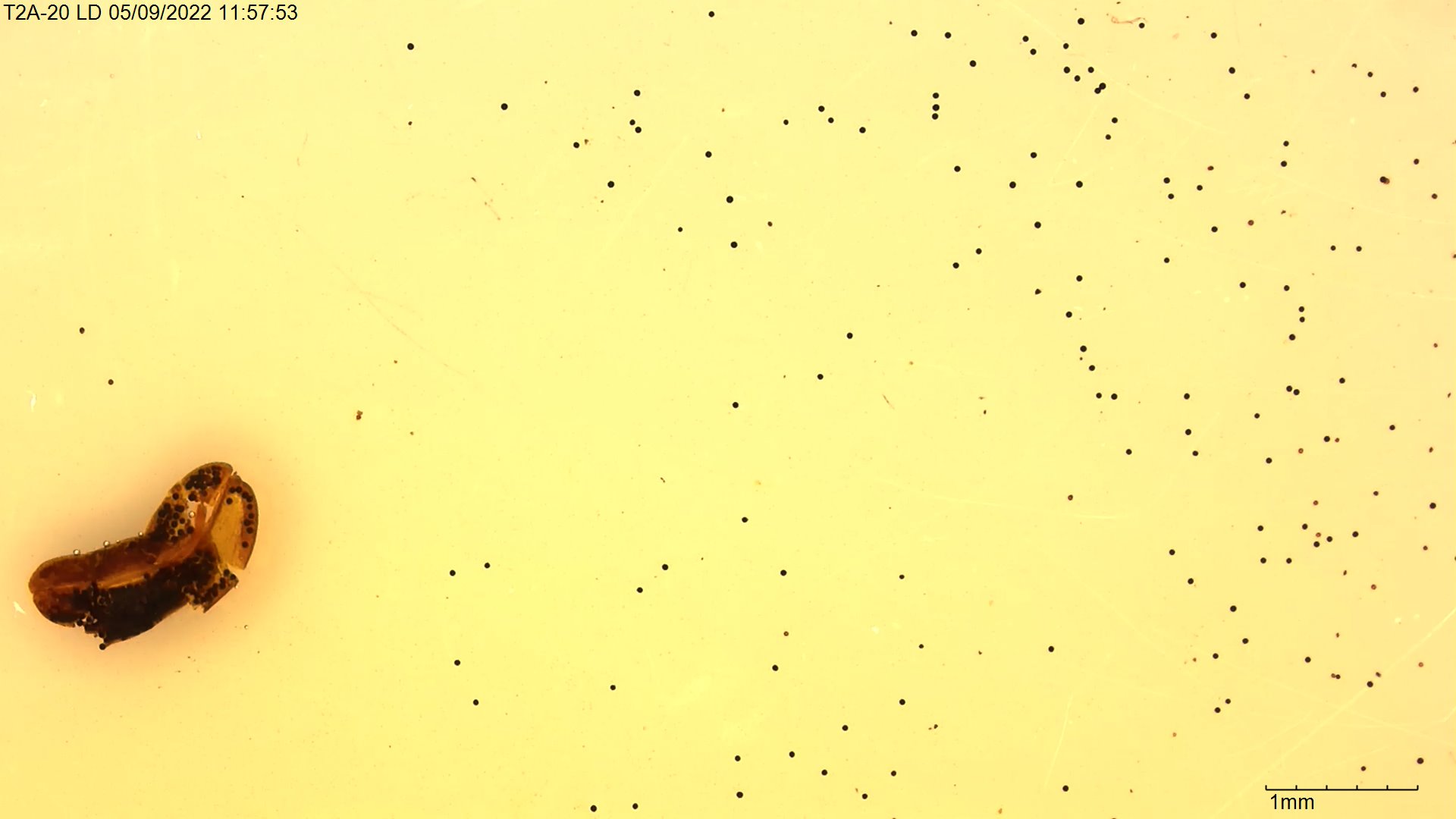

Supplement: Supplementary file 16 — Source_Data_zip [file 41467_2023_38881_MOESM16_ESM.zip › Source_Data_files_RK/Sup Fig_3/SupFig3_f_T2A-20 LD aa anther75-3.jpg]

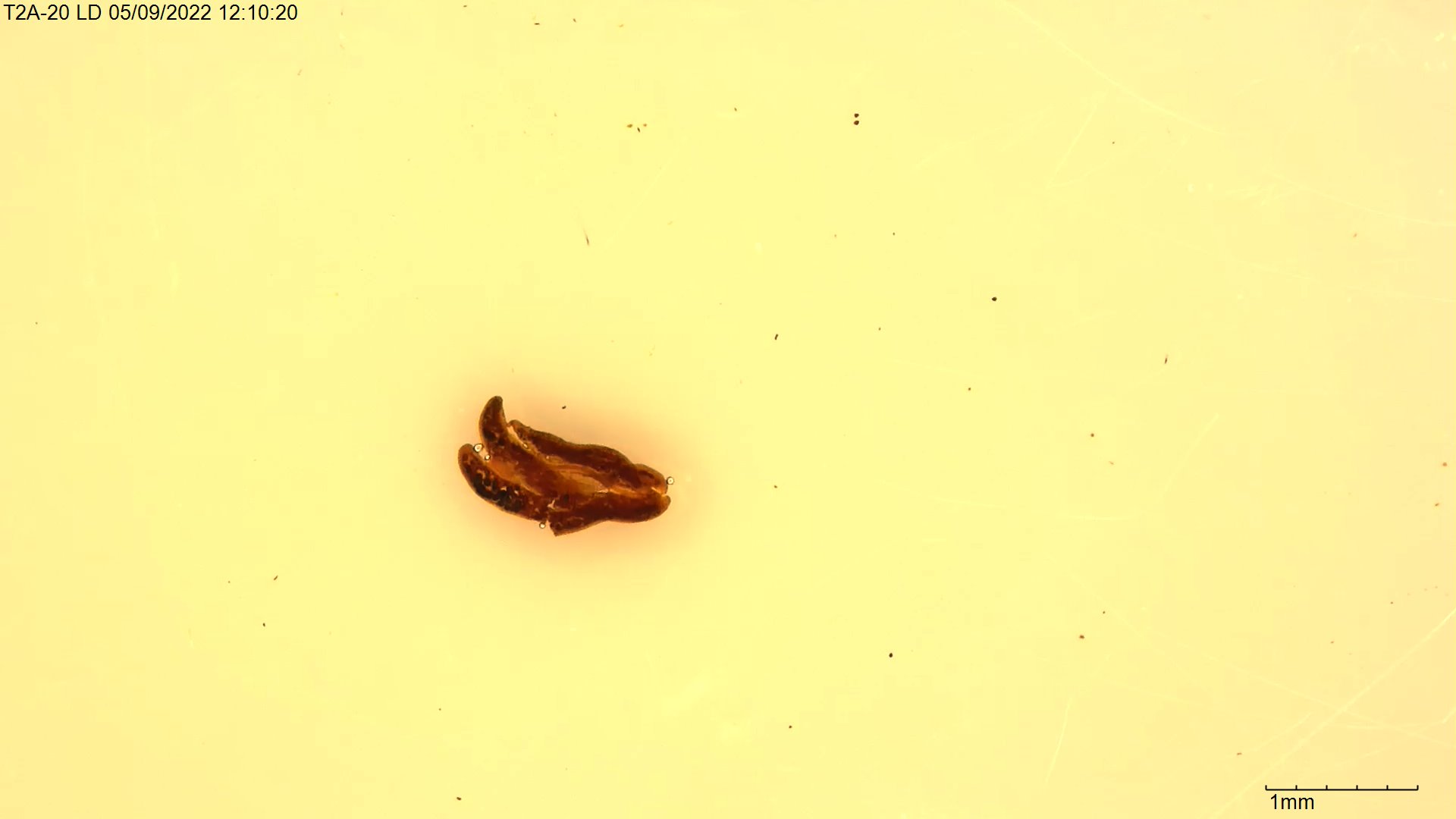

Supplement: Supplementary file 16 — Source_Data_zip [file 41467_2023_38881_MOESM16_ESM.zip › Source_Data_files_RK/Sup Fig_3/SupFig3_i_T2A-20 LD aa anther75-6.jpg]

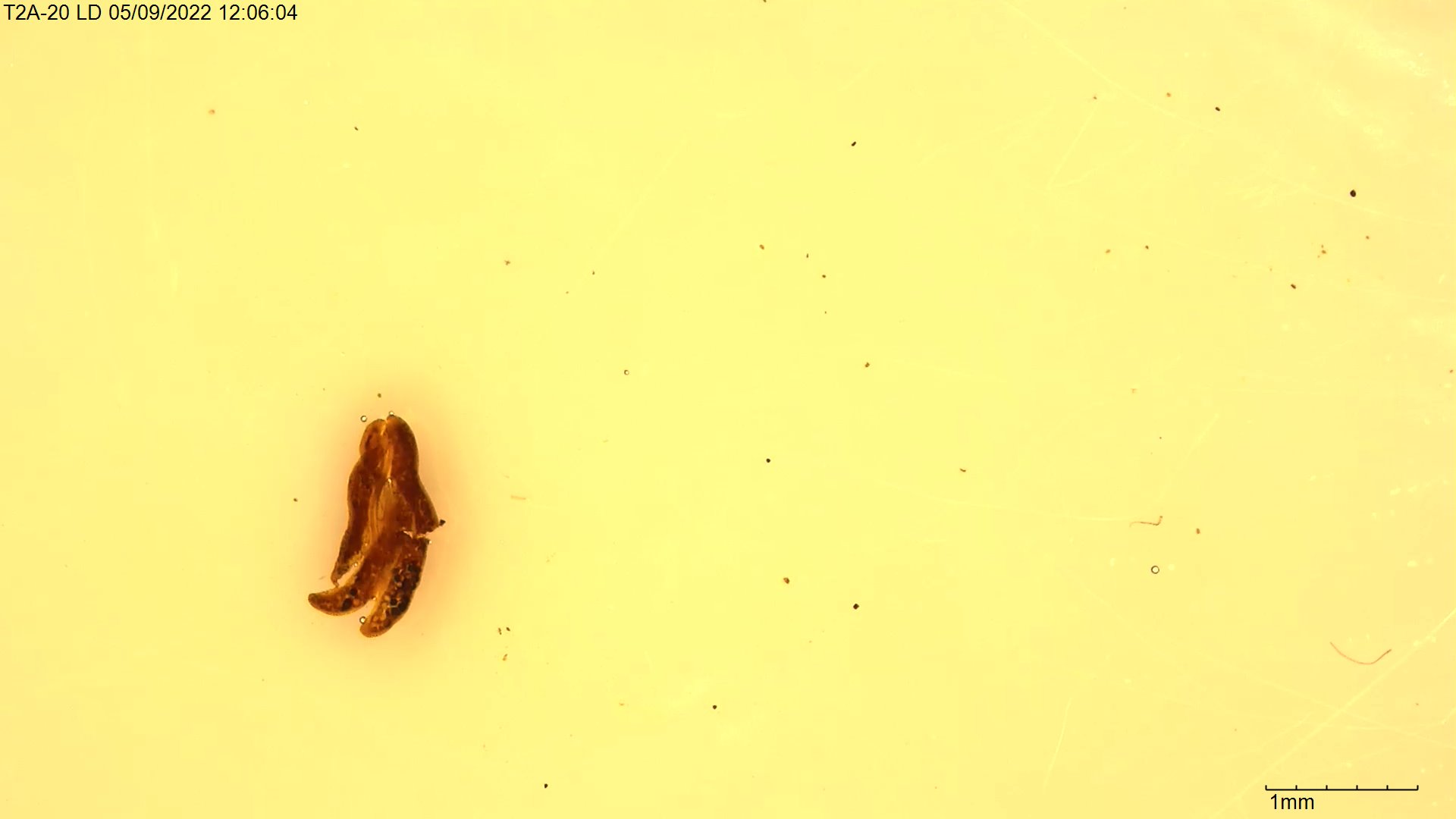

Supplement: Supplementary file 16 — Source_Data_zip [file 41467_2023_38881_MOESM16_ESM.zip › Source_Data_files_RK/Sup Fig_3/SupFig3_h_T2A-20 LD aa anther75-5.jpg]

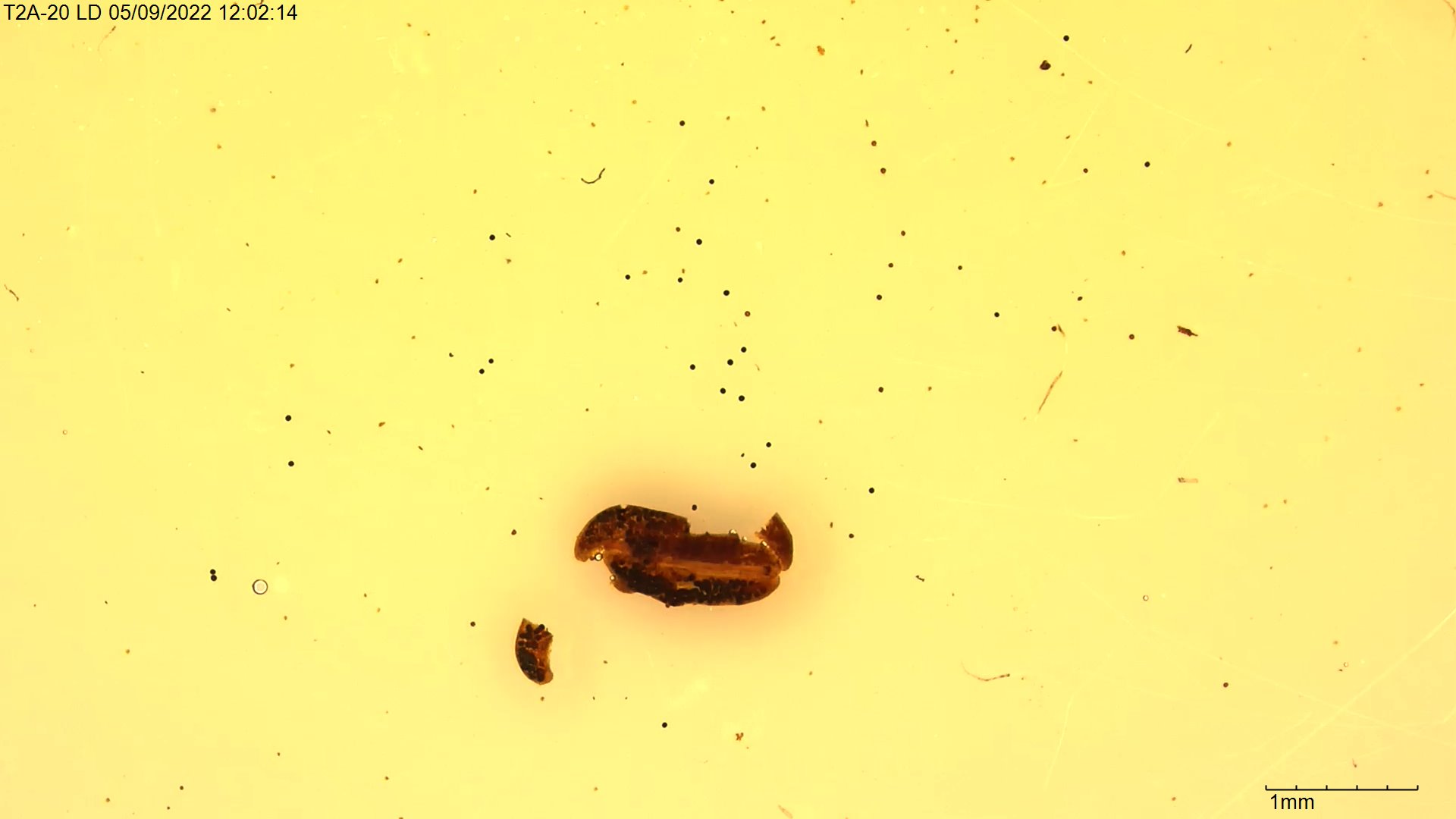

Supplement: Supplementary file 16 — Source_Data_zip [file 41467_2023_38881_MOESM16_ESM.zip › Source_Data_files_RK/Sup Fig_3/SupFig3_g_T2A-20 LD aa anther75-4.jpg]
